# Improved Statistics for Genome-Wide Interaction Analysis

**DOI:** 10.1371/journal.pgen.1002625

**Published:** 2012-04-05

**Authors:** Masao Ueki, Heather J. Cordell

**Affiliations:** 1 Faculty of Medicine, Yamagata University, Yamagata, Japan; 2 Institute of Genetic Medicine, Newcastle University, Newcastle upon Tyne, United Kingdom; University of California San Diego and The Scripps Research Institute, United States of America

## Abstract

Recently, Wu and colleagues [1] proposed two novel statistics for genome-wide interaction analysis using case/control or case-only data. In computer simulations, their proposed case/control statistic outperformed competing approaches, including the *fast-epistasis* option in PLINK and logistic regression analysis under the correct model; however, reasons for its superior performance were not fully explored. Here we investigate the theoretical properties and performance of Wu et al.'s proposed statistics and explain why, in some circumstances, they outperform competing approaches. Unfortunately, we find minor errors in the formulae for their statistics, resulting in tests that have higher than nominal type 1 error. We also find minor errors in PLINK's *fast-epistasis* and *case-only* statistics, although theory and simulations suggest that these errors have only negligible effect on type 1 error. We propose adjusted versions of all four statistics that, both theoretically and in computer simulations, maintain correct type 1 error rates under the null hypothesis. We also investigate statistics based on correlation coefficients that maintain similar control of type 1 error. Although designed to test specifically for interaction, we show that some of these previously-proposed statistics can, in fact, be sensitive to main effects at one or both loci, particularly in the presence of linkage disequilibrium. We propose two new “joint effects” statistics that, provided the disease is rare, are sensitive only to genuine interaction effects. In computer simulations we find, in most situations considered, that highest power is achieved by analysis under the correct genetic model. Such an analysis is unachievable in practice, as we do not know this model. However, generally high power over a wide range of scenarios is exhibited by our joint effects and adjusted Wu statistics. We recommend use of these alternative or adjusted statistics and urge caution when using Wu et al.'s originally-proposed statistics, on account of the inflated error rate that can result.

## Author Summary

Gene–gene interactions are a topic of great interest to geneticists carrying out studies of how genetic factors influence the development of common, complex diseases. Genes that interact may not only make important biological contributions to underlying disease processes, but also be more difficult to detect when using standard statistical methods in which we examine the effects of genetic factors one at a time. Recently a method was proposed by Wu and colleagues [1] for detecting pairwise interactions when carrying out genome-wide association studies (in which a large number of genetic variants across the genome are examined). Wu and colleagues carried out theoretical work and computer simulations that suggested their method outperformed other previously proposed approaches for detecting interactions. Here we show that, in fact, the method proposed by Wu and colleagues can result in an over-preponderence of false postive findings. We propose an adjusted version of their method that reduces the false positive rate while maintaining high power. We also propose a new method for detecting pairs of genetic effects that shows similarly high power but has some conceptual advantages over both Wu's method and also other previously proposed approaches.

## Introduction

Genome-wide association studies (GWAS) have been remarkably successful at identifying the genomic locations of variants involved in a variety of complex diseases [2]–[7]. In spite of this success, some researchers have expressed disquiet at the issue of the ‘missing heritability’ [8], namely the fact that the disease-associated single nucleotide polymorphisms (SNPs) identified through GWAS often account for only a small proportion of the the observed correlations in phenotype between relatives. This suggests that additional genetic factors remain to be found. Several explanations for this phenomenon have been suggested. Firstly, the SNPs identified through GWAS are likely to be surrogates in (imperfect) linkage disequilibrium (LD) with the true causal variants, and thus cannot be expected to fully account for their effects, particularly if the true causal variants are rare. Secondly, the low power of GWAS to detect loci of small effect means that many specific true loci remain undiscovered, even though the fact of their (combined) existence may be detectable from the observed genetic data [9], [10]. Finally (and the main focus of this communication) is the fact that the single-locus (SNP by SNP) testing strategy generally undertaken as the primary analysis tool in a GWAS may be underpowered to detect loci that interact with other genetic or enviromental factors, since effects at such loci may not be visible unless the contributing interacting factors are also taken into account.

The relationship between *biological* and *statistical* interaction has been hotly debated over many years [11]–[19]. It is now generally accepted that the lack of direct correspondence between statistical and biologial interaction makes it difficult to make strong inferences concerning biological mechanism from the existence of interaction terms in a statistical model. Nevertheless, the existence of such terms does imply that the interacting factors should at least both be ‘involved’ in disease in some way. Detection of statistical interaction thus provides a good starting point for a more focussed investigation of the joint involvement of the relevant factors, which can perhaps be better addressed through other types of experimental data. In addition, the increased detection power provided by statistical models that include interaction terms, when such terms do in fact operate [20], motivates the development of improved methods for detecting and modelling statistical interaction, particularly in the context of GWAS. The hope is that such methods will be useful for detecting effects that may be missed in standard single-locus analysis, thus providing a complementary strategy to standard GWAS analysis approaches for detecting loci involved in disease.

In case/control studies, statistical interaction is generally modelled as departure from a simple linear model describing the individual (main) effects of predictor variables on the predicted log odds of disease [17]. Consider two binary variables, 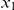 and 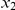, whose presence/absence (coded 0/1) is believed be associated with a disease outcome. Logistic regression models the main effects (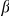 and 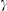) and interaction term (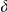) between the variables via the linear model
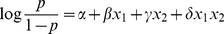
(1)where 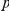 represents the probability that an individual in the study is a case rather than a control. Applying this idea to genetic predictor variables (such as SNP genotypes) is complicated by the fact that genetic predictors are not binary, but rather take 3 levels according to the number of copies (0,1,2) of the susceptibility allele possessed. However, we can easily convert to a binary coding by assuming a recessive or dominant model for each of the factors considered (thus collapsing two genotype categories to one at each locus). Alternatively, we can fit the above regression model using predictor variables coded (0,1,2), according to the number of susceptibility allele possessed, thus imposing an additive model (on the log odds scale) within each locus for the effect of each susceptibility allele. Yet another approach would be to fit a more general nine parameter (saturated) genotype model [17], that includes effects due to one or two copies of the susceptibility allele at locus 1 (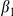, 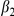), at locus 2 (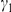, 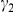), and four interaction parameters (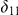, 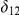, 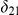, 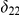) representing the additional contribution to risk from combinations of these effects, resulting in the following model:
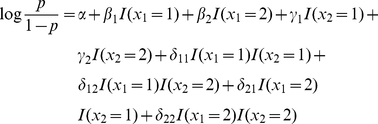
(2)(where here 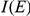 represents an indicator variable for the occurence of event 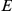).

Given the simpler logistic regression model (1), a variety of tests can be performed to assess the effects of the two contributing factors. (Similar tests can be derived for logistic regression model (2)). A 3df test of 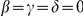 tests for association at both loci, allowing for their possible interaction. A 2df test of 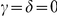 tests for association at locus 2, allowing for possible interaction with locus 1. Such a test has been shown to be a powerful approach when interactions exist, while losing very little power when no interactions exist [20]. In the current communication, we will focus on the 1df test of 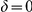 i.e. a test of the interaction term alone. This test has the disadvantage of being generally underpowered compared to tests of main effects [21]. However, we might hope that loci with reasonably large main effects will be potentially detectable via a single-locus scan. We are interested in detecting loci that will be missed via single-locus analysis, i.e. those for which the interaction term is likely to be particularly important. Moreover, assuming we can construct a good test of 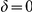, this test can potentially be combined with tests of the main effects [22], allowing the construction of joint tests of association while allowing for interaction, if desired.

## Methods

### Wu et al. (2010) statistic

Recently, Wu and colleagues [1] proposed two novel statistics for genome-wide (pairwise) interaction analysis using case/control or case-only data. The statistics proposed by [1] were based on considering ‘haplotypes’ at two diallelic loci, 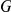 and 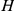, with locus 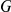 having alleles 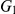 and 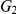 and locus 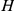 having alleles 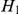 and 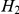. For linked loci, the concept of ‘haplotype’ corresponded to its usual interpretation in terms of the physical coupling of alleles on the DNA strand inherited from a single parent. For unlinked loci, the concept of ‘haplotype’ referred simply to the fact the alleles involved were inherited from the same parent (a concept sometimes referred to as gametic phase disequilibrium), without necessarily implying any physical coupling of the alleles.

Wu et al. [1] propose to detect interaction via consideration of the log odds ratio
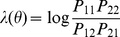
(3)where 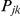 is the haplotype frequency of haplotype 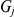
−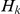 (i.e. the probability of this haplotype) in some sample under consideration. We define a parameter vector 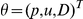, chosen to reparameterise the 4 haplotype frequencies 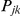 in terms of the allele frequencies, 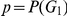, 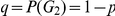, 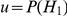, and 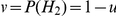, and a ‘linkage disequilibrium’ (LD) (or more generally, for unlinked loci, allelic association) parameter, 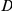, such that
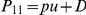


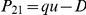


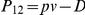


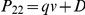
Note that the odds ratio 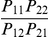 in (3) relates to the odds of a 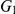 allele appearing on a ‘haplotype’ in coupling with a 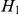 allele (and a 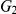 allele with 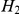) i.e. it acts a measure of correlation between alleles at the two loci, rather than relating to the odds of disease. No correlation (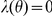) corresponds to the situation where the allelic association parameter 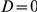. Wu et al. (2010) propose that, under the null hypothesis of no interaction, 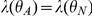, where 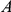 and 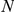 refer to calculating 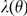 within the sample of cases and controls respectively. If, in addition, there is no population-level allelic association between alleles at 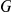 and 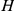, then 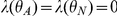.

Wu et al. [1] give a complicated description motivating their use of 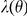, however this quantity can perhaps more easily be motivated by analogy with classical ‘case-only’ analysis [23], [24], [25]. Case-only analysis stems from the observation that, for binary predictor variables, a test of the interaction term 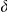 in the logistic regresssion model (1) can be obtained by noticing that it equals the ‘ratio of odds ratios’:
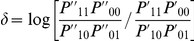
where 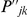 and 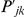 are the joint probabilities that binary variables 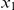 and 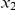 take values 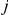 and 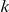, i.e. 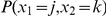, calculated within the sample of cases and controls, respectively. If variables 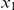 and 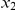 are uncorrelated in the controls (or, equivalently, in the general population under a rare disease assumption) then the denominator
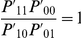
and a test of interaction can be constructed by testing whether
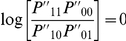
This test has the advantage [23], [24], [25] of being substantially more powerful than the usual logistic regression test of 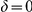. If we are not willing to assume that variables 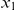 and 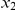 are uncorrelated in the controls, then a natural test of interaction can instead be constructed by testing whether
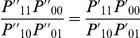
or, equivalently, whether
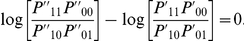
Considered in this light, the log odds ratio 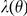 considered by Wu et al. [1] can be seen as analagous to the quantity used in case-only analysis, if the unit of analysis is defined to be a ‘haplotype’ (rather than an individual) and if binary variables 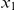 and 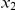 are defined as indicator variables for the two possible alleles at each locus on the haplotype.

To test for interaction, Wu et al. [1] propose two 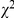 test statistics, one for case-only and one for case/control analysis, which we denote as 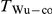 and 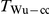


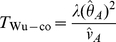
(4)

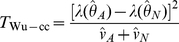
(5)Here 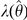 is the log OR (for 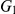
−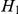 and 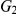
−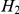 alleles being in coupling, as opposed to 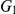
−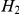 and 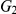
−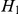), 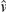 is its estimated variance (calculated using the delta method), and 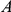 and 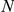 refer to quantities calculated within the sample of cases and controls respectively. The case-only test should be suitable provided there is no correlation (e.g. due to LD) between alleles at the two loci. The case/control test is more suitable if we expect correlation between alleles at the two loci due to the fact they are linked, or induced by other influences such population stratification [26]).

In order to actually calculate 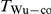 and 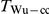, we need to know (or estimate) the ‘haplotype’ frequencies 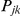 in cases and controls. Even for linked loci, haplotypes are not generally observed, but luckily many programs exist to estimate haplotype frequencies (often via an EM algorithm) given unphased genotype data. Most if not all such programs assume Hardy-Weinberg equilibrium (HWE) in order to perform the calculation. We expect HWE to hold in the general population (and thus in controls, under a rare disease assumption). Under the global null hyothesis of no association between disease status and the loci in question (via either main effects or interactions), haplotype frequencies in cases should be identical to those in controls, and HWE should also hold in the cases. However, under the alternative hypothesis of association and/or interaction, HWE will not necessarily hold in the cases [27] (unless the disease model is assumed to result from multiplicative haplotype effects [28]), meaning that haplotypes in cases cannot be considered to come together independently. We return to this point later.

Wu et al. [1] provide the following formulae for their proposed statistics:
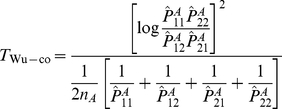
(6)

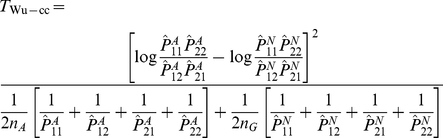
(7)where 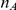 and 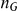 are the number of sampled case and control individuals, and 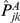 and 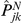 are estimators of the haplotype frequencies in cases and controls, respectively. However, the denominators in these formulae (based on calculating the asymptotic variances of 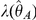 and 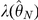) are only correct if haplotypes are actually observed i.e. there is no phase uncertainty. Consequently, we expect these variance estimates to be too small if haplotype frequencies are estimated from unphased genotype data, resulting in test statistics that are too large. In Text S1 we use results from Brown [29] and application of the delta method to calculate the correct asymptotic variances of 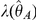 and 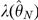. We refer to our corresponding resulting test statistics as ‘adjusted’ Wu statistics:
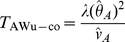
(8)

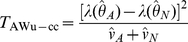
(9)(where 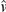 now relates to the correct asymptotic variance of 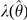 as given in Text S1). Interestingly, if one calculates this variance under the null hypothesis that 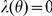 (as might be reasonable when performing case-only analysis, where this assumption is in any case required), it turns out that the resulting variance is exactly double that derived by Wu et al. [1]. In these circumstances, our case-only statistic 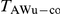 would be exactly half of the original Wu case-only statistic. This suggests that another way to construct a valid version of Wu's case-only statistic would be to simply divide the original statistic by two. In computer simulations (data not shown), we found negligible differences between between our ‘adjusted’ statistic 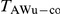 and 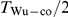, and thus, in our Results section, we only report results for 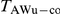.

### PLINK's *fast-epistasis* statistics

Two fast approaches for testing interaction (in addition to a slower logistic regression based approach) are implemented in the computer program PLINK [30]. For a set of individuals (either cases or controls), PLINK takes unphased genotype data as shown in Table 1 and expands it out to the 2
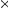
2 allelic table shown in Table 2. The log odds ratio 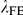 in this table can be calculated as 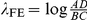 with estimated variance 
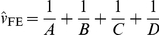
. PLINK's *fast-epistasis* tests test whether correlation between alleles at the two loci exists (case-only test) or is different between cases and controls (case/control test) via the following 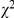 test statistics:
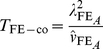
(10)

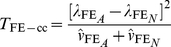
(11)Here 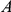 and 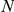 again refer to quantities calculated within the sample of cases and controls respectively. These statistics are seen to have exactly the same form as the Wu and adjusted Wu statistics, but with the log odds ratio 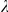 and its estimated variance relating to slightly different quantities, namely those quantities shown in Table 2.

**Table 1:** Multilocus genotype counts at two SNPs in a set of genotyped individuals.

Locus G	Locus H		
	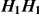	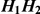	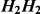
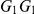	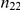	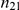	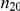
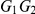	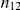	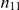	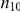
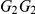	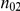	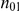	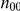

**Table 2:** Allele counts derived from Table 1, as calculated by PLINK.

Locus G	Locus H	
	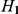	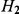
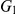	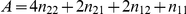	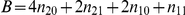
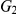	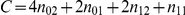	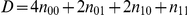

Apart from the difference in 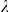, the main difference between PLINK's statistics and those proposed by Wu et al. is that fact that, in PLINK, no estimation of phased haplotype frequencies is performed. Nevertheless, the log odds ratio 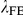 can be shown to be exactly that which would be obtained if one did estimate haplotype counts, assuming that the middle cell (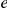) in Table 1 resolves into phased genotypes 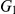
−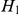/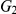
−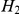 or 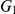
−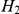/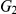
−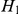 with equal frequencies. The haplotype counts implicitly utilized by PLINK are therefore similar to what would be obtained from an EM algorithm, except that in PLINK the middle cell is resolved assuming no correlation between alleles at the two loci, resulting (presumably) in a set of estimated haplotype frequencies that will be biased towards showing lower levels of allelic association. We hypothesise that this bias towards lower levels of allelic association might partly account for the inferior performance of PLINK observed by Wu et al. [1].

Although the log odds ratio 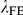 in PLINK corresponds to what would be obtained from attempting to resolve phase while assuming no correlation between alleles at the two loci, the variance estimate 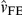 is based on counting 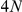 independent alleles rather than 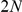 haplotypes (where 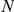 is the total number of individuals in Table 1). The formula for the variance estimate 
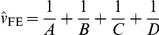
 assumes that there are 3 independent cell probabilities in Table 2. However, since the data in Table 2 was originally derived from Table 1, considering these data as realisations from a multinomial distribution, we can see that in fact there should be 8 parameters corresponding to 8 independent cell probabilities. In Text S1, we use the delta method to calculate the correct asymptotic variances of 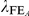 and 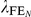, based on the multinomial data in Table 1. We refer to the corresponding resulting test statistics as ‘adjusted’ *fast-epistasis* statistics:
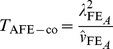
(12)

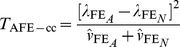
(13)where 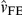 now relates to the correct asymptotic variance of 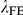 as given in Text S1, and 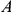 and 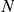 again refer to quantities calculated within the sample of cases and controls respectively.

### Wellek and Ziegler (2009) statistics

Both the methods of Wu et al. [1] and the *fast-epistasis* tests implemented in PLINK operate by turning a question about *statistical interaction* into a question about *allelic association* (or correlation), namely, whether association between alleles two loci exists (case-only test) or is different between cases and controls (case/control test)). However, many different measures of allelic association (usually calculated for linked loci, and thus assumed to reflect LD) have been proposed. Arguably the most popular are Lewontin's 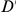 [31] and Pearson's product-moment correlation coefficient 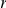 (or the square of it, 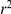) [32]. In most current genetic applications, these measures are calculated based on known or estimated haplotype frequencies. Wellek and Ziegler [33] pointed out that one advantage of 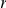 is that it may be calculated without estimating phase, simply by applying it to two variables, 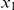 and 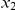, coded (0,1,2) according to the number of susceptibility alleles possessed at each locus. Wellek and Ziegler [33] examined the performance of 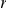 as a measure of LD using either estimated (phased) haplotype frequencies or using unresolved genotype data and showed that, if HWE holds, the loss of precision for estimating 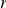 was negligible when using unphased genotypes rather than (phased) haplotypes.

If HWE does not hold, Wellek and Ziegler found the genotype-based estimator of 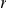 to be unbiased but the haplotype-based estimator to be strongly biased, i.e. to not reflect the ‘true’ value of 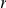 based on the true haplotype frequencies. This would seem an unappealing property of the haplotype-based estimator, if the goal is to accurately estimate the true level of allelic association (or LD) between two loci. However, if the purpose is rather to test for *interaction* (via testing whether correlation between alleles two loci exists (case-only test) or is different between cases and controls (case/control test)), it is possible that such a bias could be advantageous in terms of improving power. Since the method of Wu et al. [1] relies on estimating ‘haplotypes’ within the sample of cases (under a potentially incorrect HWE assumption), we hypothesise that the bias pointed out by [33] might also contribute to the superior performance observed by Wu et al. [1] for their approach compared to PLINK.

Given a genotype-based estimator of 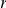, Wellek and Ziegler [33] propose using Fisher's 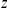 transformation to calculate a quantity
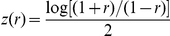
and its estimated variance. A natural pair of statistics for testing interaction based on 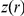 might therefore be:
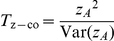


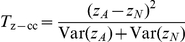
In computer simulations (data not shown), we found the performance of these statistics to be virtually identical to statistics based on the correlation coefficient itself. We therefore instead define our Wellek and Ziegler inspired statistics based on the correlation coefficient as:
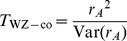
(14)

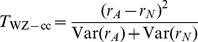
(15)where again 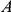 and 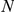 refer to quantities calculated within the sample of cases and controls respectively. Formulae for the correlation coefficient 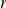 and its estimated variance are given by Wellek and Ziegler [33]. Note that the test based on the difference in the correlation coefficients between cases and controls, 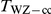, was also proposed by Kam-Thong et al. [34] and implemented in a program called EPIBLASTER. In EPIBLASTER, 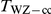 is used as a screening step, prior to performing a full logistic regression analysis on the subset of pairs of loci showing some loose level of significance with 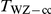.

### New “joint effects” tests

Although designed to test specifically for (statistical) interaction, several of the test statistics proposed above can be shown to be sensitive to the situation where there is, in fact, no interaction, but one or both of the loci display main effects (see details in Text S2). This is rather unsatisfactory as, even if one of the loci does have a genuine main effect, this phenomenon could lead to potentially increased false positive rates with respect to detection of the other locus (through its apparent – but false – interaction with the locus that has genuine main effects). Ideally, one would hope that detection of a significant interaction effect would indicate genuine interaction, but, even if this is not the case, one would at least hope that both loci identified have some involvement in disease (with their precise joint effects - interactive or otherwise - being determinable through further, more focussed, statistical or biological investigation).

In order to address this issue, we propose two new ‘joint effects’ tests that are sensitive only to either a) a genuine interaction effect or b) (if the disease is not sufficiently rare), main effects present at both loci. Our tests are motivated by a desire to test the same interaction parameter as tested by Wu et al. [1]. However, unlike some previously-proposed tests, our new tests can be shown to have the advantage of not being sensitive to main effects at a single locus. Moreover, under a rare disease assumption, our new tests can also be shown to be insensitive to main effects at both loci, thus reflecting genuine interaction. Thus, application of our joint effects tests will not result in an inflated type 1 error rate with respect to the *detection* of loci that are not involved in the disease (even though, for a common disease, our tests could potentially result in an inflated type 1 error with respect to whether the pair of loci actually interact, in the usual statistical sense).

Our new tests are based on the counts in Table 1, calculated separately within the sample of cases and controls. Consider using each of the four top left cells in Table 1 in turn, to estimate four odds ratios relative to the baseline (bottom right) cell:
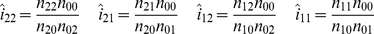
In Text S3 we show that, under a rare disease assumption, these estimated odds ratios 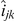 can be considered as estimates of the following functions of 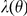, where 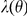 refers to to the log odds ratio estimated in the method of Wu et al. [1]:
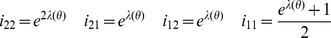
(16)


To construct our proposed tests, we therefore propose to use the four relationships in (16) as four estimating equations for 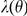, and test the hypothesis that 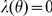 (case/only test) or that 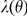 is equal for cases and controls (case/control test). Further motivation for our tests is provided in Text S3. Note that 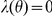 corresponds to the situation where all four of the ‘interaction’ odds ratios (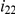, 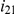, 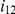, 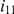) equal 1.

We construct two separate estimates of 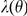, using the data in Table 1 as tabulated for either cases or controls. Equation (16) implies that we can estimate 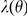 via a weighted average:
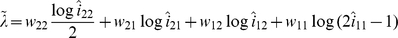
where 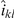 relates to the estimate of 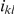 obtained from Table 1, and the weights 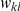 are chosen to sum to 1 and make the variance of 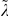 minimum (see Text S1 for details). Having estimated 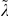 and its variance 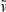 (see Text S1) separately using data from either cases or controls, we can then construct ‘joint effects’ tests:
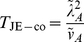
(17)

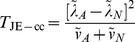
(18)where again 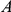 and 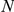 refer to the quantities calculated within cases and controls respectively.

A difficulty with estimation arises when 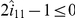. If this occurs, we replace the objective quantity 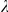 by
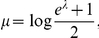
which reduces to zero if 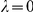 (i.e. possesses the same desirable property under the null hypothesis). Writing 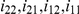 in terms of 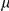, we obtain four estimating equations for 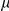 instead of 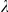, and we estimate 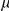 as:
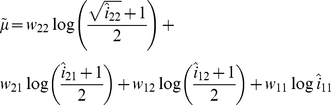
with optimal weights chosen to make the variance minimum as before. Estimating the variance of 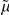 as 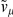, this results in alternate versions of our joint effects tests:
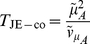
(19)

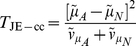
(20)where again 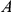 and 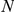 refer to quantities calculated within cases and controls respectively.

### Relationship to standard regression approaches

Text S3 motivates our ‘joint effects’ tests through consideration of the relationship between the original Wu et al. [1] method and standard logistic regression. A natural question of interest is the relationship between the other two methods described here (FE, WZ) and standard regression approaches – and, in particular, to what extent the different odds ratios (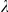) estimated by these methods correspond to the usual interaction parameters (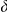 and 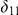, 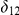, 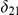, 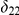) in Equations (1) and (2). In Text S4 we show, for each of these methods, the relationship between the parameters estimated in that method and those estimated in standard logistic or linear regression. In addition, in Text S5, we show that the WZ case-only statistic can be viewed equivalently as a score test with respect to the interaction parameter 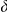. It would be of interest to determine whether a similar relationship holds for the other statistics considered here. However, providing this derivation for the remaining statistics is beyond the scope of the current manuscript, and we defer it to future work.

### Simulation study

We performed computer simulations to evaluate the performance (type 1 error and power) of the various test statistics described above. For the Wu and adjusted Wu methods, haplotype frequencies in cases and controls were calculated from unphased genotype data using an EM algorithm as implemented in either PLINK or the R library ‘Genetics’. The general structure of the disease models we considered is shown in Table 3, assuming two loci G and H, each having two alleles 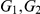 and 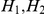. We simulated 1000 cases and 1000 controls from a general population assumed to be in HWE. Writing the haplotype frequencies in the general population as 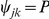 (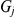
−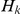) for 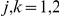, we considered the same two sets of haplotype frequencies considered by [1]:

1. Loci not in LD: 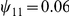, 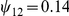, 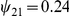 and 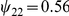.
2. Loci in LD: 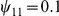, 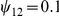, 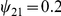 and 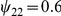.

**Table 3:** Structure of log odds 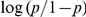 in disease models used for simulation study.

Model	Locus G	Locus H		
		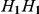	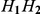	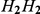
Recessive 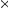 Recessive	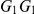	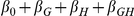	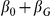	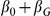
	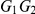	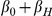	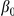	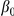
	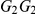	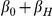	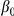	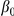
Dominant 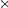 Dominant	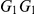	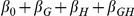	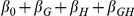	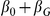
	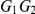	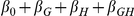	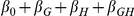	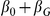
	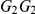	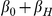	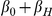	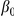
Additive 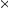 Additive	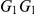	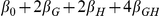	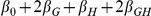	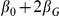
	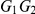	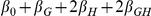	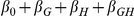	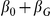
	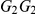	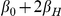	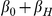	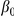
Dominant 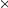 Additive	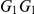	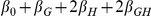	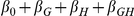	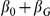
	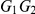	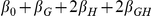	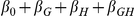	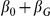
	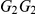	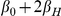	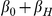	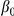

When the two SNPs were not in LD, we examined the performance of both case/control and case-only statistics. When the SNPs were simulated to be in LD, we examined only the performance of case/control statistics (since we know that case-only statistics will show inflated type 1 error in this situation). To investigate type 1 error we considered 8 scenarios, each using 10,000 data replications. To investigate power we considered a further 4 scenarios, each using 1,000 data replications. The structure of the simulated models and the parameter values assumed are given in Tables 3 and 4. Note that in Tables 3 and 4 we denote the baseline, main effect and interaction parameter values (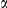, 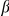, 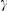, 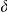 in Equation (1)) as (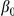, 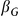, 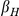, 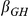) respectively. In each scenario apart from 5c and 5d, the baseline regression coefficient 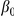 was chosen to equal 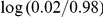, corresponding to a baseline penetrance of 2%. For Scenarios 5c and 5d we assumed a rarer disease, with baseline penetrance 0.0001. For each power scenario, we increased 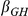 from 0 (no interaction) to a value at which the power to detect an effect (at significance level 0.01) was close to 100% for the best-performing statistics.

**Table 4:** Description of simulation scenarios.

Scenario^a^	Description
1	Both loci have no effect, corresponding to 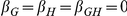 in a Recessive 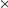 Recessive model
2	Locus G has main effect in a Recessive 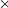 Recessive model, with 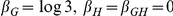
3	Locus G has main effect in a Dominant 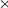 Dominant model, with 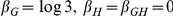
4	Locus G has main effect in an Additive 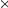 Additive model, with 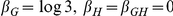
5a	Both loci have main effects in an Additive 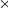 Additive model, with 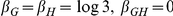
5b	Both loci have main effects in a Recessive 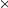 Recessive model, with 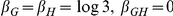
5c	As for Scenario 5a, but assuming a rare disease (baseline penetrance 0.0001)
5d	As for Scenario 5b, but assuming a rare disease (baseline penetrance 0.0001)
6^b^	Recessive 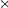 Recessive with either no main effects ( 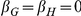 ) or main effect at locus G ( 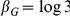 )
7^b^	Dominant 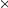 Dominant with with either no main effects ( 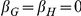 ) or main effect at locus G ( 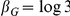 )
8*^b^*	Additive 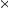 Additive with with either no main effects ( 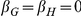 ) or main effect at locus G ( 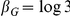 )
9^b^	Dominant 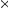 Additive with with either no main effects ( 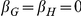 ) or main effect at locus G ( 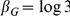 )

a In each scenario (apart from 5c and 5d) the baseline regression coefficient 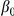 was chosen to equal 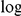
(0.02/0.98), corresponding to a baseline penetrance of 2%.

b For Scenarios 6–9 we increased 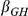 from 0 (no interaction) to a value at which the power to detect an effect (at significance level 0.01) was close to 100% for the best-performing statistics.

In addition to the test statistics described above, when comparing power (Scenarios 6–9) we also calculated several additional statistics. Firstly, as an ‘optimal’ test we considered analysing the data assuming the ‘correct’ model (i.e. imposing the correct structure in terms of whether a model was assumed to be additive, dominant or recessive at each locus, see Table 4). For case/control data this was achieved by using logistic regression with the correct coding of predictor variables at each locus, and then comparing models in which an interaction term was or was not included via a likelihood ratio test. For case-only data, the ‘optimal’ analysis was implemented by using the Wellek and Ziegler statistic (14) with the correct coding of predictor variables (corresponding to an additive, dominant or recessive model) at each locus. For comparison, we also considered ‘sub-optimal’ tests where an incorrect coding for the simulation model was used. Secondly, we considered an ‘ideal’ version of the Wu et al. statistics (Equations (6) and (7)), in which we assumed haplotypes could be inferred without error. In this case, the formulae proposed by Wu et al. [1] should be correct, as there is no increase in the asymptotic variances used in the denominator due to phase uncertainty. Although not achievable in practice, for theoretical interest we investigated the performance of the Wu et al. statistics (with respect to both type 1 error and power) in this ‘ideal’ situation.

To gain additional insight into the properties of the methods considered, for Scenario 7 we noted the ‘haplotype’ frequencies and resulting LD measures 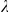, 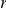 and 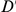 obtained from the EM algorithm applied (separately) to cases and controls (as used in the Wu and adjusted Wu approaches). These were compared to the true haplotype frequencies and correlation measures (as implied by the generating model), the genotype-based correlation coefficient (as used in the Wellek and Ziegler inspired approaches), and the haplotype frequencies and correlation measures calculated from Table 2 (which are, effectively, those used by PLINK).

### Data application

As an illustration of the methods described, we also applied them to real data from a publicly available genome-wide data set consisting of 1748 cases of Crohn's disease and 2938 population-based controls obtained from the Wellcome Trust Case Control Consortium (WTCCC) [2]. Since this exercise was purely for illustrative purposes, in the interests of time we limited our analysis to that of a single chromosome, chromosome 22. We used the same quality control procedures as the WTCCC [2] to remove poor-quality SNPs and samples prior to analysis. This generated 5750 SNPs across chromosome 22, resulting in 16,528,375 pairwise combinations to be tested for interaction.

## Results

### Evaluation of type 1 error

**Figure 1:**
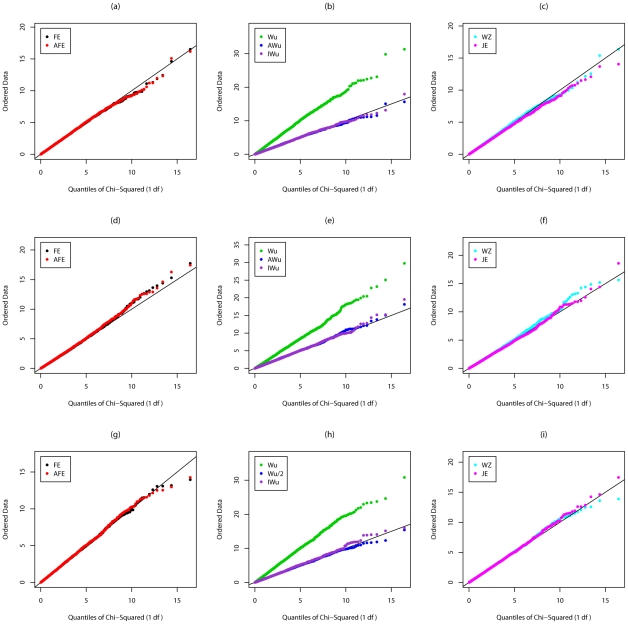
Chi-squared (1 df) Q-Q plot for Scenario 1 (Global Null). Top panels ((a), (b) and (c)): Case/Control not in LD; Middle panels ((d), (e) and (f)): Case/Control in LD; Bottom panels ((g), (h) and (i)): Case-Only not in LD; FE: Fast-Epistasis; AFE: Adjusted FE; Wu: Wu et al. statistic; AWu: Adjusted Wu statistic; IWu: Ideal Wu statistic; WZ: Wellek and Ziegler statistic; JE: Joint Effects statistic.

Figure 1 shows quantile-quantile (QQ) plots of the distribution of the different test statististics calculated in Scenario 1 (so under the global null of no effects at either locus). For a test that is performing correctly (i.e. with well-calibrated type 1 error), we would expect to see all points lying on the line with slope equal to 1. We find this to be true for all methods *except* the original Wu et al. [1] statistics (Equations (6) and (7)), which show strong departure from the line, indicating a severe inflation in type 1 error.

**Figure 2:**
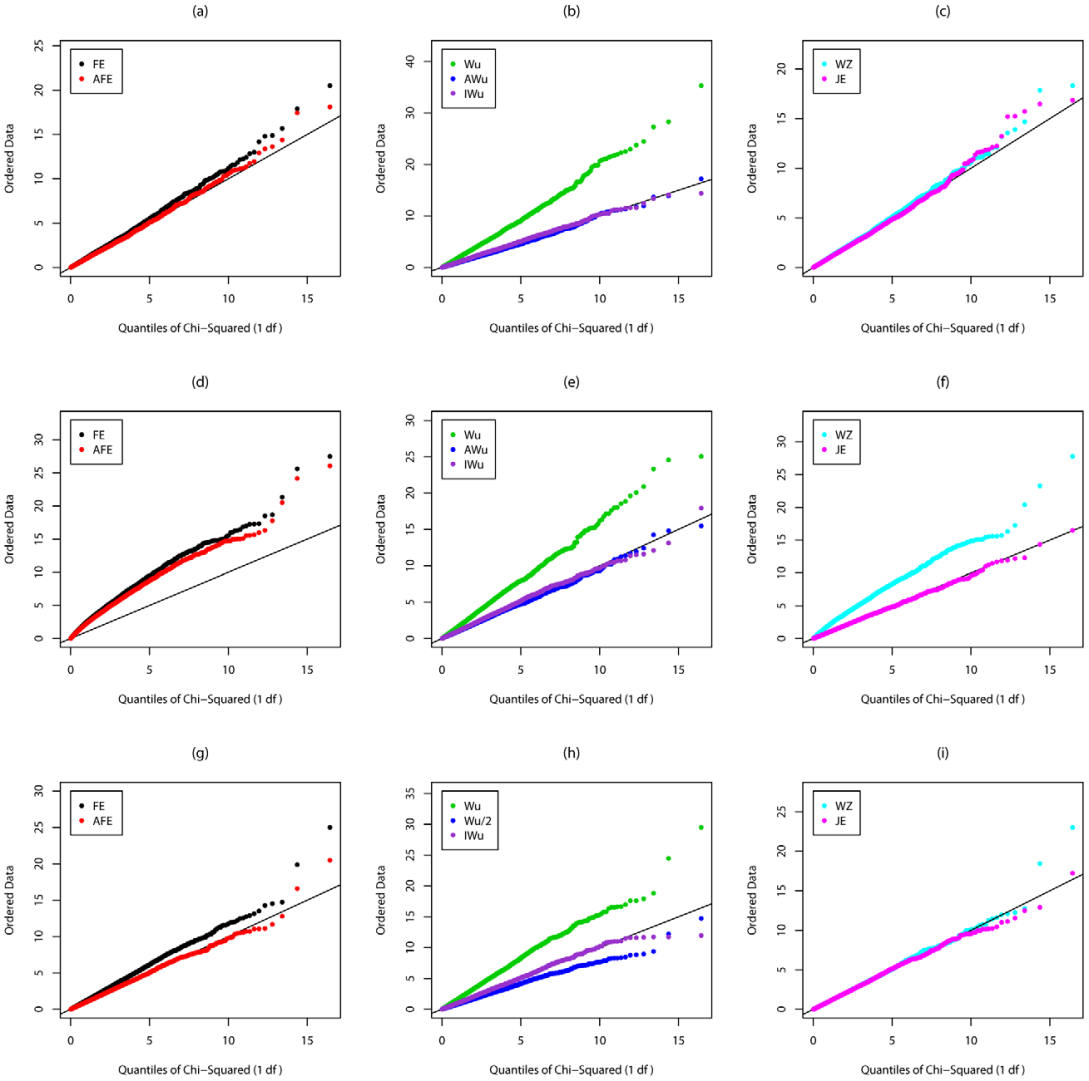
Chi-squared (1 df) Q-Q plot for Scenario 2 (Recessive effect at locus G). Top panels ((a), (b) and (c)): Case/Control not in LD; Middle panels ((d), (e) and (f)): Case/Control in LD; Bottom panels ((g), (h) and (i)): Case-Only not in LD; FE: Fast-Epistasis; AFE: Adjusted FE; Wu: Wu et al. statistic; AWu: Adjusted Wu statistic; IWu: Ideal Wu statistic; WZ: Wellek and Ziegler statistic; JE: Joint Effects statistic.

Figure 2 shows QQ plots for Scenario 2 in which locus G has a recessive main effect. Again the original Wu et al. [1] statistics show a severe inflation in type 1 error. A severe inflation is also seen for the Wellek and Ziegler inspired statistics and PLINK's *fast-epistasis* tests (both the original and our adjusted version) in case/control analysis, when the two SNPs considered are in LD in the general population. (Some theoretical explanation for these results can be found in Text S2). This inflation in the presence of LD is not seen for the ideal Wu statistics or for our new joint effects statistics. For case-only analysis, we see a small inflation in type 1 error for PLINK's *fast-epistasis* test, which is corrected through use of our adjusted version of this test. We also see a slight deflation in type 1 error (indicating the method is conservative) for our adjusted Wu statistic.

A similar pattern is seen for Scenario 3 (in which locus G has a dominant main effect, see Figure S1) except that, in this case, the Wellek and Ziegler inspired case/control statistic does not appear to show inflated type 1 error in the presence of LD, and, for case-only analysis, PLINK's *fast-epistasis* test shows a slight deflation (rather than inflation) in type 1 error, while our adjusted Wu statistic shows a slight inflation. Correct type 1 errors are achieved by the ideal Wu statistics and by our new joint effects statistics. Results from Scenario 4 (in which locus G has an additive main effect) are shown in Figure S2. In this case, all methods appear to have correct type 1 error except the original Wu et al. [1] statistics and the Wellek and Ziegler inspired case/control statistic in the presence of LD.

**Figure 3:**
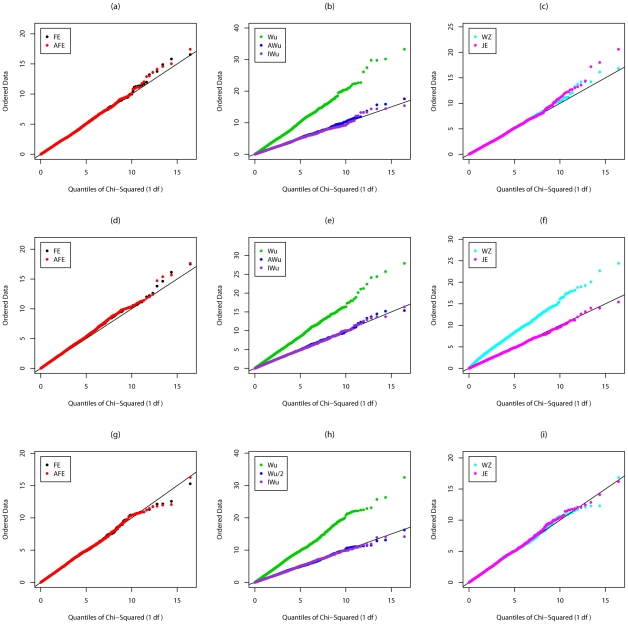
Chi-squared (1 df) Q-Q plot for Scenario 5c (Rare disease, Additive effects at both loci). Top panels ((a), (b) and (c)): Case/Control not in LD; Middle panels ((d), (e) and (f)): Case/Control in LD; Bottom panels ((g), (h) and (i)): Case-Only not in LD; FE: Fast-Epistasis; AFE: Adjusted FE; Wu: Wu et al. statistic; AWu: Adjusted Wu statistic; IWu: Ideal Wu statistic; WZ: Wellek and Ziegler statistic; JE: Joint Effects statistic.

**Figure 4:**
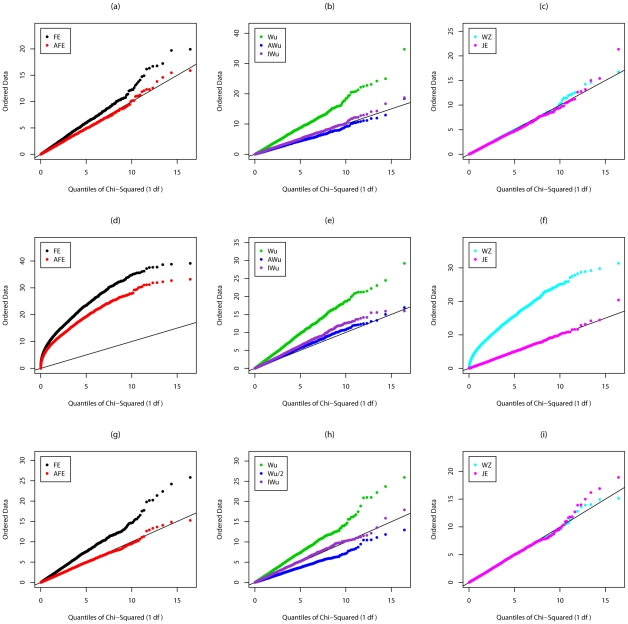
Chi-squared (1 df) Q-Q plot for Scenario 5d (Rare disease, Recessive effects at both loci). Top panels ((a), (b) and (c)): Case/Control not in LD; Middle panels ((d), (e) and (f)): Case/Control in LD; Bottom panels ((g), (h) and (i)): Case-Only not in LD; FE: Fast-Epistasis; AFE: Adjusted FE; Wu: Wu et al. statistic; AWu: Adjusted Wu statistic; IWu: Ideal Wu statistic; WZ: Wellek and Ziegler statistic; JE: Joint Effects statistic.

Figures S3, Figure S4, Figure 3, and Figure 4 show the results from Scenarios 5a, 5b, 5c, 5d, in which both loci have main effects. Provided the disease is rare (Figure 3 and Figure 4), our joint effects statistics show correct type 1 error, while the adjusted *fast-epistasis* and Wellek and Ziegler methods can show inflated type 1 errors, particularly in the presence of LD. (Some theoretical explanation for these results can be found in Text S2). The Adjusted Wu method has generally correct type 1 error although it appears to be slightly conservative for case/only analysis in Figure 4. When the disease is more common (Figures S3 and S4), the presence of main effects appears to have an impact on the type 1 error of virtually all methods, indicating that none are completely immune from detecting pairs of loci that are both involved in disease, but which do not, in fact, require any statistical interaction term to describe their action. The only method that appears immune to this problem is the ideal Wu statistic applied to case/control (but not to case-only) data.

### Evaluation of power

**Figure 5:**
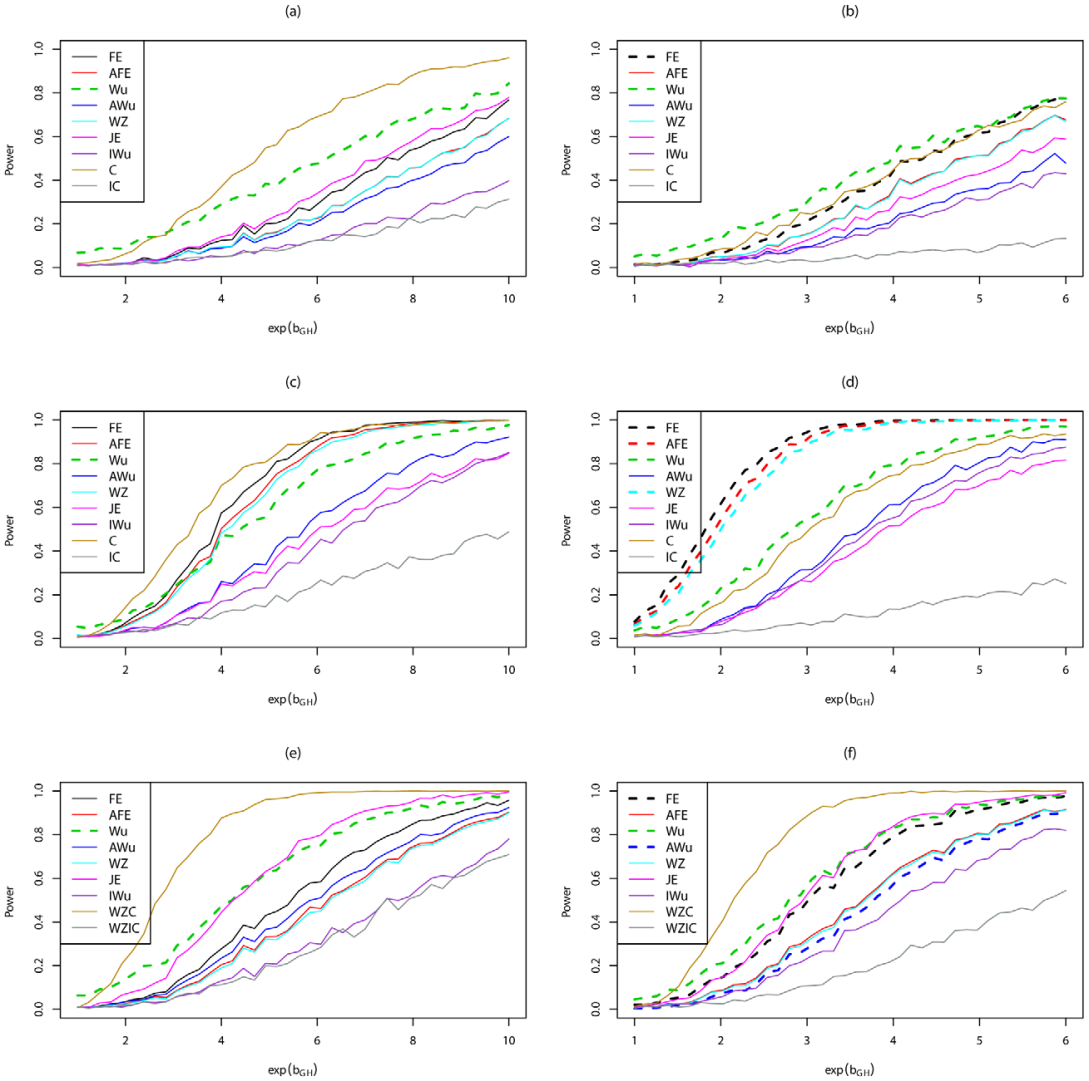
Power curves for Scenario 6 (Recessive 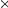 Recessive). Power to achieve significance level 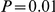. Top panels ((a) and (b)): Case/Control not in LD; Middle panels ((c) and (d)): Case/Control in LD; Bottom panels ((e) and (f)): Case-Only not in LD; Left hand panels ((a), (c) and (e)): No main effect; Right hand panels ((b), (d) and (f)): Locus G has main effect; FE: Fast-Epistasis; AFE: Adjusted FE; Wu: Wu et al. statistic; AWu: Adjusted Wu statistic; WZ: Wellek and Ziegler statistic; JE: Joint Effects statistic; IWu: Ideal Wu statistic; C: Logistic regression using correct coding; IC: Logistic regression using incorrect ( = Recessive
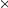
Dominant) coding; WZC: Wellek and Ziegler case-only statistic using correct coding; WZIC: Wellek and Ziegler case-only statistic using incorrect ( = Recessive
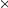
Dominant) coding.

Figure 5 shows power curves for Scenario 6 (Recessive
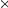
Recesssive model) for all methods considered, including methods that assume ‘correct’ or ‘incorrect’ knowledge of the true structure of the underlying generating model. The left hand panels show results when there are no main effects, while the right hand panels show results in the presence of a main effect at locus G. We use solid lines to represent methods that have been shown (Figure 1, Figure 2, Figure 3, Figure 4; Figures S1, S2, S3, S4) or would be expected on theoretical grounds to have correct type 1 error. We use dashed lines to represent methods that have been shown (Figure 1, Figure 2, Figure 3, Figure 4; Figures S1, S2, S3, S4) to have incorrect type 1 error under the relevant generating model (and whose ‘power’ should therefore be interpreted cautiously in the light of that fact). In all cases, we find that the highest power among methods that correctly control the type 1 error is seen for ‘optimal’ tests that impose the correct structure, while the lowest power is seen for ‘sub-optimal’ tests that impose the incorrect structure, as might be expected from standard statistical theory. Amongst the other tests, no method consistently outperforms the others; in some cases our joint effects test has highest power, in other cases the adjusted Wu or adjusted or original *fast-epistasis* tests perform best. The ideal Wu test (in which we assume haplotypes can be estimated without uncertainty) shows generally lower power than the other tests considered, in this scenario.

**Figure 6:**
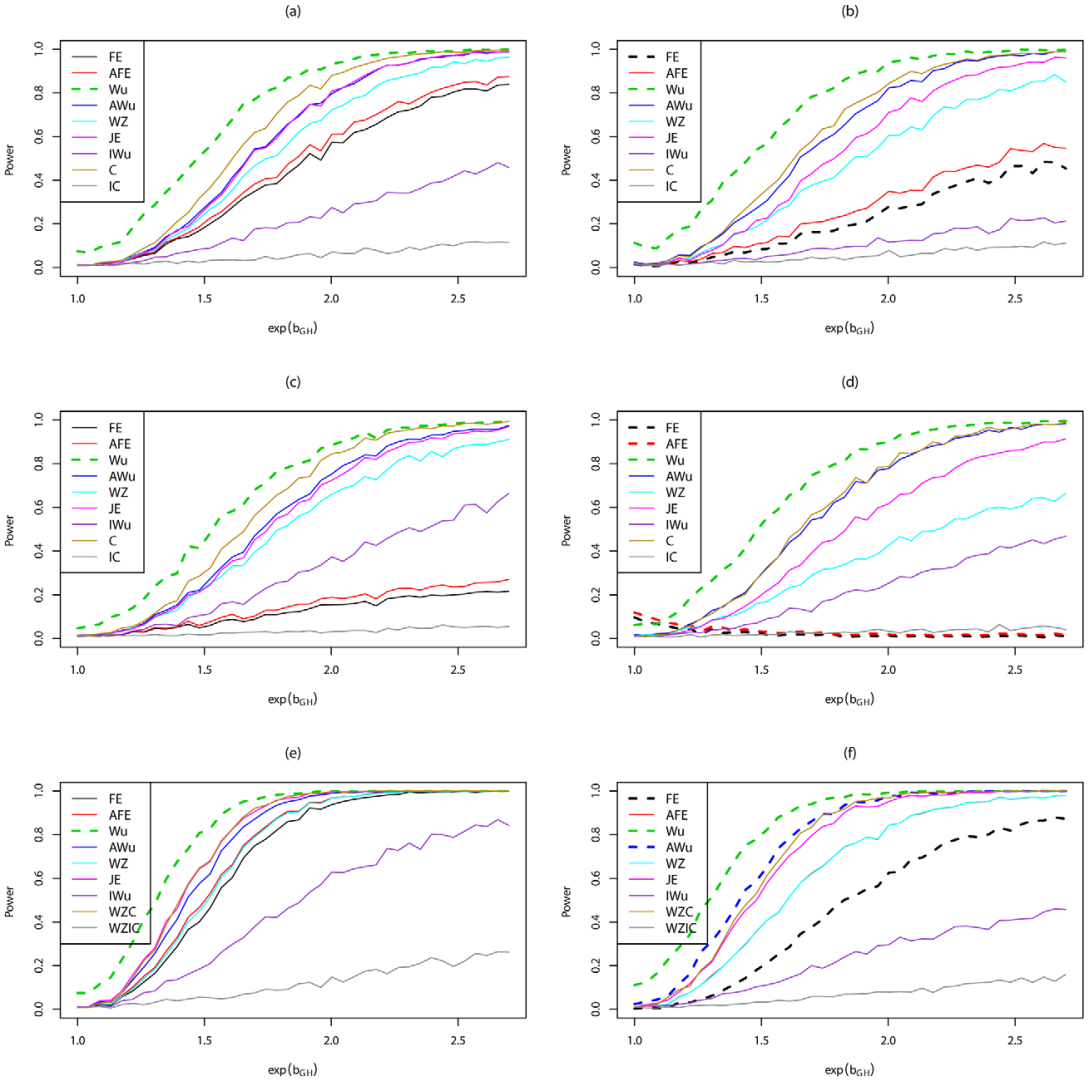
Power curves for Scenario 7 (Dominant 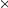 Dominant). Power to achieve significance level 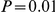. Top panels ((a) and (b)): Case/Control not in LD; Middle panels ((c) and (d)): Case/Control in LD; Bottom panels ((e) and (f)): Case-Only not in LD; Left hand panels ((a), (c) and (e)): No main effect; Right hand panels ((b), (d) and (f)): Locus G has main effect; FE: Fast-Epistasis; AFE: Adjusted FE; Wu: Wu et al. statistic; AWu: Adjusted Wu statistic; WZ: Wellek and Ziegler statistic; JE: Joint Effects statistic; IWu: Ideal Wu statistic; C: Logistic regression using correct coding; IC: Logistic regression using incorrect ( = Dominant
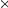
Recessive) coding; WZC: Wellek and Ziegler case-only statistic using correct coding; WZIC: Wellek and Ziegler case-only statistic using incorrect ( = Dominant
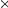
Recessive) coding.

Figure 6 shows power curves for Scenario 7 (Dominant
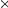
Dominant model). The original Wu statistic shows apparent highest power, but this observation is tempered by the fact that we know it has inflated type 1 error. Again, highest power among methods that correctly control the type 1 error is generally obtained for ‘optimal’ tests that impose the correct structure, although in some cases this power is closely matched by the adjusted Wu or joint effects tests. The original and adjusted *fast-epistasis* tests show low power when applied to case/control data. The ideal Wu test also shows generally low power when applied to either case/control or case-only data.

Figure S5 shows power curves for Scenario 8 (Additive
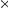
Additive model). Most methods perform fairly similarly, except for analysis under an incorrect model and the ideal Wu test, which both show lower power. For case/control data, in this scenario, the Wellek and Ziegler test slightly outperforms most other tests.

Figure S6 shows power curves for Scenario 9 (Dominant
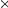
Additive model). Again we find that the highest power among methods that correctly control the type 1 error is seen for ‘optimal’ tests that impose the correct structure, while the lowest power is seen for either for the ideal Wu statistic, or for ‘sub-optimal’ tests that impose the incorrect structure. Amongst the other tests, no method consistently outperforms the others; in some cases the Wellek and Ziegler test shows highest power, whereas in other cases the joint effects or adjusted Wu statistics show highest power.

Table 5 shows the true and estimated haplotype frequencies and correlation measures, as used by several different methods, under one particular setting for simulation Scenario 7. When data is simulated without LD between the loci, we see that, in controls, both the EM algorithm (as used in the Wu et al. and adjusted Wu methods) and the allele counting algorithm (used in PLINK's *fast-epistasis* method) give very similar results with respect to estimated haplotype frequencies and resulting correlation measures. The correlation measures (along with the Wellek and Ziegler genotype-based correlation coefficient) are correctly estimated as being close to 0. The slight departure from 0 results from the fact that the disease is not particularly rare, and so the presence of an interaction effect will cause unaffected controls, as well as cases, to show some slight correlation between alleles at the two loci.

**Table 5:** True and estimated haplotype frequencies and correlation measures used by different methods.

LD	Quantity	Cases				Controls			
		True	Wu	FE	WZ	True	Wu	FE	WZ
No	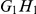	0.091	0.107	0.092	-	0.059	0.059	0.059	-
	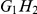	0.150	0.136	0.151	-	0.140	0.140	0.140	-
	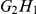	0.243	0.227	0.242	-	0.240	0.241	0.240	-
	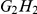	0.516	0.531	0.515	-	0.561	0.560	0.561	-
	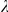	0.253	0.609	0.257	-	−0.015	−0.025	−0.012	-
	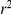	0.003	0.017	0.003	0.013	2.3×10^−6^	0.001	2.3×10^−4^	0.001
	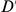	0.066	0.160	-	-	0.005	0.059	-	-
Yes	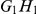	0.148	0.158	0.118	-	0.099	0.099	0.079	-
	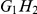	0.104	0.095	0.134	-	0.100	0.100	0.120	-
	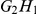	0.196	0.187	0.223	-	0.200	0.200	0.220	-
	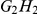	0.551	0.561	0.522	-	0.600	0.601	0.581	-
	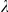	1.386	1.615	0.711	-	1.089	1.086	0.559	-
	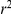	0.088	0.119	0.023	0.104	0.047	0.047	0.012	0.048
	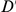	0.369	0.429	-	-	0.283	0.282	-	-

Data was simulated under Scenario 7 (Dominant
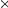
Dominant) with 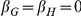 and 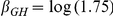. The table shows the mean of the relevant quantity (haplotype frequency or correlation measure) as estimated within cases or controls from 1000 simulation replicates. Wu: Estimated using EM algorithm as used by Wu et al. [1] methods. FE: Estimated based on counts in Table 2, as used by *fast-epistasis* methods. WZ: estimated using genotype-based correlation coefficient, as used by Wellek and Zigler inspired methods.

In cases (with no LD) however, the story is very different. All methods show correlation between alleles at the two loci, however the haplotype frequencies and resulting correlation measures estimated using PLINK's allele counting algorithm seem to be much closer to the true generating values. The EM algorithm (as used in the Wu et al. and adjusted Wu methods) produces upwardly biased estimates, presumably because of the incorrect (within cases) HWE assumption made. This results in much higher apparent correlation, which could plausibly increase power when testing whether correlation between alleles two loci exists (case-only test) or is different between cases and controls (case/control test)). However, the power of any given test will depend not just on the level of apparent correlation, but also on the estimated variance of the correlation measure used, and our results overall suggest that the bias induced by the incorrect HWE asssumption does not necessarily always translate to a substantially improved power.

In the presence of LD, for controls the EM algorithm (as used in the Wu et al. and adjusted Wu methods) appears to better capture the true haplotype frequencies and resulting correlation measures, while the PLINK's allele counting algorithm produces results that are biased downwards (i.e. towards showing lower levels of correlation). For cases, PLINK's allele counting algorithm produces correlation measures that are biased downwards from the true values, while the EM algorithm produces correlation measures that biased upwards. Given that any analysis in the presence of LD needs to be based on the difference in correlations between cases and controls, it is unclear to what extent these biases will operate to improve power for one method over another, although the results shown in Figure 6 suggest that these bias may partly account for the high power of the adjusted Wu methods in that scenario.

### Data application

Figure S7 shows the results from applying the different methods to 5750 SNPs across chromosome 22 genotyped in the WTCCC Crohn's disease dataset. Since SNPs on the same chromosome are likely to be in LD, we limited our analysis to the case/control version of all statistics considered. Given the large number of potential tests performed (16,528,375 pairwise combinations), for the joint effects, *fast-epistasis* and Wellek and Ziegler inspired methods, we only output results passing a 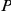 value threshold of 0.001 (although note that, for the *fast-epistasis* statistic, PLINK in fact only performed a total of 13,818,410 tests that passed its validity criteria).

The QQ plots (Figure S7) show that the joint effects, *fast-epistasis* and Wellek and Ziegler inspired statistics all follow the expected distribution under the null hypothesis, even in this tail (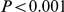) of the distribution. We also noted that, for these three methods, the proportion of tests falling into this tail was 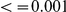, as expected (data not shown). The most computationally efficient implementation was PLINK, which took approximately 20 minutes to perform 13,818,410 tests. The Wellek and Ziegler and joint effects methods were considerably slower, each taking 20 hours (on the same computer system) to perform 16,528,375 tests. We implemented the Wellek and Ziegler and joint effects statistics through code written by ourselves in R, and so these times could be considerably reduced by re-writing the code (e.g. in C++) and making use of mechanisms for efficient binary data storage.

The original and adjusted Wu methods were prohibitively slow to calculate for all 16,528,375 pairwise combinations, most likely because of the requirement of these methods to estimate haplotype frequencies from unphased genotype data (e.g. via an EM algorithm). (We implemented these methods through code written by ourselves in R; calculation might be achievable in reasonable time through use of more efficient programming in C++, binary data storage and parallel execution on a computer cluster). Figure S7 therefore shows the results for the original and adjusted Wu methods for a subset of 10813 SNP pairs consisting of the first and the thousandth SNP, each paired with all others. Even in this reduced data set, we can see that the adjusted Wu statistic follows the expected distribution under the null hypothesis while the original Wu statistic shows an inflated distribution, in line with the results we found in our computer simulations.

The results in Figure S7 do not provide any strong evidence for the existence of interactions between SNPs on chromosome 22 in the WTCCC Crohn's data. However, it is of interest to see to what extent the different methods implicate the same ‘top SNP pairs’. Figure S8 plots the observed test statistics for the joint effects, *fast-epistasis* and Wellek and Ziegler inspired statistics against one another. The results from these three methods are seen to be broadly correlated, with the same SNP pairs tending to fall at the extreme of the distribution, regardless of which method is used.

Since we were unable to calculate the Wu and adjusted Wu statistics for all pairs of SNPs, at the suggestion of a reviewer, we used another approach for calculating these statistics, which we hoped would be computationally quicker. We used a phasing algorithm to infer haplotypes across chromosome 22, for each individual. We carried out this step using the program SHAPEIT [35], which has the advantage of outputting for each individual not just a single “most likely” haplotype configuration, but additionally allows one to store the uncertainty and sample a set of haplotype configurations. We sampled 100 replicate haplotype configurations for each individual. Since the idea of the Wu method is to compare ‘apparent LD’ within cases to that within controls, we initially carried out the phasing in case and control groups separately, although we later compared our results to those obtained when phasing the cases and controls together.

Having generated 100 replicates of phased haplotypes, we then calculated, for each pair of SNPs, the mean (over the 100 replicates) haplotype frequencies in cases and controls. (The haplotype frequencies within each replicate were calculated simply by counting resolved case and control haplotypes). We used these mean haplotype frequencies in the formulae for the Wu and adjusted Wu statistics (Equations 7 and 9 respectively). Note that these formulae were derived on the basis of sampling theory under the assumption of a certain number of observed haplotypes, and it is unclear whether the same theoretical arguments should apply to haplotype frequencies that have been estimated in a different way. In particular, SHAPEIT uses a hidden Markov model that is motivated by population genetics principles, resulting in a greater borrowing of information across SNPs and individuals than is used in the other approaches. This fact, together with the fact we averaged (over 100 replicates), suggests that the haplotype frequencies (and thus 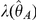 and 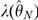) estimated from SHAPEIT may be more accurate and less variable than those estimated in the other approaches, thus requiring a smaller variance in the denominator of the test statistic. To address this issue, we used an additional strategy of calculating the variance directly from the 100 replicates. Within each replicate, we calculated the haplotype frequencies and log odds ratios 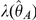 and 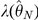. We then calculated the sample mean and variance of 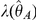 and 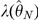 over the 100 replicates and constructed a ‘SHAPEIT variance-based Wu (SVBW) test statistic’:
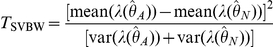



Figure S9 shows QQ plots for the Wu and adjusted Wu test statistics (Equations 7 and 9) applied to the mean estimated haplotype frequencies from SHAPEIT, for the subset of 10813 SNP pairs consisting of the first and the thousandth SNP, each paired with all others. The test statistics (shown in red and black) are seen to be considerably deflated in comparison to the expected 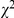 distribution, suggesting that the variance of the SHAPEIT-derived haplotype frequencies is indeed considerably lower than that implied by Equations 7 and 9. We noticed, however, that the 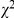 test statistics appeared to be approximately half their expected value. We therefore constructed an alternative ‘SHAPEIT mean-based Wu (SMBW) test statistic’:
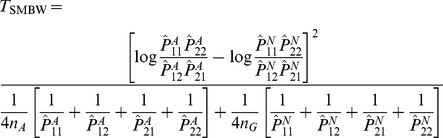
(21)which can be seen to be equivalent to the original Wu case/control statistic, but under the assumption of double the number of haplotypes. The SMBW test statistics (shown in green) are seen to closely follow the expected 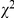 distribution, suggesting that variance of the SHAPEIT-derived haplotype frequencies is indeed equivalent to what would be obtained from observing twice the number of haplotypes. We consulted the description of the algorithm used by SHAPEIT [35] and noticed that it involves an iterative procedure of updating an individual's current haplotype configuration by sampling haplotypes from a set of currently resolved haplotypes (for the other individuals in the data set), in such a way that recombination and mutation events are allowed for. This means that, for SNPs close together, the sampling procedure would effectively be sampling alleles from 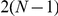 currently-resolved haplotypes (where 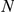 is the number of individuals in the data set) while for SNPs that are far apart, a recombination event is virtually guarranteed and so the sampling procedure is effectively sampling from 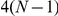 haplotypes constructed by sampling the alleles at each SNP independently. Since the majority of our pairwise tests involve SNPs that are far apart, the majority of the tests will indeed closely correspond to effectively observing 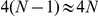 haplotypes. Note that this argument is quite similar to the argument that could be used to justify the construction of PLINK's *fast-epistasis* statistic on the basis of 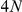 alleles.

Logically, one would expect that the variance could be estimated even better by allowing for the actual recombination distance between each pair of SNPs, so that SNPs that are closer together are considered to have a probability 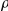 of undergoing a recombination and thus being sampled from 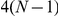 haplotypes, and probability 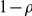 of not undergoing a recombination and thus being sampled from 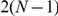 haplotypes. (For definition of 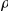, see [35]). However, we found implementation of this approach resulted in test statistics that did not follow the expected 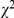 (on 1 df) distribution quite as well as simply assuming 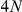 or 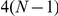 haplotypes (data not shown). One possible explanation is that the iterative nature of the SHAPEIT algorithm means that even SNPs that lie close together are likely to be subject to a recombination event at some point during the procedure, generating closer to 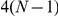 effective haplotypes. Further work, beyond the scope of this paper, would be required to follow up the explanation for these observations in more detail.

Figure S10 (Panel (a)) shows the QQ plot for the SHAPEIT variance-based Wu (SVBW) test statistic, for the subset of 10813 SNP pairs consisting of the first and the thousandth SNP, each paired with all others. Although the majority of the points do lie on the expected line, there are a number of outliers. We noticed that the most severe outliers corresponded to pairs of SNPs that lie within 1 cM of one another (shown in red), suggesting that the variance of the haplotype frequencies within short regions may perhaps be under-estimated by the SHAPEIT algorithm. (Another explanation is that these are true interactions and/or haplotype effects, however this seems a little unlikely given that they are not identified by any other method). We removed all pairs of SNPs that lie within 1 cM of one another from both the SVBW and SMBW results, which resulted in test statistics that followed the expected distribution more closely (Panels (b) and (c)). Panel (d) shows a comparison between the resulting SMBW and SVBW test statistics, showing how extremely similar they are. Figure S11 Panels (a) and (b) show a comparison between the SMBW and SVBW statistics and the AWu statistic, while Panels (c) and (d) show a comparison between the SMBW and SVBW statistics and the JE statistic. Although these different test statistics are by no means identical, they are seen to be broadly correlated, as expected.

Figure S12 shows a comparison of the SMBW (left hand panels) and SVBW (right hand panels) results from haplotypes estimated by applying SHAPEIT to cases and controls separately (y axes) or together (x axes). Points marked in red on the top panels correspond to SNP pairs where the SNPs are less than 1 cM apart; these pairs are seen to generate outliers for the SVBW test regardless of whether SHAPEIT is applied to cases and controls separately or together. These outliers do not occur with the SMBW test when SHAPEIT is applied to cases and controls together. The bottom panels repeat these plots, but with SNP pairs where the SNPs are less than 1 cM apart removed. Overall the results from applying SHAPEIT to cases and controls separately (y axes) or together (x axes) are seen to be highly correlated, particularly for the SMBW test. We investigated the outliers (where the results were very different according to whether cases and controls were phased separately or together) and noticed that the vast majority of these corresponded to SNPs whose minor allele frequency is close to 0.5, and for which there had been a swap with respect to which allele was designated as the minor allele between the case and control groups, when phased separately. This resulted in an incorrect matching of haplotypes between case and control groups, resulting in an incorrect test statistic. (Interestingly, the 7 outliers for which the test statistic is close to 0 when the cases and controls were phased separately are also seen as outliers when compared to the AWu and JE tests (Figure S11), indicating that the results from SHAPEIT applied to cases and controls together are concordant with the AWu and JE results). We found that the allele swap problem had occurred in 46 out of the 5750 SNPs considered i.e. just under 1% of the results presented from applying SHAPEIT to cases and controls separately were incorrect. This might suggest that the strategy of phasing cases and controls together is more reliable, although in practice one could avoid this problem when phasing cases and controls separately by performing a more careful check at the analysis stage. Intuitively, one might expect that the strategy of phasing cases and controls separately might be more powerful when constructing tests that are based on haplotype differences between cases and controls, but a detailed comparison of the relative power of these two approaches would required further investigation.

Although the SHAPEIT approaches appear to result in more accurate haplotype estimation than the EM algorithm-based Wu and AWu approaches, generating haplotype frequency estimates that can (with care) be translated into Wu-like interaction tests, in our hands, implementation of these approaches was not computationally faster than the original Wu and AWu methods. Although generation of 100 replicates of phased chromosome 22 haplotypes in SHAPEIT was relatively fast (taking around 28 hours on our system), our program for generating the resulting SMBW and SVBW test statistics ended up taking about 3 seconds per SNP pair. (For each SNP pair we needed to read in – or store in memory – 100 replicates of phased haplotypes for each individual, in order to pick out the required alleles at the two SNPs, and then calculate haplotype frequencies, 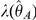 and 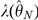, within each replicate, followed by the mean and variance of these quantities across replicates). No doubt more efficient programming, binary data storage and implementation on a computer cluster could considerably speed up this procedure. Given the close correspondence between the SMBW and SVBW tests, together with the better performance of SMBW for SNPs that lie close together, a natural first step might be to initially focus on SMBW alone, for which 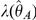 and 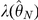 within each replicate, and all variances across replicates, would not need to be calculated.

## Discussion

Here we have investigated, through theoretical derivation, computer simulations and a real data example, the properties of several previously-proposed statistics for performing genome-wide interaction analysis using case/control or case-only data [1], [30], together with a number of alternative statistics proposed by ourselves and others [33], [34]. Our main finding is that the statistics proposed by Wu and colleagues [1] show substantially increased type 1 error due to the incorrect variance estimates used (Equations (6) and (7)) which do not account for the uncertainty induced when estimating phased haplotype frequencies from unphased genotype data. This inflation in type 1 error can be corrected by using a variance estimate that accounts for this uncertainty, as in our adjusted Wu statistics. All other methods investigated appear to show adequate control of type 1 error under the null hypothesis of no genetic effects (main effects or interactions), although several methods (including the *fast-epistasis* method implemented in PLINK [30] and the Wellek and Ziegler method [33], [34]) can show increased type 1 error when there is a main effect at one or both loci, particularly if there is also LD. Only the ideal Wu method and our new joint effects statistics achieve consistent control of type 1 error in the presence of a main effect at just one of the loci.

In terms of power, comparison of the different methods is somewhat complicated by the fact that several of the methods show increased type 1 error in different circumstances. However, even when comparing methods that control the type 1 error rate in a given situation, no method consistently outperforms all others. Generally high power over a range of scenarios is exhibited by the Wellek and Ziegler statistics [33], [34] and by our new joint effects statistics and adjusted Wu statistics. Given that, out of these options, only the joint effects statistics achieve adequate control of type 1 error in the presence of a single main effect, this might suggest that the joint effects tests would be the overall preferred option. Although the ideal Wu method also shows adequate control of type 1 error in the presence of a single main effect, observation of known haplotypes, as required by this method, is unachievable in practice. Even if it were achievable, e.g. through experimental assays that allow determination of haplotypes, or through the use of larger numbers of markers to help infer phase between the two SNPs in question, Figure 5 and Figure 6 and Figures S5 and S6 show that the power achieved by the ideal Wu approach is generally lower than for other approaches. This slightly counter-intuitive result might be due to the fact that the ideal Wu method is not affected by the bias that results from incorrectly assuming HWE when estimating haplotype frequencies in cases, a bias that can potentially increase power.

Somewhat surprisingly, many of our results appear to contradict results presented by Wu and colleagues [1] who found in simulations (using similar generating models to those considered here) and application to real data that their method gave adequate control of type 1 error and higher power than competing methods (including logistic regression analysis under the correct model). We have been unable to fully determine the reason for these discrepencies, even after discussion with the authors of [1], although our discussions have highlighted some possible explanations. With respect to the simulation results, our current understanding is that the simulations performed by Wu et al. did not, in fact, include any consideration of haplotype uncertainty (their simulations simply assumed haplotypes could be observed without error – as, in a simulated data set, they can). This explains the apparently correct type 1 error observed by Wu et al. but it means that all their simulations (of both type 1 error and power) are highly misleading with respect to illustrating how their method might perform in practice (where haplotype uncertainty will invariably exist, particularly at loci that are not in strong LD). It also does not explain the difference in power we see compared to Wu et al. when we also assume haplotypes can be observed without error (our ‘ideal’ Wu statistics). We speculate that one possible explanation for this difference might be that Wu et al. assumed in their simulations that haplotypes come together independently in cases (which is true under a multiplicative haplotype model [28], but not under recessive or dominant models). It is unclear what effect such an erroneous assumption would have on the power of the different methods, but it might possbly explain why Wu et al. found their method to give consistently higher power than logistic regression analysis under a correct model, whereas we find (as might be expected from statistical theory) that logistic regression analysis under a correct model gives generally higher power than the adjusted or ideal Wu statistics.

The explanation of these simulation discrepencies also does not explain why Wu et al. found correct (or possibly slightly deflated) type 1 error in analysis of real data (see QQ plot shown in Figure 1 of Wu et al. (2010) [1]), whereas in our own application of the original Wu et al. (2010) method to real data (Figure S7), we found the same general inflation of test statistics as we observe in computer simulations. One possibility is that Wu et al. inadvertedly divided by a factor of two when using their formulae (our Equations (6) and (7)) to calculate the desired test statistics. This would result in a test that would approximately correspond to our adjusted Wu statistic. In any case, unless or until these issues can be resolved, we recommend use of our new joint effects or adjusted statistics, and urge caution when using Wu et al.'s [1] originally-proposed statistics, on account of the inflated error rate that can result.

We have focussed in this communication on methods that test for interaction *per se* i.e. that test (or attempt to test) the interaction term in a linear model (such as Equation 1). As mentioned previously, if one prefers to test combinations of terms (e.g. in order to implement tests of association allowing for interaction [20], [17]) one may do so by combining a test of the interaction term with some test of the other terms [22]. It is well-known (and indeed can be seen from Figure 5 and Figure 6 and Figures S5 and S6) that case-only tests are more powerful than case/control tests for testing interaction, provided there is no population-level correlation between the two variables being tested. Although such an assumption should in principal be reasonable when testing genetic variants that are located sufficiently far apart as to be expected not to show LD, in practice GWAS data often does display long-range allelic association [17], possibly due to population structure [26] or other confounding influences. This suggests that, in application to GWAS data, the case/control versions of the statistics described here might be preferred over the case-only versions, in spite of their lower power. Alternatively, construction of weighted combinations of the case-only and case/control statistics [36]) might prove a more powerful approach. Several authors have recently proposed the use of retrospective likelihoods [37], [26] that can increase power by exploiting an assumption of gene-gene independence in the underlying population (or in controls, if the disease is rare or controls unselected). These methods have been used, for example, in a conditional search exercise exploiting known loci for prostate cancer in a multi-stage GWAS [38]. The advantage of these frameworks is that they allow the incorporation of covariates (such as principal components scores) to account for population stratification, as well as allowing a wider class of tests. Since the methods described here can all be formulated in terms of (prospective) linear or logistic regression models (see Text S3 and S4), in theory such approaches could be applied to the tests described here. However, an advantage of the current formulations is that closed-form expressions for the tests are available, which makes them attractive when carrying out all pairwise interaction scans in GWAS, on account of the fact that the tests are rapidly computed.

R code for implementing the joint effects, Wellek and Ziegler and adjusted Wu statistics described in this manuscript is available on request from the authors.

## Supporting Information

Figure S1 Chi-squared (1 df) Q-Q plot for Scenario 3 (Dominant effect at locus G). Top panels ((a), (b) and (c)): Case/Control not in LD; Middle panels ((d), (e) and (f)): Case/Control in LD; Bottom panels ((g), (h) and (i)): Case-Only not in LD; FE: Fast-Epistasis; AFE: Adjusted FE; Wu: Wu et al. statistic; AWu: Adjusted Wu statistic; IWu: Ideal Wu statistic; WZ: Wellek and Ziegler statistic; JE: Joint Effects statistic.
(TIF)

Figure S2 Chi-squared (1 df) Q-Q plot for Scenario 4 (Additive effect at locus G). Top panels ((a), (b) and (c)): Case/Control not in LD; Middle panels ((d), (e) and (f)): Case/Control in LD; Bottom panels ((g), (h) and (i)): Case-Only not in LD; FE: Fast-Epistasis; AFE: Adjusted FE; Wu: Wu et al. statistic; AWu: Adjusted Wu statistic; IWu: Ideal Wu statistic; WZ: Wellek and Ziegler statistic; JE: Joint Effects statistic.
(TIF)

Figure S3 Chi-squared (1 df) Q-Q plot for Scenario 5a (Additive effects at both loci). Top panels ((a), (b) and (c)): Case/Control not in LD; Middle panels ((d), (e) and (f)): Case/Control in LD; Bottom panels ((g), (h) and (i)): Case-Only not in LD; FE: Fast-Epistasis; AFE: Adjusted FE; Wu: Wu et al. statistic; AWu: Adjusted Wu statistic; IWu: Ideal Wu statistic; WZ: Wellek and Ziegler statistic; JE: Joint Effects statistic.
(TIF)

Figure S4 Chi-squared (1 df) Q-Q plot for Scenario 5b (Recessive effects at both loci). Top panels ((a), (b) and (c)): Case/Control not in LD; Middle panels ((d), (e) and (f)): Case/Control in LD; Bottom panels ((g), (h) and (i)): Case-Only not in LD; FE: Fast-Epistasis; AFE: Adjusted FE; Wu: Wu et al. statistic; AWu: Adjusted Wu statistic; IWu: Ideal Wu statistic; WZ: Wellek and Ziegler statistic; JE: Joint Effects statistic.
(TIF)

Figure S5Power curves for Scenario 8 (Additive
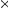
Additive). Power to achieve significance level 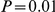. Top panels ((a) and (b)): Case/Control not in LD; Middle panels ((c) and (d)): Case/Control in LD; Bottom panels ((e) and (f)): Case-Only not in LD; Left hand panels ((a), (c) and (e)): No main effect; Right hand panels ((b), (d) and (f)): Locus G has main effect; FE: Fast-Epistasis; AFE: Adjusted FE; Wu: Wu et al. statistic; AWu: Adjusted Wu statistic; WZ: Wellek and Ziegler statistic; JE: Joint Effects statistic; IWu: Ideal Wu statistic; C: Logistic regression using correct coding; IC: Logistic regression using incorrect ( = Additive
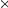
Recessive) coding; WZC: Wellek and Ziegler case-only statistic using correct coding; WZIC: Wellek and Ziegler case-only statistic using incorrect ( = Additive
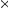
Recessive) coding.(TIF)

Figure S6Power curves for Scenario 9 (Dominant
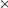
Additive). Power to achieve significance level 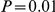. Top panels ((a) and (b)): Case/Control not in LD; Middle panels ((c) and (d)): Case/Control in LD; Bottom panels ((e) and (f)): Case-Only not in LD; Left hand panels ((a), (c) and (e)): No main effect; Right hand panels ((b), (d) and (f)): Locus G has main effect; FE: Fast-Epistasis; AFE: Adjusted FE; Wu: Wu et al. statistic; AWu: Adjusted Wu statistic; WZ: Wellek and Ziegler statistic; JE: Joint Effects statistic; IWu: Ideal Wu statistic; C: Logistic regression using correct coding; IC: Logistic regression using incorrect ( = Dominant
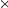
Recessive) coding; WZC: Wellek and Ziegler case-only statistic using correct coding; WZIC: Wellek and Ziegler case-only statistic using incorrect ( = Dominant
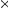
Recessive) coding.(TIF)

Figure S7QQ plots from analysis of pairwise (SNP
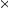
SNP) interactions on chromosome 22 in the WTCCC Crohn's data set. JE: Joint Effects statistic; FE: Fast-Epistasis; WZ: Wellek and Ziegler statistic; Wu: Wu et al. statistic; AWu: Adjusted Wu statistic.(TIF)

Figure S8Correlations between three different methods applied to the WTCCC Crohn's data set. Each point represents a particular SNP
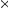
SNP pair on chromosome 22. Shown are pairwise plots of the test statistics generated by each of the three methods. JE: Joint Effects statistic; FE: Fast-Epistasis; WZ: Wellek and Ziegler statistic.(TIF)

Figure S9 QQ plots from analyses based on the mean haplotype frequencies (over 100 replicates) estimated using SHAPEIT. Red crosses denote results calculated using the original Wu formula. Black plusses denote results calculated using the Adjusted Wu formula. Green circles denote results calculated using the SMBW formula, which corresponds to twice the original Wu statistic.
(TIF)

Figure S10 Panel (a): QQ plots from analyses based on the SVBW test statistic. Results from SNP pairs where the SNPs are less than 1 cM apart are shown in red. Panel (b): QQ plots from analyses based on the SVBW test statistic, having removed all SNP pairs where the SNPs are less than 1 cM apart. Panel (c): QQ plots from analyses based on the SMBW test statistic, having removed all SNP pairs where the SNPs are less than 1 cM apart. Panel (d): Plot of SVBW test statistic (y axis) against SMBW test statistic (x axis), for each SNP pair.
(TIF)

Figure S11 Panel (a): Plot of SMBW test statistic (y axis) against Adjusted Wu test statistic (x axis), for each SNP pair. Panel (b): Plot of SVBW test statistic (y axis) against Adjusted Wu test statistic (x axis), for each SNP pair. Panel (c): Plot of SMBW test statistic (y axis) against Joint Effects test statistic (x axis), for each SNP pair. Panel (d): Plot of SVBW test statistic (y axis) against Joint Effects test statistic (x axis), for each SNP pair.
(TIF)

Figure S12 SMBW (left hand panels (a) and (c)) and SVBW (right hand panels (b) and (d)) results, from haplotypes estimated by applying SHAPEIT to cases and controls separately (y axes) or together (x axes). Points shown in red on top panels (a) and (b) correspond to SNP pairs where the SNPs are less than 1 cM apart. These points have been removed from the plots shown in bottom panels (c) and (d).
(TIF)

Text S1 Details of the variance calculation for our various proposed statistics.
(PDF)

Text S2 Here we demonstrate that several of the test statistics described in this manuscript may show sensitivity to the presence of main effects at one or both loci, rather than showing sensitivity purely to interaction effects.
(PDF)

Text S3 A logistic regression view of the Wu et al. statistic.
(PDF)

Text S4 Here we consider the relationship between the fast-epistasis (FE) and Wellek and Ziegler (WZ) inspired statistics and standard logistic and linear regression.
(PDF)

Text S5 Here we show that the Wellek and Ziegler inspired case-only statistic can be viewed equivalently as a score test with respect to the interaction parameter δ in the model given in the second table on page 5 of Text S3.
(PDF)
