# Supplementary figures and images for: Improved Statistics for Genome-Wide Interaction Analysis
Source: PLoS Genet. 2012 Apr 5;8(4):e1002625. doi: 10.1371/journal.pgen.1002625 (PMC3320596; doi:10.1371/journal.pgen.1002625)

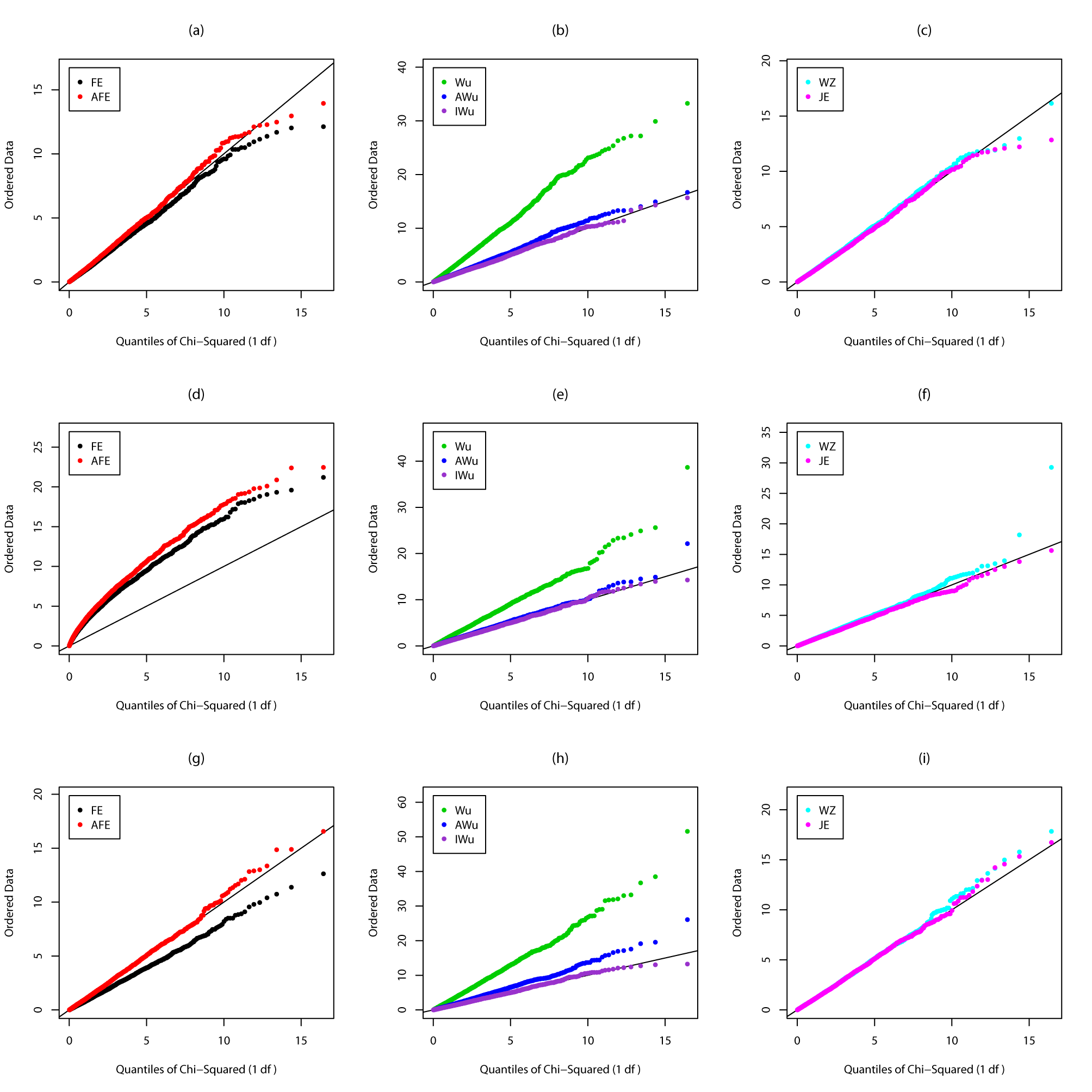

Supplement: Figure S1 — Chi-squared (1 df) Q-Q plot for Scenario 3 (Dominant effect at locus G). Top panels ((a), (b) and (c)): Case/Control not in LD; Middle panels ((d), (e) and (f)): Case/Control in LD; Bottom panels ((g), (h) and (i)): Case-Only not in LD; FE: Fast-Epistasis; AFE: Adjusted FE; Wu: Wu et al. statistic; AWu: Adjusted Wu statistic; IWu: Ideal Wu statistic; WZ: Wellek and Ziegler statistic; JE: Joint Effects statistic. (TIF) [file pgen.1002625.s001.tif]

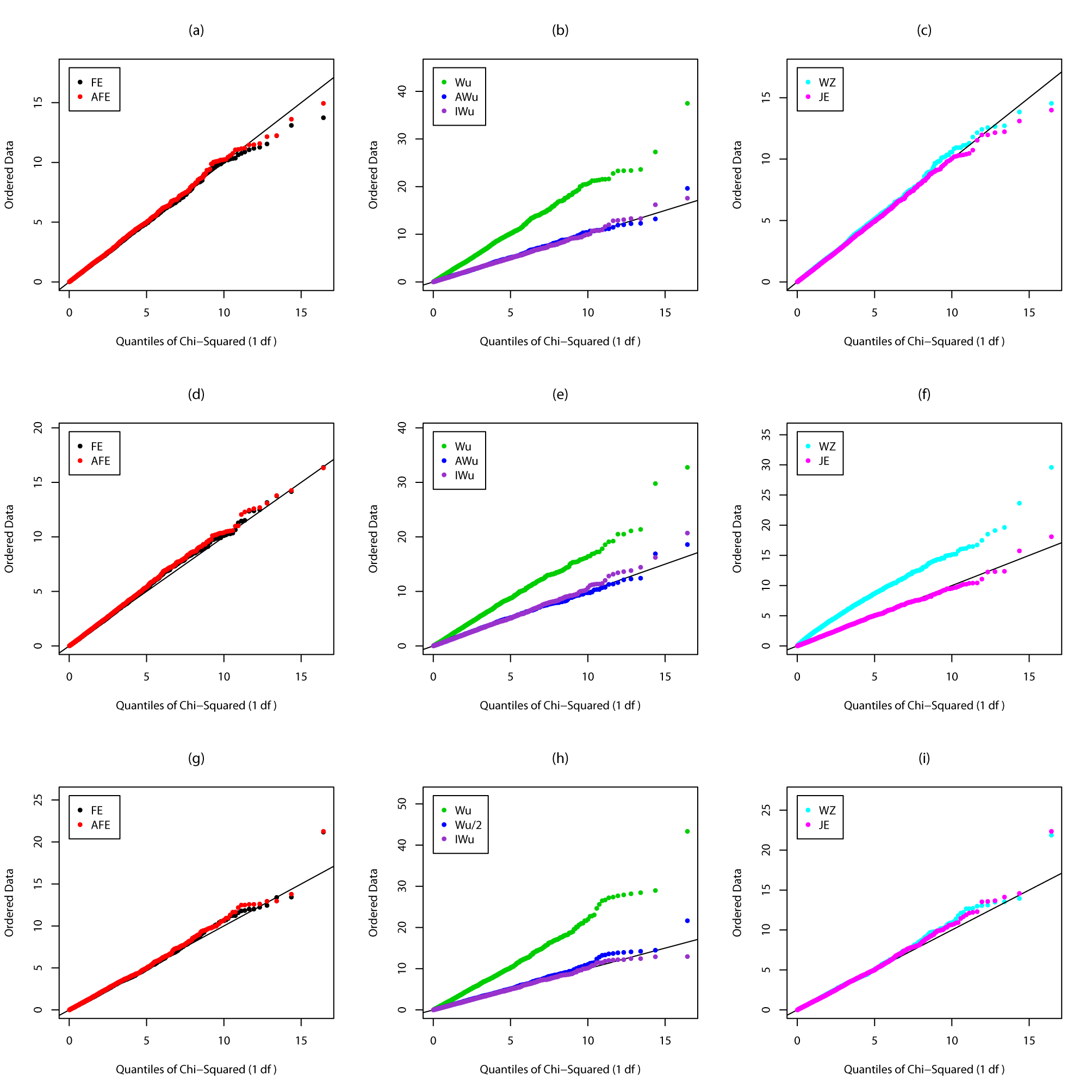

Supplement: Figure S2 — Chi-squared (1 df) Q-Q plot for Scenario 4 (Additive effect at locus G). Top panels ((a), (b) and (c)): Case/Control not in LD; Middle panels ((d), (e) and (f)): Case/Control in LD; Bottom panels ((g), (h) and (i)): Case-Only not in LD; FE: Fast-Epistasis; AFE: Adjusted FE; Wu: Wu et al. statistic; AWu: Adjusted Wu statistic; IWu: Ideal Wu statistic; WZ: Wellek and Ziegler statistic; JE: Joint Effects statistic. (TIF) [file pgen.1002625.s002.tif]

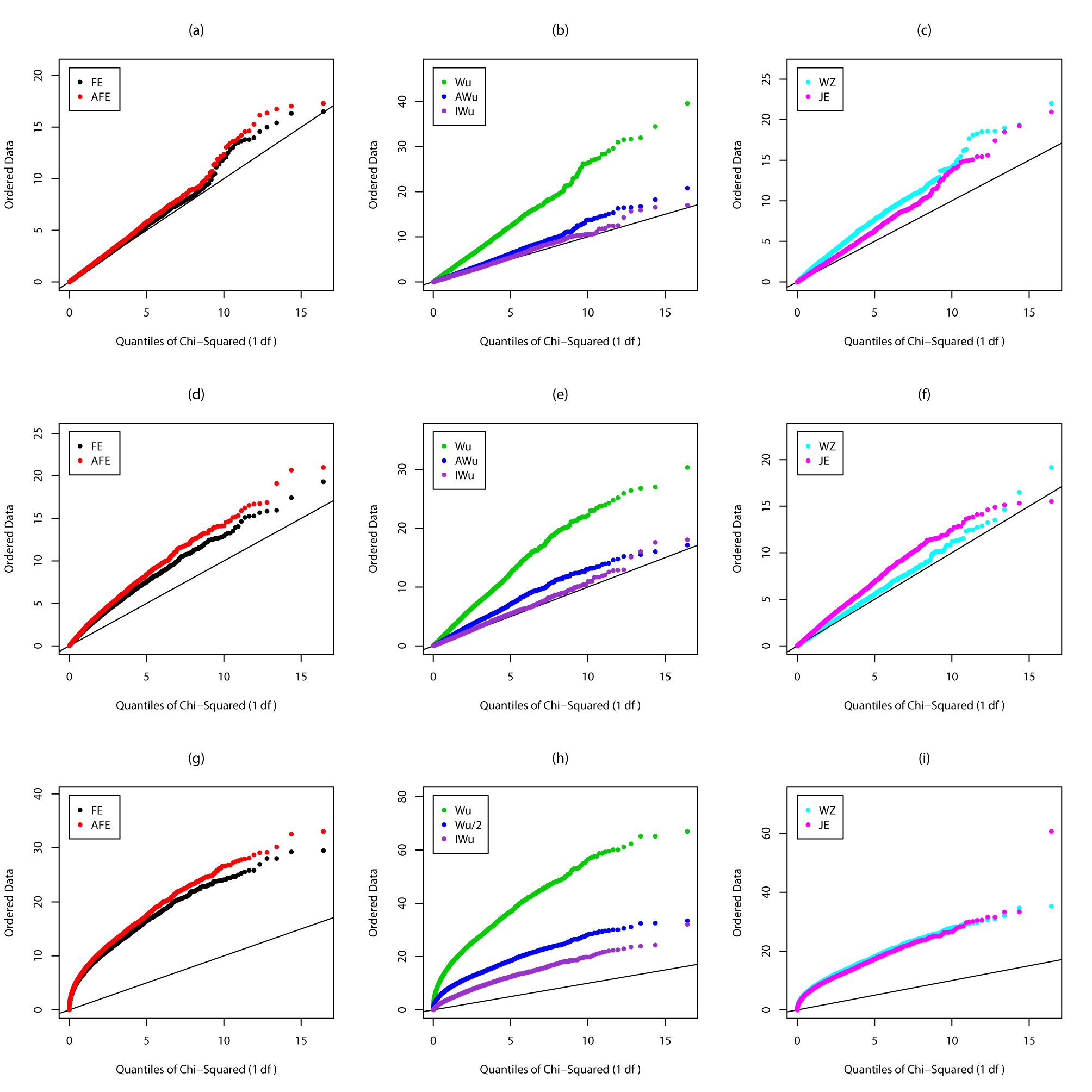

Supplement: Figure S3 — Chi-squared (1 df) Q-Q plot for Scenario 5a (Additive effects at both loci). Top panels ((a), (b) and (c)): Case/Control not in LD; Middle panels ((d), (e) and (f)): Case/Control in LD; Bottom panels ((g), (h) and (i)): Case-Only not in LD; FE: Fast-Epistasis; AFE: Adjusted FE; Wu: Wu et al. statistic; AWu: Adjusted Wu statistic; IWu: Ideal Wu statistic; WZ: Wellek and Ziegler statistic; JE: Joint Effects statistic. (TIF) [file pgen.1002625.s003.tif]

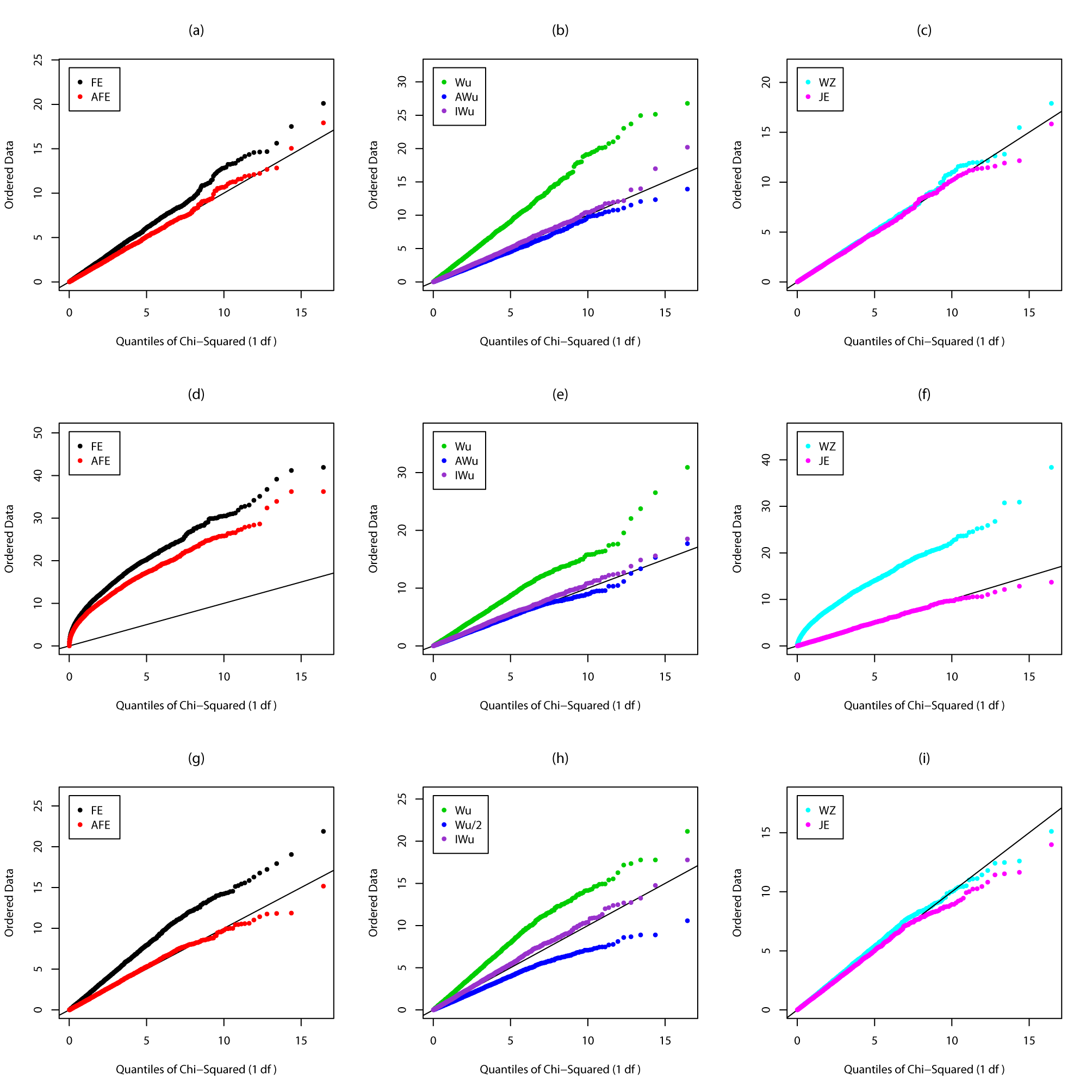

Supplement: Figure S4 — Chi-squared (1 df) Q-Q plot for Scenario 5b (Recessive effects at both loci). Top panels ((a), (b) and (c)): Case/Control not in LD; Middle panels ((d), (e) and (f)): Case/Control in LD; Bottom panels ((g), (h) and (i)): Case-Only not in LD; FE: Fast-Epistasis; AFE: Adjusted FE; Wu: Wu et al. statistic; AWu: Adjusted Wu statistic; IWu: Ideal Wu statistic; WZ: Wellek and Ziegler statistic; JE: Joint Effects statistic. (TIF) [file pgen.1002625.s004.tif]

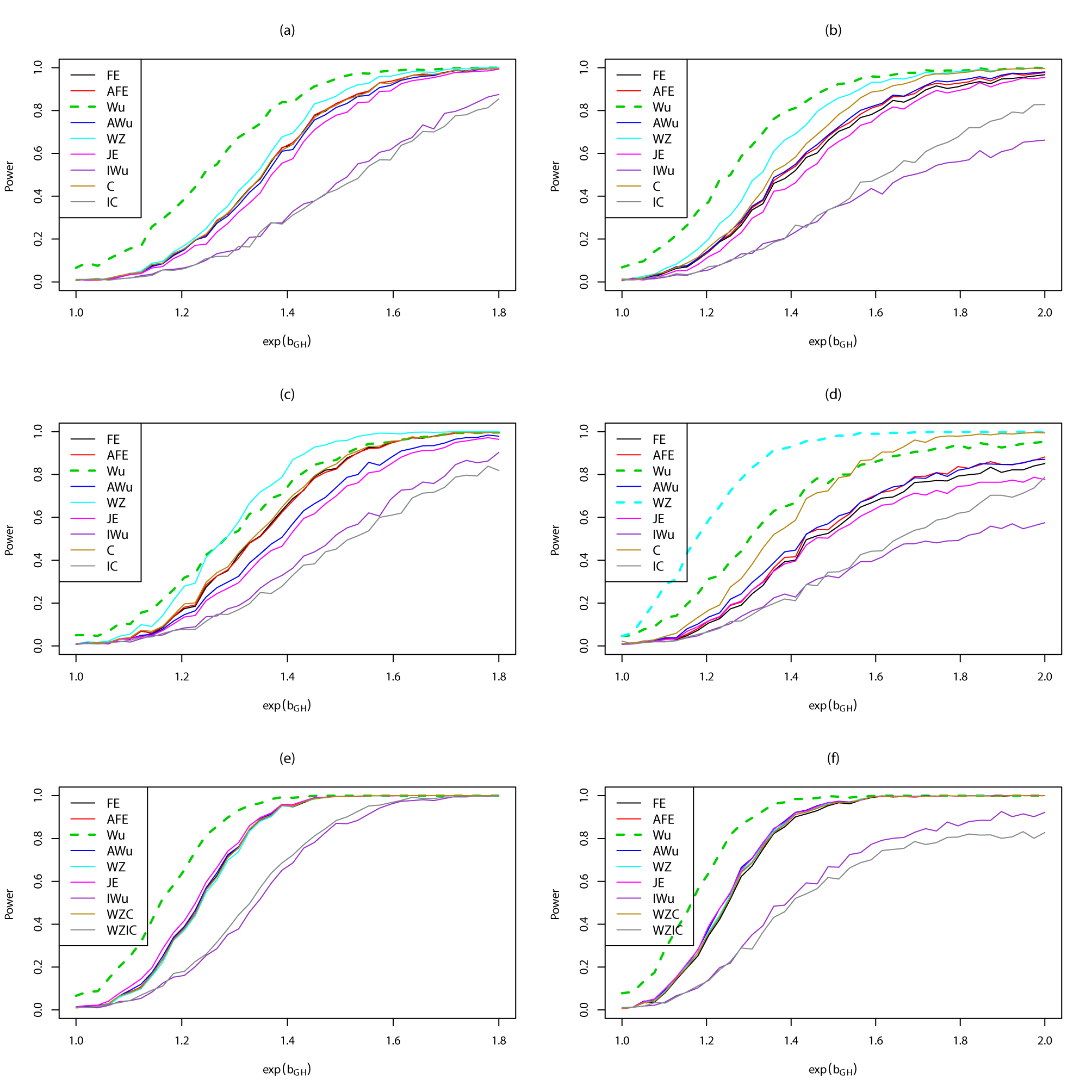

Supplement: Figure S5 — Power curves for Scenario 8 (AdditiveAdditive). Power to achieve significance level . Top panels ((a) and (b)): Case/Control not in LD; Middle panels ((c) and (d)): Case/Control in LD; Bottom panels ((e) and (f)): Case-Only not in LD; Left hand panels ((a), (c) and (e)): No main effect; Right hand panels ((b), (d) and (f)): Locus G has main effect; FE: Fast-Epistasis; AFE: Adjusted FE; Wu: Wu et al. statistic; AWu: Adjusted Wu statistic; WZ: Wellek and Ziegler statistic; JE: Joint Effects statistic; IWu: Ideal Wu statistic; C: Logistic regression using correct coding; IC: Logistic regression using incorrect ( = AdditiveRecessive) coding; WZC: Wellek and Ziegler case-only statistic using correct coding; WZIC: Wellek and Ziegler case-only statistic using incorrect ( = AdditiveRecessive) coding. (TIF) [file pgen.1002625.s005.tif]

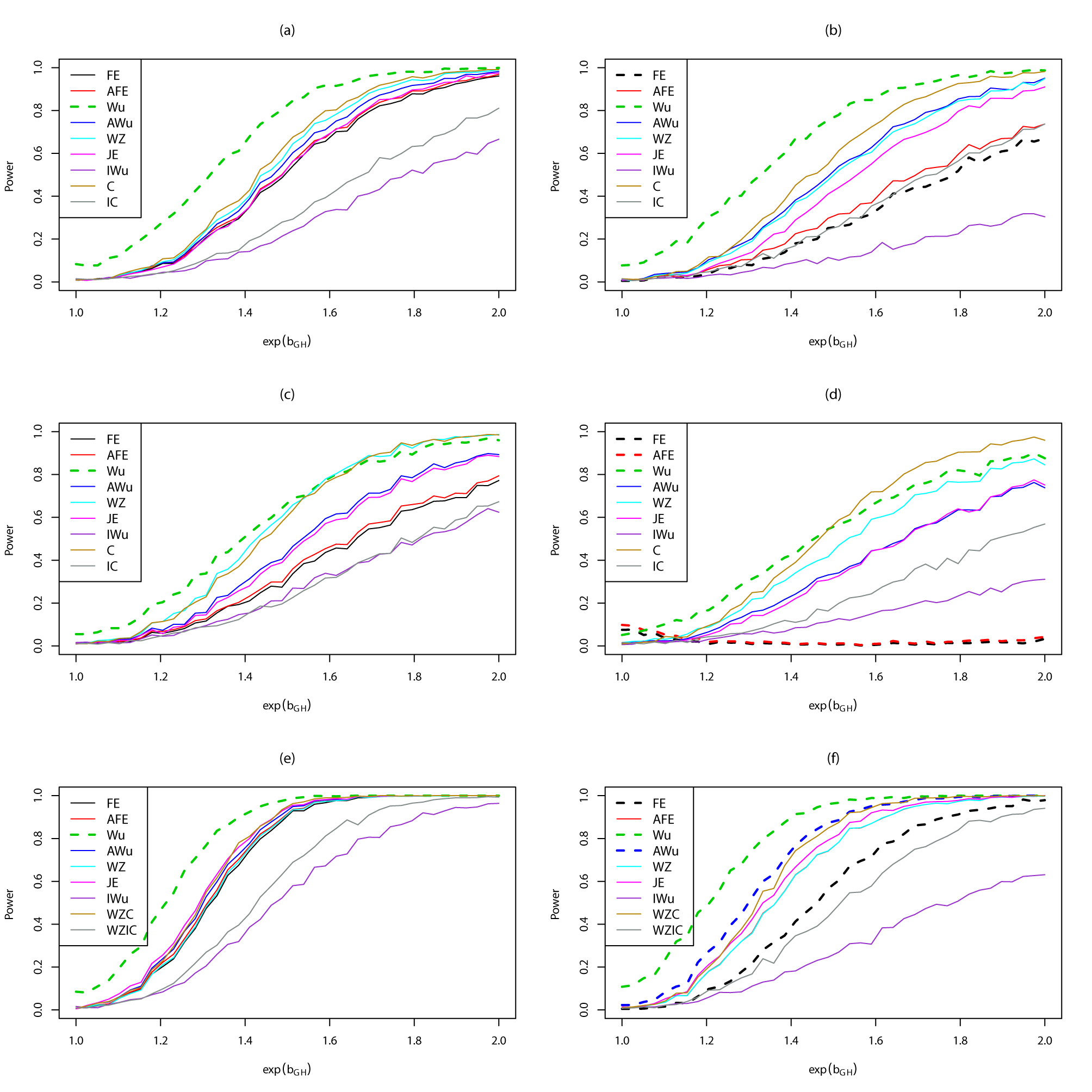

Supplement: Figure S6 — Power curves for Scenario 9 (DominantAdditive). Power to achieve significance level . Top panels ((a) and (b)): Case/Control not in LD; Middle panels ((c) and (d)): Case/Control in LD; Bottom panels ((e) and (f)): Case-Only not in LD; Left hand panels ((a), (c) and (e)): No main effect; Right hand panels ((b), (d) and (f)): Locus G has main effect; FE: Fast-Epistasis; AFE: Adjusted FE; Wu: Wu et al. statistic; AWu: Adjusted Wu statistic; WZ: Wellek and Ziegler statistic; JE: Joint Effects statistic; IWu: Ideal Wu statistic; C: Logistic regression using correct coding; IC: Logistic regression using incorrect ( = DominantRecessive) coding; WZC: Wellek and Ziegler case-only statistic using correct coding; WZIC: Wellek and Ziegler case-only statistic using incorrect ( = DominantRecessive) coding. (TIF) [file pgen.1002625.s006.tif]

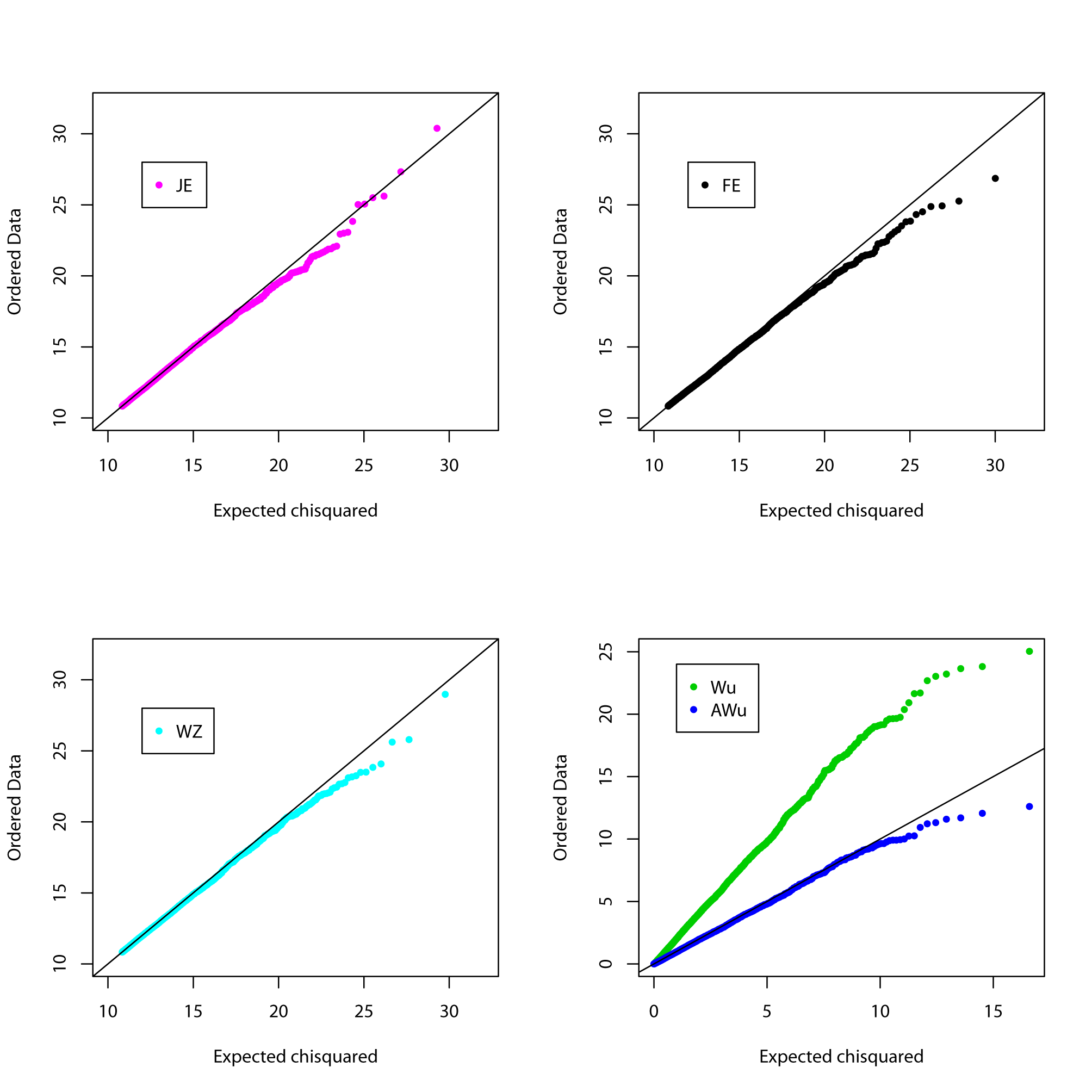

Supplement: Figure S7 — QQ plots from analysis of pairwise (SNPSNP) interactions on chromosome 22 in the WTCCC Crohn's data set. JE: Joint Effects statistic; FE: Fast-Epistasis; WZ: Wellek and Ziegler statistic; Wu: Wu et al. statistic; AWu: Adjusted Wu statistic. (TIF) [file pgen.1002625.s007.tif]

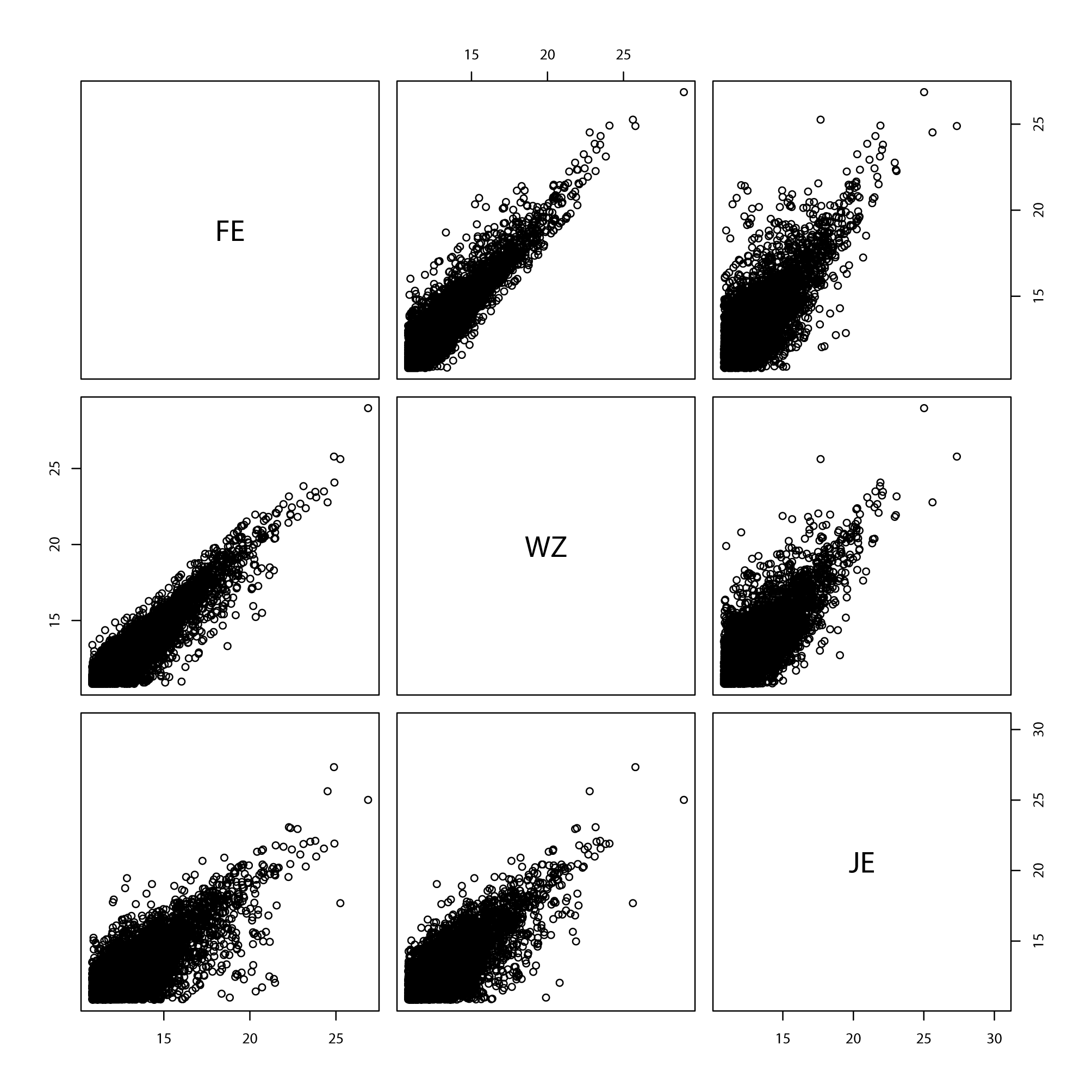

Supplement: Figure S8 — Correlations between three different methods applied to the WTCCC Crohn's data set. Each point represents a particular SNPSNP pair on chromosome 22. Shown are pairwise plots of the test statistics generated by each of the three methods. JE: Joint Effects statistic; FE: Fast-Epistasis; WZ: Wellek and Ziegler statistic. (TIF) [file pgen.1002625.s008.tif]

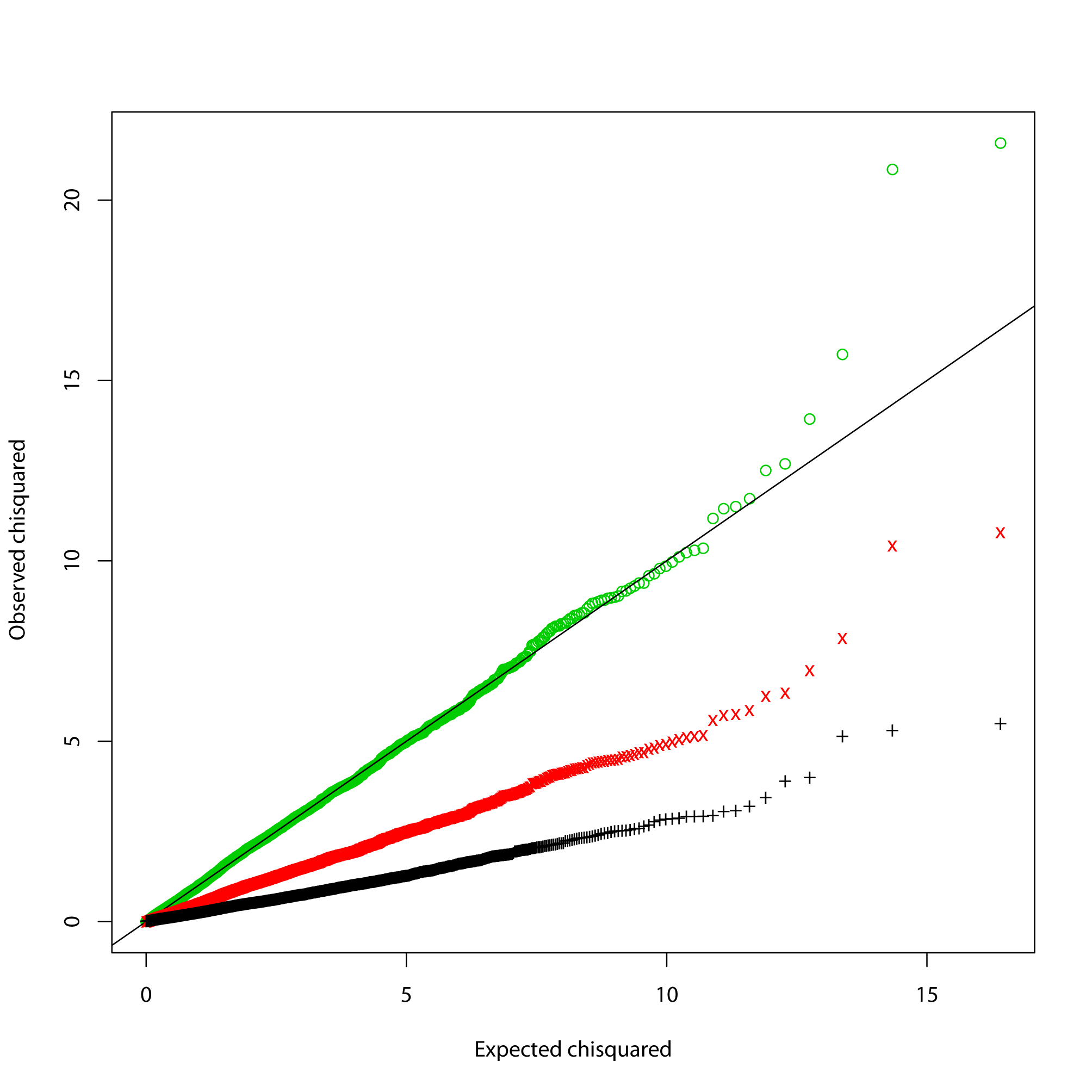

Supplement: Figure S9 — QQ plots from analyses based on the mean haplotype frequencies (over 100 replicates) estimated using SHAPEIT. Red crosses denote results calculated using the original Wu formula. Black plusses denote results calculated using the Adjusted Wu formula. Green circles denote results calculated using the SMBW formula, which corresponds to twice the original Wu statistic. (TIF) [file pgen.1002625.s009.tif]

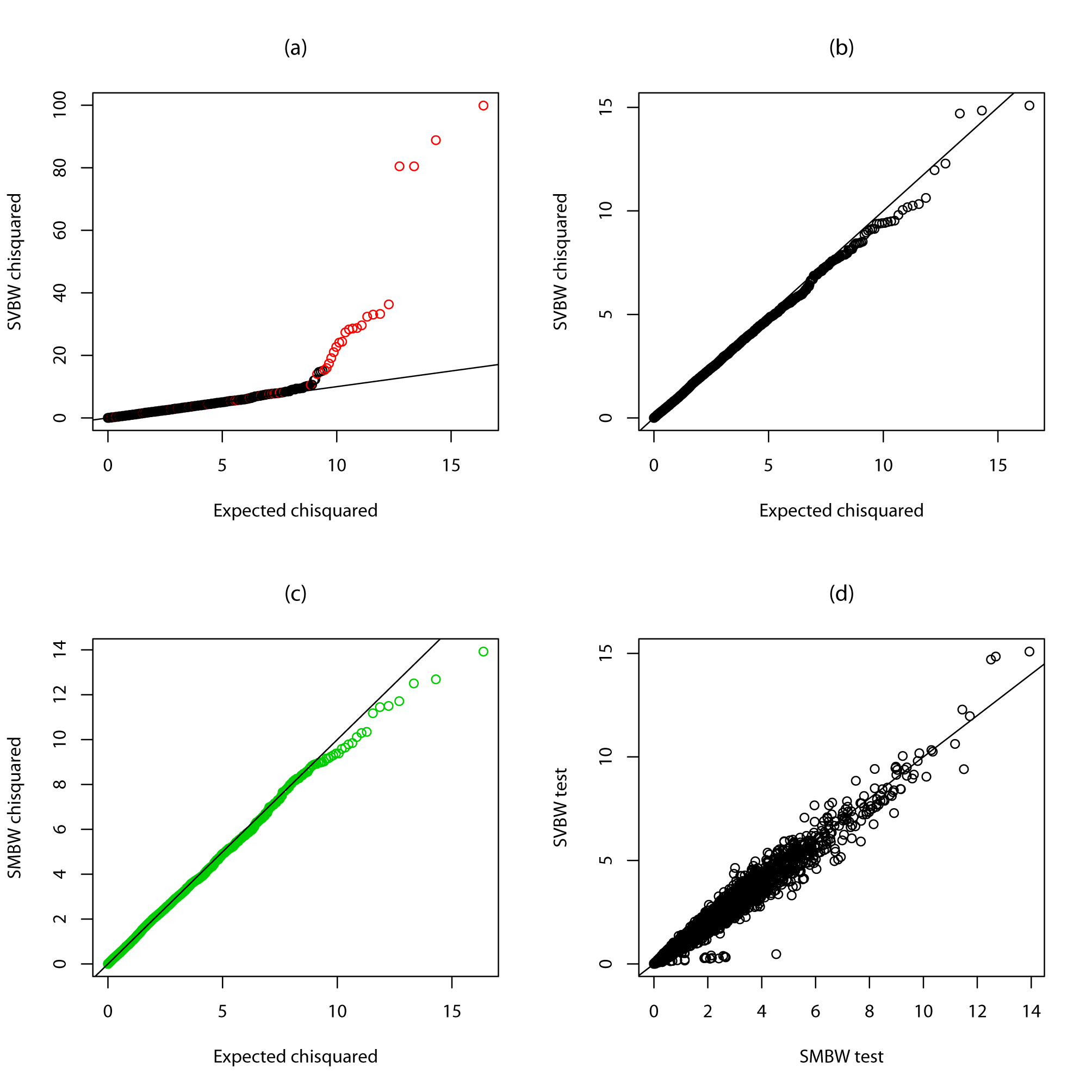

Supplement: Figure S10 — Panel (a): QQ plots from analyses based on the SVBW test statistic. Results from SNP pairs where the SNPs are less than 1 cM apart are shown in red. Panel (b): QQ plots from analyses based on the SVBW test statistic, having removed all SNP pairs where the SNPs are less than 1 cM apart. Panel (c): QQ plots from analyses based on the SMBW test statistic, having removed all SNP pairs where the SNPs are less than 1 cM apart. Panel (d): Plot of SVBW test statistic (y axis) against SMBW test statistic (x axis), for each SNP pair. (TIF) [file pgen.1002625.s010.tif]

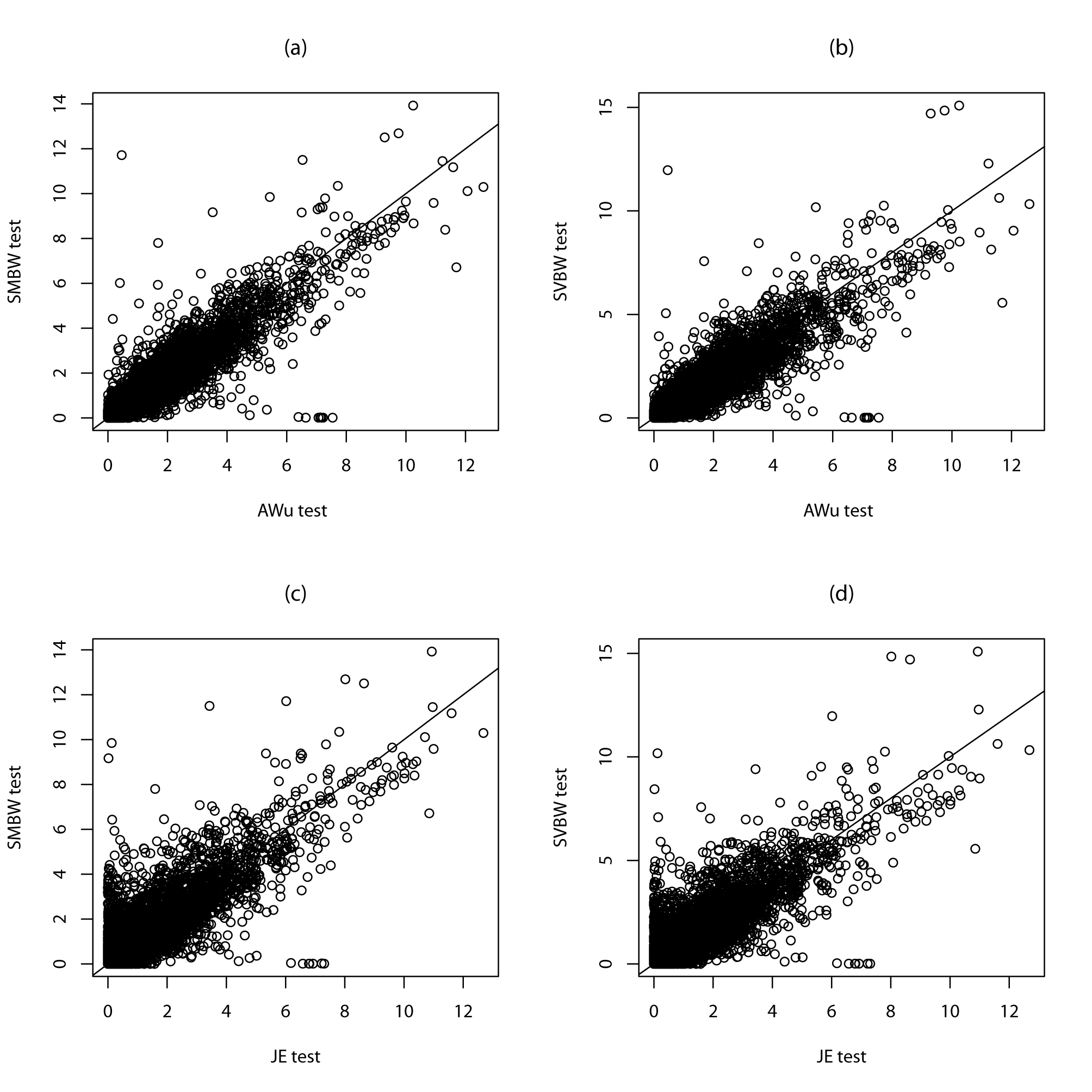

Supplement: Figure S11 — Panel (a): Plot of SMBW test statistic (y axis) against Adjusted Wu test statistic (x axis), for each SNP pair. Panel (b): Plot of SVBW test statistic (y axis) against Adjusted Wu test statistic (x axis), for each SNP pair. Panel (c): Plot of SMBW test statistic (y axis) against Joint Effects test statistic (x axis), for each SNP pair. Panel (d): Plot of SVBW test statistic (y axis) against Joint Effects test statistic (x axis), for each SNP pair. (TIF) [file pgen.1002625.s011.tif]

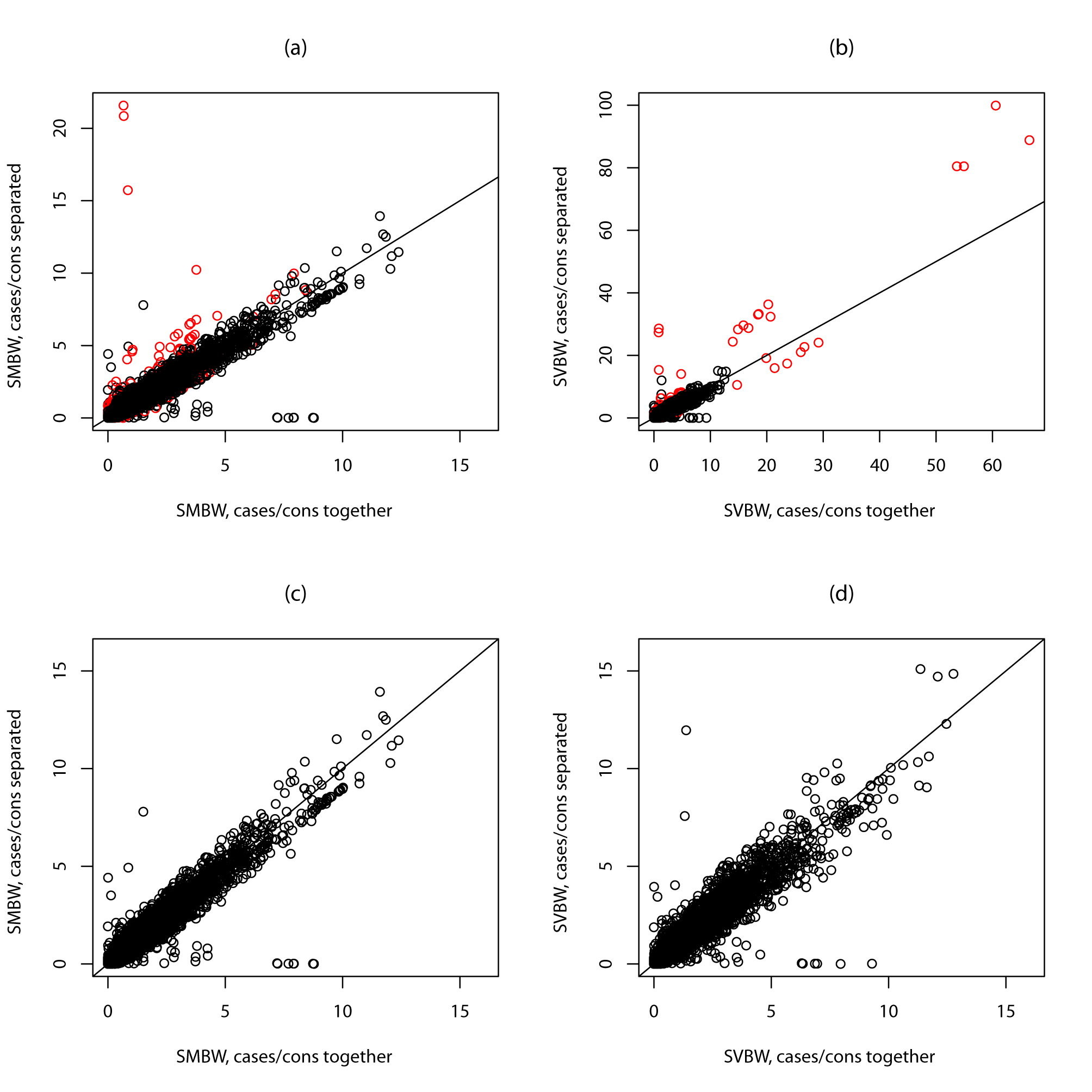

Supplement: Figure S12 — SMBW (left hand panels (a) and (c)) and SVBW (right hand panels (b) and (d)) results, from haplotypes estimated by applying SHAPEIT to cases and controls separately (y axes) or together (x axes). Points shown in red on top panels (a) and (b) correspond to SNP pairs where the SNPs are less than 1 cM apart. These points have been removed from the plots shown in bottom panels (c) and (d). (TIF) [file pgen.1002625.s012.tif]
