# Supplementary material for: Improved Statistics for Genome-Wide Interaction Analysis
Source: PLoS Genet. 2012 Apr 5;8(4):e1002625. doi: 10.1371/journal.pgen.1002625 (PMC3320596; doi:10.1371/journal.pgen.1002625)
Supplement: Text S1 — Details of the variance calculation for our various proposed statistics. (PDF) [file pgen.1002625.s013.pdf]

Text S1

Here we provide details of the variance calculation for our various proposed statistics.

Variance calculation for adjusted Wu statistic

We have

$$\lambda(\theta) = \log \frac{P_{11}P_{22}}{P_{12}P_{21}},$$

where haplotype frequencies $P_{jk} = P(G_j\text{-}H_k)$ for $j,k = 1,2$. We reparameterize P_{ij} by a parameter vector $\theta = (p,u,D)^T$ where $P_{11} = pu + D$, $P_{12} = pv - D$, $P_{21} = qu - D$ and $P_{22} = qv + D$, and where $q = 1 - p$ and $v = 1 - u$.

The asymptotic variance of MLE $\hat{\theta}$ was given by Brown [1] and application of the delta method readily leads to the asymptotic variance of $\lambda(\hat{\theta})$. To this end, we introduce some notation:

$$\phi = \{P_{11}P_{22}(P_{11}+P_{22}) + P_{12}P_{21}(P_{12}+P_{21})\}/\{(P_{11}P_{22}+P_{12}P_{21})P_{11}P_{12}P_{21}P_{22}\},$$

$$\omega = [\phi^{-1} + D^2\{pq(v-u)^2 + uv(q-p)^2\} - 2D^3(q-p)(v-u)]/(pquv - D^2),$$

$$\Sigma = 2^{-1}\begin{pmatrix} pq & D & D(q-p) \\ D & uv & D(v-u) \\ D(q-p) & D(v-u) & \omega \end{pmatrix}$$

and

$$\frac{\partial\lambda}{\partial\theta} = \begin{pmatrix} uP_{11}^{-1} - vP_{12}^{-1} + uP_{21}^{-1} - vP_{22}^{-1} \\ pP_{11}^{-1} + pP_{12}^{-1} - qP_{21}^{-1} - qP_{22}^{-1} \\ P_{11}^{-1} + P_{12}^{-1} + P_{21}^{-1} + P_{22}^{-1} \end{pmatrix}$$

Now $n^{-1}\Sigma$ coincides with the asymptotic variance of $\hat{\theta}$ derived in [1], in which n represents the number of individuals. It follows from the delta method that the asymptotic variance of $\lambda(\hat{\theta})$, v, is written as

$$v = n^{-1}\frac{\partial\lambda}{\partial\theta^T}\Sigma\frac{\partial\lambda}{\partial\theta}$$

The variance v can be consistently estimated by $\hat{v}$, in which we substitute $\hat{\theta}$ for the unknown parameters appearing in v. If we assume $D = 0$, v reduces to $n^{-1}(P_{11}^{-1} + P_{12}^{-1} + P_{21}^{-1} + P_{22}^{-1})$, which is exactly double

the variance estimate derived by Wu et al. [2].

The original Wu statistic, as well as our adjusted version, is potentially undefined if one of the estimated haplotype frequencies $P_{jk} = 0$. To avoid this issue, in our calculation of the original and adjusted Wu statistics we set any haplotype frequency estimated as $< 10^{-8}$ to be equal to 10^{-8}.

Variance calculation for adjusted *fast-epistasis* statistic

We define a 9-dimensional vector p to denote the genotype frequencies (probabilities) at two loci G and H, as observed in a sample of individuals, $p = (q_{00}, q_{01}, \ldots, q_{22})^T$. (Here q_{kl} corresponds to the observed sample frequency of carrying k copies of allele G_1 and l copies of allele H_1 at loci G and H respectively, derived from counts as shown, for example, in Table 1).

We define 4×9 matrix M by

$$M = 4^{-1} \begin{pmatrix} 4 & 2 & 0 & 2 & 1 & 0 & 0 & 0 & 0 \\ 0 & 2 & 4 & 0 & 1 & 2 & 0 & 0 & 0 \\ 0 & 0 & 0 & 2 & 1 & 0 & 4 & 2 & 0 \\ 0 & 0 & 0 & 0 & 1 & 2 & 0 & 2 & 4 \end{pmatrix}$$

and let $\tilde{P} \equiv (\tilde{P}_A, \tilde{P}_B, \tilde{P}_C, \tilde{P}_D)^T = Mp$. Then the log odds ratio used to calculate the *fast-epistasis* statistic may be written as

$$\lambda_{\text{FE}} = \log \frac{\tilde{P}_A \tilde{P}_D}{\tilde{P}_B \tilde{P}_C}. \tag{1}$$

According to [3], the variance of λ_{FE} is given as

$$V' \equiv \frac{1}{4n}(\tilde{P}_A^{-1} + \tilde{P}_B^{-1} + \tilde{P}_C^{-1} + \tilde{P}_D^{-1}) \tag{2}$$

in our notation, where n is the number of individuals. However, this equation holds only when the three quantities $(\tilde{P}_A, \tilde{P}_B, \tilde{P}_C)$ are independent, which is not in fact the case.

To derive the asymptotic variance of $\tilde{P}$, we rely on the delta method. Recall that λ_{FE} is a function of p. Let $\dot{t}$ be the first derivative of λ_{FE} with respect to p, and let Σ_p be the 9×9 covariance matrix of p which equals $D_p - pp^T$, where $D_p = \text{diag}(p)$. It follows from the chain rule that $\dot{t} = (\partial \tilde{P}/\partial p^T)(\partial \lambda_{\text{FE}}/\partial \tilde{P})$. From $\tilde{P} = Mp$, we have $\partial \tilde{P}/\partial p^T = M$, while from Equation (1), we have

$d \equiv \partial\lambda_{\mathrm{FE}}/\partial\tilde{P} = (\tilde{P}_A^{-1}, -\tilde{P}_B^{-1}, -\tilde{P}_C^{-1}, \tilde{P}_D^{-1})^T$. Thus, it holds that $p^T\dot{t} = \tilde{P}^T d = 0$. Using this notation, we can write the asymptotic variance as

$$\mathrm{var}(\lambda_{\mathrm{FE}}) = n^{-1}\dot{t}^T\Sigma_p\dot{t} = n^{-1}d^T M D_p M^T d. \tag{3}$$

Equation (3) indeed coincides with the variance in Equation (2) assumed by PLINK [3], if the two loci are in Hardy-Weinberg equilibrium (HWE) and not in linkage disequilibrium (LD) within the sample (e.g. cases or controls) under consideration. To see this, we note that the difference between two variance quantities is given by

$$\Delta \equiv V' - \mathrm{var}(\lambda_{\mathrm{FE}}) = n^{-1}d^T S d,$$

where

$$S = 4^{-1}\begin{pmatrix} 3q_{22} + q_{21}/2 + q_{12}/2 & q_{21} + q_{11}/4 & q_{12} + q_{11}/4 & q_{11}/4 \\ q_{21} + q_{11}/4 & 3q_{20} + q_{21}/2 + q_{10}/2 & q_{11}/4 & q_{10} + q_{11}/4 \\ q_{12} + q_{11}/4 & q_{11}/4 & 3q_{02} + q_{12}/2 + q_{01}/2 & q_{01} + q_{11}/4 \\ q_{11}/4 & q_{10} + q_{11}/4 & q_{01} + q_{11}/4 & 3q_{00} + q_{10}/2 + q_{01}/2 \end{pmatrix}.$$

When two loci are not in LD within the sample under consideration (i.e. $P_{11}P_{22} - P_{12}P_{21} = 0$), we have $d = (P_{11}^{-1}, -P_{12}^{-1}, -P_{21}^{-1}, P_{22}^{-1})^T$. Then, the first element of 4-dimensional verctor Sd reduces to

$$P_{11}^{-1}(3P_{11} + P_{12} + P_{21})P_{11} - P_{12}^{-1}(2P_{11} + P_{21})P_{12} - P_{21}^{-1}(2P_{11} + P_{12})P_{21} + P_{11}^{-1}P_{22}P_{22} = 0.$$

Similarly, the other three elements are shown to be zero, which implies that $Sd = 0$. As a consequence, we can prove that

$$\Delta = n^{-1}d^T S d = 0.$$

In the general situation, Δ is not always zero. The following table illustrates the variance formulae (3) and (2) evaluated under Scenarios 1–5a used for type 1 error simulations:

		Case		Control	
Scenario	NoLD/LD	nV'	$n\mathrm{var}(\lambda_{\mathrm{FE}})$	nV'	$n\mathrm{var}(\lambda_{\mathrm{FE}})$
1	No LD	7.44	7.44	7.44	7.44
	LD	6.78	6.68	6.78	6.68
2	No LD	6.25	7.62	7.48	7.42
	LD	5.67	6.97	6.81	6.66
3	No LD	5.28	4.12	7.58	7.63
	LD	4.84	3.74	6.9	6.85
4	No LD	4.91	4.83	7.68	7.6
	LD	4.5	4.47	7	6.8
5a	No LD	4.25	3.94	8.52	8.1
	LD	4.2	3.82	7.76	7
1b	LD	9.01	7.92	9.01	7.92

Therefore, the difference between the variances is not zero except for in the no LD case of Scenario 1.

Finally, we give an additional simulation study (Scenario 1b) where the difference between Equations (3) and (2) is investigated. The setting of Scenario 1b is the same as in the LD situation in Scenario 1, except that the true haplotype frequencies in the general population are $\psi_{11} = 0.15, \psi_{12} = 0.05, \psi_{21} = 0.01$ and $\psi_{22} = 0.79$, giving $\rho = 0.805$ and theoretical variance quantities $nV' = 9.01$ and $n\mathrm{var}(\lambda_{\mathrm{FE}}) = 7.92$ (in both cases and controls), see table above. We used 100,000 replicates with 1,000 cases and 1,000 controls. The type 1 error rates for case-control statistics of PLINK's fast-epistatsis and our adjusted fast-epistasis are summarized as follows, which indicates that the correct asymptotic variance given in Equation (3) works well, whereas that used by PLINK results in less than the nominal type 1 error rates i.e. in a conservative test.

Nominal error rate	FE	AFE
0.05	0.0362	0.0493
0.01	0.0058	0.0099
0.005	0.0027	0.0047
0.001	0.0004	0.001

Variance calculation and calculation of weights for joint effects statistic

The asymptotic variance of $\xi = (\log \hat{i}_{22}, \log \hat{i}_{21}, \log \hat{i}_{12}, \log \hat{i}_{11})^T$ is given as

$$C = n^{-1} \begin{pmatrix} q_{22}^{-1} + q_{02}^{-1} + q_{20}^{-1} + q_{00}^{-1} & q_{20}^{-1} + q_{00}^{-1} & q_{02}^{-1} + q_{00}^{-1} & q_{00}^{-1} \\ q_{20}^{-1} + q_{00}^{-1} & q_{21}^{-1} + q_{20}^{-1} + q_{01}^{-1} + q_{00}^{-1} & q_{00}^{-1} & q_{01}^{-1} + q_{00}^{-1} \\ q_{02}^{-1} + q_{00}^{-1} & q_{00}^{-1} & q_{12}^{-1} + q_{10}^{-1} + q_{02}^{-1} + q_{00}^{-1} & q_{10}^{-1} + q_{00}^{-1} \\ q_{00}^{-1} & q_{01}^{-1} + q_{00}^{-1} & q_{10}^{-1} + q_{00}^{-1} & q_{11}^{-1} + q_{10}^{-1} + q_{01}^{-1} + q_{00}^{-1} \end{pmatrix},$$

where n is the sample size. The delta method allows us to derive the asymptotic variance of $f(\xi)$ with any transformation f as

$$\frac{\partial f}{\partial \xi^T} C \frac{\partial f}{\partial \xi}. \tag{4}$$

For calculation of asymptotic variance of λ, we apply the above formula to the 4-dimensional vector-valued function $f(\xi) = (\xi_1/2, \xi_2, \xi_3, \log(2e^{\xi_4} - 1))^T$. Then $\frac{\partial f}{\partial \xi} = \mathrm{diag}(1/2, 1, 1, 2e^{\xi_4}/(2e^{\xi_4} - 1))$. On the other hand, for calculation of asymptotic variance of μ, we apply the above formula to the 4-dimensional vector-valued function $f(\xi) = (\log(\sqrt{e^{\xi_1}} + 1) - \log 2,\ \log(e^{\xi_2} + 1) - \log 2,\ \log(e^{\xi_3} + 1) - \log 2,\ \xi_4)^T$. Then we have $\frac{\partial f}{\partial \xi} = \mathrm{diag}(\sqrt{e^{\xi_1}}/(2\sqrt{e^{\xi_1}} + 2), e^{\xi_2}/(e^{\xi_2} + 1), e^{\xi_3}/(e^{\xi_3} + 1), 1)$. Substituting these equations in (4) we get the expression for their asymptotic variances.

The joint effects test estimates $\lambda = \lambda(\theta)$ via a weighted average:

$$\tilde{\lambda} = w_{22} \frac{\log \hat{i}_{22}}{2} + w_{21} \log \hat{i}_{21} + w_{12} \log \hat{i}_{12} + w_{11} \log(2\hat{i}_{11} - 1), \tag{5}$$

where $w = (w_{22}, w_{21}, w_{12}, w_{11})^T$ is the weight vector that sums to 1, and $\hat{i}$ represent estimates obtained from Equation (16) in the main manuscript. The weights are determined to make the variance minimum. Write the asymptotic covariance matrix of $(\log \hat{i}_{22}/2, \log \hat{i}_{21}, \log \hat{i}_{12}, \log(2\hat{i}_{11} - 1))$ as V which corresponds to Equation (4). Then, the weights that minimise the variance of $\tilde{\lambda}$ are given by

$$w = (1^T V^{-1} 1)^{-1} V^{-1} 1,$$

where 1 is the 4-dimensional vector of ones. The minimum of the variance of $\tilde{\lambda}$ is attained at $(1^T V^{-1} 1)^{-1}$, which is estimated consistently by substituting in the estimates $\hat{i}_{jk}$.

In practice, the joint effects statistic can potentially be affected by small sample issues. To avoid this, when calculating the joint effects statistic, if any cells in case or control tables (Table 1 in main manuscript) have a count of zero, we add a count of 0.5 to all cells both in cases and controls. (We implement a similar approach when calculating the Wellek and Ziegler statistic). In addition, for the joint effects statistic, if the baseline (bottom right) cell of Table 1 has frequency < 0.01, we set its frequency to equal 0.01 then re-standardize to make the summation unity.

References

1. Brown A (1975) Sample sizes required to detect linkage disequilibrium between two or three loci. Theoretical Population Biology 8: 184–201.

2. Wu X, Dong H, Luo L, Zhu Y, Peng G, et al. (2010) A novel statistic for genome-wide interaction analysis. PLoS Genet 6: e1001131.

3. Purcell S, Neale B, Todd-Brown K, Thomas L, Ferreira MA, et al. (2007) PLINK: a tool set for whole-genome association and population-based linkage analyses. Am J Hum Genet 81: 559–575.
