# Supplementary material for: Improved Statistics for Genome-Wide Interaction Analysis
Source: PLoS Genet. 2012 Apr 5;8(4):e1002625. doi: 10.1371/journal.pgen.1002625 (PMC3320596; doi:10.1371/journal.pgen.1002625)
Supplement: Text S2 — Here we demonstrate that several of the test statistics described in this manuscript may show sensitivity to the presence of main effects at one or both loci, rather than showing sensitivity purely to interaction effects. (PDF) [file pgen.1002625.s014.pdf]

Text S2

Here we demonstrate that several of the test statistics described in this manuscript may show sensitivity to the presence of main effects at one or both loci, rather than showing sensitivity purely to interaction effects. For simplicity, we start by considering a 2×2 table for binary variables, before extending our results to the usual 3×3 SNP genotype table. We show that the 2×2 table has a desirable property corresponding to the fact that, in the presence of a main effect, the odds ratio (representing association between the variables) is identical when calculated within a sample of cases as when calculated within a sample of controls. We then investigate to what extent this desirable property is inherited by various test statistics based on the usual 3×3 genotype table.

Invariant property of odds ratio in 2×2 table

Suppose that individuals in general population have measurements of two dichotomous variables G and H (e.g. relating to genotype at loci G and H) that take values in $\{0, 1\}$. The distribution of the variables in the general population may be expressed in the following the 2×2 table:

	H	
G	1	0
1	Q_{11}	Q_{10}
0	Q_{01}	Q_{00}

in which $Q_{jk} = P(G = j, H = k)$ for $j, k = 0, 1$.

The objective is the odds ratio representing association between the variables in the 2×2 table:

$$\frac{Q_{11}Q_{00}}{Q_{10}Q_{01}},$$

which is calculated independently for case and control samples and is used to detect interaction. Let the probability that an individual with $G = j$ and $H = k$ is affected be $f_{jk} = P(D|G = j, H = k)$, where D represents the event that an individual is affected (diseased). Under such specification, frequencies of case and control populations are respectively given by $Q_{A,jk} = \frac{f_{jk}Q_{jk}}{K}$ and $Q_{N,jk} = \frac{(1-f_{jk})Q_{jk}}{(1-K)}$, where K

denotes the prevalence, i.e., $K = P(D)$. Then, the odds ratios for case and control samples are given by

$$\frac{Q_{A,11}Q_{A,00}}{Q_{A,01}Q_{A,10}} = \frac{f_{11}f_{00}}{f_{01}f_{10}}\frac{Q_{11}Q_{00}}{Q_{01}Q_{10}},$$

and

$$\frac{Q_{N,11}Q_{N,00}}{Q_{N,01}Q_{N,10}} = \frac{(1-f_{11})(1-f_{00})}{(1-f_{01})(1-f_{10})}\frac{Q_{11}Q_{00}}{Q_{01}Q_{10}},$$

respectively. From these expressions, we can see that the odds ratios for case and control samples are in general not identical. However, if there is no effect at either locus (i.e. f_{jk} is constant), or if only one of the loci has a main effect, they are identical.

To see this, assume that $f_{10} = f_{11} = a = \frac{\exp(\alpha+\beta)}{1+\exp(\alpha+\beta)}$ and $f_{00} = f_{01} = b = \frac{\exp(\alpha)}{1+\exp(\alpha)}$ i.e. without loss of generality G has main effect. (The argument for H is the same). In this case, we have the following distributions in the case and control populations respectively:

Cases	H	
G	1	0
1	$\frac{aQ_{11}}{K}$	$\frac{aQ_{10}}{K}$
0	$\frac{bQ_{01}}{K}$	$\frac{bQ_{00}}{K}$

Controls	H	
G	1	0
1	$\frac{(1-a)Q_{11}}{(1-K)}$	$\frac{(1-a)Q_{10}}{(1-K)}$
0	$\frac{(1-b)Q_{01}}{(1-K)}$	$\frac{(1-b)Q_{00}}{(1-K)}$

The fact that $f_{10} = f_{11} = a$ and $f_{00} = f_{01} = b$ means that these terms (or 1- them), together with K and $(1-K)$, cancel from the odds ratios calculated from each of the above tables, and we have

$$\frac{Q_{A,11}Q_{A,00}}{Q_{A,01}Q_{A,10}} = \frac{Q_{N,11}Q_{N,00}}{Q_{N,01}Q_{N,10}} = \frac{Q_{11}Q_{00}}{Q_{01}Q_{10}}.$$

Thus, any statistic based on the difference of the odds ratio between cases and controls is valid in the presence of a main effect at one locus. In addition, any statistic based on whether the odds ratio in cases differs from 1 is valid provided $\frac{Q_{11}Q_{00}}{Q_{10}Q_{01}} = 1$, i.e. provided there is no population-level association between the variables.

Consider now the presence of main effects at both loci, so that $f_{jk} = \frac{\exp(\alpha+\beta I(j=1)+\gamma I(k=1))}{1+\exp(\alpha+\beta I(j=1)+\gamma I(k=1))}$ (where $I(E)$ represents an indicator variable for the occurence of event E). We no longer have the cancelling of f_{jk} or $(1-f_{jk})$ terms that occurs in the presence of a main effect at one locus, and thus we may observe a difference in the odds ratio between cases and controls, even when no interaction effects exist. However,

if the disease is sufficiently rare, we find that the required terms do cancel, and the odds ratio in cases is again equal to that in controls. To see this, note that for a rare disease, with main effects at both loci, we may write

$$f_{jk} = \frac{e^{\alpha+\beta I(j=1)+\gamma I(k=1)}}{1+e^{\alpha+\beta I(j=1)+\gamma I(k=1)}} \approx e^{\alpha+\beta I(j=1)+\gamma I(k=1)}$$

Thus we may write $f_{11} = e^{\alpha+\beta+\gamma} = ABC$, $f_{10} = e^{\alpha+\beta} = AB$, $f_{01} = e^{\alpha+\gamma} = AC$ and $f_{00} = e^{\alpha} = A$, where $A = e^{\alpha}$, $B = e^{\beta}$ and $C = e^{\gamma}$. We have the following distributions in the case and control populations respectively:

Cases	H	
G	1	0
1	$\frac{ABCQ_{11}}{K}$	$\frac{ABQ_{10}}{K}$
0	$\frac{ACQ_{01}}{K}$	$\frac{AQ_{00}}{K}$

Controls	H	
G	1	0
1	Q_{11}	Q_{10}
0	Q_{01}	Q_{00}

(since, under the rare disease assumption, the distribution in controls is the same as in the general population). When calculating odds ratios from each of the above tables, the A, B, C terms cancel and we have

$$\frac{Q_{A,11}Q_{A,00}}{Q_{A,01}Q_{A,10}} = \frac{Q_{N,11}Q_{N,00}}{Q_{N,01}Q_{N,10}} = \frac{Q_{11}Q_{00}}{Q_{01}Q_{10}}.$$

Thus, under a rare disease assumption, any statistic based on the difference of the odds ratio between cases and controls is valid in the presence of main effects at both loci. In addition, any statistic based on whether the odds ratio in cases differs from 1 is valid provided $\frac{Q_{11}Q_{00}}{Q_{10}Q_{01}} = 1$, i.e. provided there is no population-level association between the variables.

Invariant property of odds ratio from 3×3 table as used in various tests

Here we investigate whether or not the invariant property holds for various previously-described statistics for detecting gene-gene interaction. All statistics are calulated based on tabulating genotypes at loci G and H in cases and controls, as shown in Table 1. Assume that only G has main effect, that is, we define the penetrances by $a = P(D|G = G_1G_1) = \frac{e^{\alpha+\beta_2}}{1+e^{\alpha+\beta_2}}$, $b = P(D|G = G_1G_2) = \frac{e^{\alpha+\beta_1}}{1+e^{\alpha+\beta_1}}$ and $c = P(D|G = G_2G_2) = \frac{e^{\alpha}}{1+e^{\alpha}}$. We express the distribution of genotypes in the general population in the following the 3×3 table:

	H		
G	2	1	0
2	Q_{22}	Q_{21}	Q_{20}
1	Q_{12}	Q_{11}	Q_{10}
0	Q_{02}	Q_{01}	Q_{00}

in which $Q_{jk} = P(G = j, H = k)$ for $j, k = 0, 1, 2$, and i and j refer to the number of copies of G_1 and H_1. We then have the following genotype distributions in the case and control populations, respectively:

Cases	H		
G	2	1	0
2	$\frac{aQ_{22}}{K}$	$\frac{aQ_{21}}{K}$	$\frac{aQ_{20}}{K}$
1	$\frac{bQ_{12}}{K}$	$\frac{bQ_{11}}{K}$	$\frac{bQ_{10}}{K}$
0	$\frac{cQ_{02}}{K}$	$\frac{cQ_{01}}{K}$	$\frac{cQ_{00}}{K}$

Controls	H		
G	2	1	0
2	$\frac{(1-a)Q_{22}}{(1-K)}$	$\frac{(1-a)Q_{21}}{(1-K)}$	$\frac{(1-a)Q_{20}}{(1-K)}$
1	$\frac{(1-b)Q_{12}}{(1-K)}$	$\frac{(1-b)Q_{11}}{(1-K)}$	$\frac{(1-b)Q_{10}}{(1-K)}$
0	$\frac{(1-c)Q_{02}}{(1-K)}$	$\frac{(1-c)Q_{01}}{(1-K)}$	$\frac{(1-c)Q_{00}}{(1-K)}$

where K denotes the prevalence, i.e., $K = P(D)$.

Joint effects statistic

Our new joint effects statistic is based on deleting rows and columns in the above 3×3 tables in order to create four sets of 2×2 tables, from which the odds ratio for each of the four top left cells, relative to the bottom right cell, may be estimated. We delete, in turn, the row and column corresponding to each of the four top left cells, resulting in the following four sets of tables for cases and countrols:

Top row and left column deleted:

Cases	H	
G	1	0
1	$\frac{bQ_{11}}{K}$	$\frac{bQ_{10}}{K}$
0	$\frac{cQ_{01}}{K}$	$\frac{cQ_{00}}{K}$

Controls	H	
G	1	0
1	$\frac{(1-b)Q_{11}}{(1-K)}$	$\frac{(1-b)Q_{10}}{(1-K)}$
0	$\frac{(1-c)Q_{01}}{(1-K)}$	$\frac{(1-c)Q_{00}}{(1-K)}$

Top row and middle column deleted:

Cases	H	
G	2	0
1	$\frac{bQ_{12}}{K}$	$\frac{bQ_{10}}{K}$
0	$\frac{cQ_{02}}{K}$	$\frac{cQ_{00}}{K}$

Controls	H	
G	2	0
1	$\frac{(1-b)Q_{12}}{(1-K)}$	$\frac{(1-b)Q_{10}}{(1-K)}$
0	$\frac{(1-c)Q_{02}}{(1-K)}$	$\frac{(1-c)Q_{00}}{(1-K)}$

Middle row and left column deleted:

Cases	H	
G	1	0
2	$\frac{aQ_{21}}{K}$	$\frac{aQ_{20}}{K}$
0	$\frac{cQ_{01}}{K}$	$\frac{cQ_{00}}{K}$

Controls	H	
G	1	0
2	$\frac{(1-a)Q_{21}}{(1-K)}$	$\frac{(1-a)Q_{20}}{(1-K)}$
0	$\frac{(1-c)Q_{01}}{(1-K)}$	$\frac{(1-c)Q_{00}}{(1-K)}$

Middle row and middle column deleted:

Cases	H	
G	2	0
2	$\frac{aQ_{22}}{K}$	$\frac{aQ_{20}}{K}$
0	$\frac{cQ_{02}}{K}$	$\frac{cQ_{00}}{K}$

Controls	H	
G	2	0
2	$\frac{(1-a)Q_{22}}{(1-K)}$	$\frac{(1-a)Q_{20}}{(1-K)}$
0	$\frac{(1-c)Q_{02}}{(1-K)}$	$\frac{(1-c)Q_{00}}{(1-K)}$

When estimating the odds ratio for one of the four top left cells of the 3×3 table, relative to the bottom right cell, we make use of one of these pairs of 2×2 tables. Thus the desirable property (corresponding to the fact that, in the presence of a main effect, the odds ratio i_{jk} should be identical when calculated within a sample of cases as when calculated within a sample of controls) is inherited directly from the 2×2 table situation described earlier.

Consider now the presence of main effects at both loci:

$$f_{jk} = \frac{\exp(\alpha + \beta_1 I(j=1) + \beta_2 I(j=2) + \gamma_1 I(k=1) + \gamma_2 I(k=2))}{1+\exp(\alpha + \beta_1 I(j=1) + \beta_2 I(j=2) + \gamma_1 I(k=1) + \gamma_2 I(k=2))}$$

(where f_{jk} represents the penetrance associated with possessing j copies of the G_1 allele and k copies of the H_1 allele). As in the 2×2 table situation, in the presence of main effects at both loci, the desired cancelling of terms when calculating odds ratios no longer occurs. However, if we make a rare disease

assumption, we may assume that

$$f_{jk} \approx e^{\alpha+\beta_1 I(j=1)+\beta_2 I(j=2)+\gamma_1 I(k=1)+\gamma_2 I(k=2)}$$

Writing $A = e^{\alpha}$, $B_j = e^{\beta_j}$ and $C_k = e^{\gamma_k}$, we may write the genotype distributions in case and control populations as follows:

Cases	H		
G	2	1	0
2	$\frac{AB_2C_2Q_{22}}{K}$	$\frac{AB_2C_1Q_{21}}{K}$	$\frac{AB_2Q_{20}}{K}$
1	$\frac{AB_1C_2Q_{12}}{K}$	$\frac{AB_1C_1Q_{11}}{K}$	$\frac{AB_1Q_{10}}{K}$
0	$\frac{AC_2Q_{02}}{K}$	$\frac{AC_1Q_{01}}{K}$	$\frac{AQ_{00}}{K}$

Controls	H		
G	2	1	0
2	Q_{22}	Q_{21}	Q_{20}
1	Q_{12}	Q_{11}	Q_{10}
0	Q_{02}	Q_{01}	Q_{00}

(since, under the rare disease assumption, the distribution in controls is the same as in the general population). When calculating odds ratios based on deleting rows and columns from each of the above tables, the A, B_j, C_k terms cancel in the same way as in 2×2 table situation. Thus, for a rare disease, even when there are main effects at both loci, the odds ratio i_{jk} should be identical when calculated within the sample of cases as when calculated within the sample of controls.

PLINK's *fast-epistasis* statistic

Consider the odds ratio employed in PLINK's *fast-epistasis* statistic [1]. In the presence of main effects at a single locus, using the same notation as above, the odds ratio calculated for cases is

$$\mathrm{OR}_{\mathrm{FE},A} = \left(\frac{aQ_{22} + \frac{aQ_{21}+bQ_{12}}{2} + \frac{bQ_{11}}{4}}{cQ_{02} + \frac{cQ_{01}+bQ_{12}}{2} + \frac{bQ_{11}}{4}} \right) \times \left(\frac{cQ_{00} + \frac{cQ_{10}+bQ_{01}}{2} + \frac{bQ_{11}}{4}}{aQ_{20} + \frac{aQ_{21}+bQ_{10}}{2} + \frac{bQ_{11}}{4}} \right)$$

while that for controls is obtained by replacing a, b and c by $1-a, 1-b$ and $1-c$ in the above equation. These two quantities are not in general identical. However, for general population under HWE, we have

$$\begin{aligned}
\mathrm{OR}_{\mathrm{FE},A} &= \left(\frac{a\psi_{11}^2 + a\psi_{11}\psi_{12} + b\psi_{21}\psi_{11} + b\frac{\psi_{11}\psi_{22}+\psi_{12}\psi_{21}}{2}}{c\psi_{21}^2 + c\psi_{21}\psi_{22} + b\psi_{21}\psi_{11} + b\frac{\psi_{11}\psi_{22}+\psi_{12}\psi_{21}}{2}} \right) \times \left(\frac{c\psi_{22}^2 + c\psi_{21}\psi_{22} + b\psi_{12}\psi_{22} + b\frac{\psi_{11}\psi_{22}+\psi_{12}\psi_{21}}{2}}{a\psi_{12}^2 + a\psi_{12}\psi_{11} + b\psi_{12}\psi_{22} + b\frac{\psi_{11}\psi_{22}+\psi_{12}\psi_{21}}{2}} \right) \\
&= e^{\lambda_\psi} \left(\frac{a(\psi_{11}+\psi_{12}) + b(\psi_{21}+\psi_{22}) - b\frac{D_\psi}{2\psi_{11}}}{c(\psi_{21}+\psi_{22}) + b(\psi_{11}+\psi_{12}) + b\frac{D_\psi}{2\psi_{21}}} \right) \times \left(\frac{c(\psi_{22}+\psi_{21}) + b(\psi_{12}+\psi_{11}) - b\frac{D_\psi}{2\psi_{22}}}{a(\psi_{12}+\psi_{11}) + b(\psi_{22}+\psi_{21}) + b\frac{D_\psi}{2\psi_{12}}} \right),
\end{aligned}$$

where λ_ψ and D_ψ relate to the log odds ratio and linkage disequilibrium parameters $\lambda(\theta)$ and D (see Text S1 and Equation (3) in main manuscript) calculated with respect to the general population i.e.

$$\lambda_\psi = \log \frac{\psi_{11}\psi_{22}}{\psi_{12}\psi_{21}}$$

and

$$D_\psi = \psi_{11} - (\psi_{11} + \psi_{12})(\psi_{11} + \psi_{21}) = \psi_{11}\psi_{22} - \psi_{12}\psi_{21}$$

where ψ_{jk} is the haplotype frequency of haplotype G_j-H_k. The expression for $\mathrm{OR}_{\mathrm{FE},A}$ reduces to 1 if $\lambda_\psi = 0$ and $D_\psi = 0$. By a similar argument, the expression for the odds ratio in controls $\mathrm{OR}_{\mathrm{FE},N}$ reduces to 1 if $\lambda_\psi = 0$ and $D_\psi = 0$. Therefore, in the presence of a main effect at a single locus, the *fast-epistasis* statistic possesses the invariant property, provided the two loci are not in LD.

Now consider the situation where there are main effects at both loci, but the disease is rare. Using the same notation as for the joint effects statistic above, the odds ratio calculated for cases is

$$\begin{aligned}\mathrm{OR}_{\mathrm{FE},A} \quad &= \quad \left(\frac{4AB_2C_2Q_{22}+2AB_2C_1Q_{21}+2AB_1C_2Q_{12}+AB_1C_1Q_{11}}{4AB_2Q_{20}+2AB_2C_1Q_{21}+2AB_1Q_{10}+AB_1C_1Q_{11}}\right) \\ &\qquad \times\left(\frac{4AQ_{00}+2AC_1Q_{01}+2AB_1Q_{10}+AB_1C_1Q_{11}}{4AC_2Q_{02}+2AC_1Q_{01}+2AB_1C_2Q_{12}+AB_1C_1Q_{11}}\right)\end{aligned}$$

while that for controls is obtained by replacing terms involving A, AB_j, AC_k and AB_jC_k with 1 in the above equation. Simplifying, we have

$$\mathrm{OR}_{\mathrm{FE},A} = \left(\frac{4B_2C_2Q_{22}+2B_2C_1Q_{21}+2B_1C_2Q_{12}+B_1C_1Q_{11}}{4B_2Q_{20}+2B_2C_1Q_{21}+2B_1Q_{10}+B_1C_1Q_{11}}\right)\times\left(\frac{4Q_{00}+2C_1Q_{01}+2B_1Q_{10}+B_1C_1Q_{11}}{4C_2Q_{02}+2C_1Q_{01}+2B_1C_2Q_{12}+B_1C_1Q_{11}}\right)$$

and

$$\mathrm{OR}_{\mathrm{FE},N} = \left(\frac{4Q_{22}+2Q_{21}+2Q_{12}+Q_{11}}{4Q_{20}+2Q_{21}+2Q_{10}+Q_{11}}\right) \times \left(\frac{4Q_{00}+2Q_{01}+2Q_{10}+Q_{11}}{4Q_{02}+2Q_{01}+2Q_{12}+Q_{11}}\right)$$

These two odds ratios $\mathrm{OR}_{\mathrm{FE},A}$ and $\mathrm{OR}_{\mathrm{FE},N}$ are not in general identical. If we assume a general population in HWE, we have

$$
\begin{aligned}
\mathrm{OR}_{\mathrm{FE},A} &= \left(\frac{4B_2C_2\psi_{11}^2+4B_2C_1\psi_{11}\psi_{12}+4B_1C_2\psi_{11}\psi_{21}+2B_1C_1(\psi_{11}\psi_{22}+\psi_{12}\psi_{21})}{4B_2\psi_{12}^2+4B_2C_1\psi_{11}\psi_{12}+4B_1\psi_{12}\psi_{22}+2B_1C_1(\psi_{11}\psi_{22}+\psi_{12}\psi_{21})}\right) \\
&\quad \times \left(\frac{4\psi_{22}^2+4C_1\psi_{22}\psi_{21}+4B_1\psi_{12}\psi_{22}+2B_1C_1(\psi_{11}\psi_{22}+\psi_{12}\psi_{21})}{4C_2\psi_{21}^2+4C_1\psi_{21}\psi_{22}+4B_1C_2\psi_{11}\psi_{21}+2B_1C_1(\psi_{11}\psi_{22}+\psi_{12}\psi_{21})}\right) \\
&= \frac{\psi_{11}\psi_{22}}{\psi_{12}\psi_{21}}\left(\frac{4B_2(C_2\psi_{11}+C_1\psi_{12})+4B_1(C_2\psi_{21}+C_1\psi_{22})+2B_1C_1(\frac{\psi_{12}\psi_{21}}{\psi_{11}}-\psi_{22})}{4B_2(\psi_{12}+C_1\psi_{11})+4B_1(\psi_{22}+C_1\psi_{21})+2B_1C_1(\frac{\psi_{11}\psi_{22}}{\psi_{12}}-\psi_{21})}\right) \\
&\quad \times \left(\frac{4(\psi_{22}+C_1\psi_{21})+4B_1(\psi_{12}+C_1\psi_{11})+2B_1C_1(\frac{\psi_{12}\psi_{21}}{\psi_{22}}-\psi_{11})}{4(C_2\psi_{21}+C_1\psi_{22})+4B_1(C_2\psi_{11}+C_1\psi_{12})+2B_1C_1(\frac{\psi_{11}\psi_{21}}{\psi_{12}}-\psi_{12})}\right) \\
&= e^{\lambda_\psi}\left(\frac{4B_2(C_2\psi_{11}+C_1\psi_{12})+4B_1(C_2\psi_{21}+C_1\psi_{22})-2B_1C_1\frac{D_\psi}{\psi_{11}}}{4B_2(\psi_{12}+C_1\psi_{11})+4B_1(\psi_{22}+C_1\psi_{21})+2B_1C_1\frac{D_\psi}{\psi_{12}}}\right) \\
&\quad \times \left(\frac{4(\psi_{22}+C_1\psi_{21})+4B_1(\psi_{12}+C_1\psi_{11})-2B_1C_1\frac{D_\psi}{\psi_{22}}}{4(C_2\psi_{21}+C_1\psi_{22})+4B_1(C_2\psi_{11}+C_1\psi_{12})+2B_1C_1\frac{D_\psi}{\psi_{21}}}\right)
\end{aligned}
$$

If $\lambda_\psi = 0$ and $D_\psi = 0$, this expression reduces to:

$$
\mathrm{OR}_{\mathrm{FE},A} = \left(\frac{B_2(C_2\psi_{11}+C_1\psi_{12})+B_1(C_2\psi_{21}+C_1\psi_{22})}{B_2(\psi_{12}+C_1\psi_{11})+B_1(\psi_{22}+C_1\psi_{21})}\right) \times \left(\frac{(\psi_{22}+C_1\psi_{21})+B_1(\psi_{12}+C_1\psi_{11})}{(C_2\psi_{21}+C_1\psi_{22})+B_1(C_2\psi_{11}+C_1\psi_{12})}\right)
$$

Although this is not in general equal to $\mathrm{OR}_{\mathrm{FE},N}$ (which equals 1 when $\lambda_\psi = 0$ and $D_\psi = 0$), for specific choices of B_1, B_2, C_1, C_2, these odds ratios may be equal. In particular, for the choice of values used in our simulation Scenario 5c ($B_1 = C_1 = 3$; $B_2 = C_2 = 9$) we find that $\mathrm{OR}_{\mathrm{FE},A} = \frac{3}{1} \times \frac{1}{3} = 1 = \mathrm{OR}_{\mathrm{FE},N}$. Thus, assuming a rare disease and no population-level LD, the *fast-epistasis* method does possess the invariant property under this particular choice of simulation parameters, which explains why the type 1 error is correct for the adjusted *fast-epistasis* method in simulation Scenario 5c (when there is no population-level LD). More generally, if we assume a multiplicative model for the effects of alleles at both loci (i.e. $B_1 = B$, $C_1 = C$, $B_2 = B^2$, $C_2 = C^2$, for some parameters B and C), which is equivalent to an additive model on the log odds scale, then we find $\mathrm{OR}_{\mathrm{FE},A} = 1 = \mathrm{OR}_{\mathrm{FE},N}$. Alternatively, if you assume a recessive model (i.e. $B_1 = 1$, $C_1 = 1$), and also assume no population level LD (so $\lambda_\psi = D_\psi = 0$), then the log odds ratio in cases again reduces to 0, as required. This explains why the type 1 error is also correct for the adjusted *fast-epistasis* method in simulation Scenario 5d (when there is no population-level LD). Therefore, assuming a rare disease and no population-level LD, the adjusted *fast-epistasis* method does indeed possess the invariant property (and thus will be valid in the presence of main effects) when

alleles at both loci act either additively or recessively on the log odds scale.

Wellek and Ziegler (2009) correlation coefficient

Consider application of the Wellek and Ziegler's [2] correlation coefficient. We use the same notation as for the joint effects statistic above. For a general population under HWE, using the parameterisation $\psi_{11} = pu + D_\psi$, $\psi_{12} = pv - D_\psi$, $\psi_{21} = qu - D_\psi$ and $\psi_{22} = qv + D_\psi$ where $q = 1 - p$ and $v = 1 - u$, the genotype frequencies Q_{ij} may be represented as a quadratic function in D_ψ:

$$\begin{pmatrix} Q_{22} & Q_{21} & Q_{20} \\ Q_{12} & Q_{11} & Q_{10} \\ Q_{02} & Q_{01} & Q_{00} \end{pmatrix}$$
$$= \begin{pmatrix} (pu + D_\psi)^2 & 2(pu + D_\psi)(pv - D_\psi) & (pv - D_\psi)^2 \\ 2(pu + D_\psi)(qu - D_\psi) & 2(pu + D_\psi)(qv + D_\psi) + 2(pv - D_\psi)(qu - D_\psi) & 2(pv - D_\psi)(qv + D_\psi) \\ (qu - D_\psi)^2 & 2(qu - D_\psi)(qv + D_\psi) & (qv + D_\psi)^2 \end{pmatrix}$$
$$= gh^T + 2D_\psi a_g a_h^T + D_\psi^2 ee^T,$$

where $g = (p^2, 2pq, q^2)^T$, $h = (u^2, 2uv, v^2)^T$, $a_g = (p, q-p, -q)^T$, $a_h = (u, v-u, -v)^T$ and $e = (1, -2, 1)^T$. Consequently, Q_{ij} can be written as $g_i h_j + 2D_\psi a_{g,i} a_{h,j} + D_\psi^2 e_i e_j$.

If we apply the above formula for Q_{ij} to the genotype frequencies in the case population that are represented by the left hand table on page 6 of this Text S2, we obtain:

$$Q_{A,ij} = AB_i C_j (g_i h_j + 2D_\psi a_{g,i} a_{h,j} + D_\psi^2 e_i e_j)/K, \tag{1}$$

where we defined $B_0 = C_0 = 1$ as a matter of convenience. Since $K = \sum_{i,j} AB_i C_j (g_i h_j + 2D_\psi a_{g,i} a_{h,j} + D_\psi^2 e_i e_j)$, we have

$$K = A\left\{(\sum_i B_i g_i)(\sum_j C_j h_j) + 2D_\psi(\sum_i B_i a_{g,i})(\sum_j C_j a_{h,j}) + D_\psi^2(\sum_i B_i e_i)(\sum_j C_j e_j)\right\}.$$

If we define $d = (2, 1, 0)^T$, the Wellek and Ziegler correlation coefficient for cases is written as

$$R_{\mathrm{WZ},A} = \frac{\sum_{ij} d_i d_j Q_{A,ij} - (\sum_{ij} d_i Q_{A,ij})(\sum_{ij} d_j Q_{A,ij})}{\sqrt{\sum_{ij} d_i^2 Q_{A,ij} - (\sum_{ij} d_i Q_{A,ij})^2}\sqrt{\sum_{ij} d_j^2 Q_{A,ij} - (\sum_{ij} d_j Q_{A,ij})^2}}.$$

Applying the above formula to the numerator of $R_{\mathrm{WZ},A}$, we have that

$$\begin{aligned}
&\sum_{ij} d_i d_j Q_{A,ij} - (\sum_{ij} d_i Q_{A,ij})(\sum_{ij} d_j Q_{A,ij}) \\
&= \frac{(\sum_i d_i B_i g_i)(\sum_j d_j C_j h_j) + 2D_\psi(\sum_i d_i B_i a_{g,i})(\sum_j d_j C_j a_{h,j}) + D_\psi^2(\sum_i d_i B_i e_i)(\sum_j d_j C_j e_j)}{K/A} \\
&\quad - \frac{(\sum_i d_i B_i g_i)(\sum_j C_j h_j) + 2D_\psi(\sum_i d_i B_i a_{g,i})(\sum_j C_j a_{h,j}) + D_\psi^2(\sum_i d_i B_i e_i)(\sum_j C_j e_j)}{K/A} \\
&\quad \times \frac{(\sum_i B_i g_i)(\sum_j d_j C_j h_j) + 2D_\psi(\sum_i B_i a_{g,i})(\sum_j d_j C_j a_{h,j}) + D_\psi^2(\sum_i B_i e_i)(\sum_j d_j C_j e_j)}{(\sum_i B_i g_i)(\sum_j C_j h_j) + 2D_\psi(\sum_i B_i a_{g,i})(\sum_j C_j a_{h,j}) + D_\psi^2(\sum_i B_i e_i)(\sum_j C_j e_j)}. \qquad (2)
\end{aligned}$$

The parameterisation represented by the left hand table on page 6 of this Text S2 corrresponds to modelling either main effects at both loci, under a rare disease assumption, or main effects at a single locus, without making any rare disease assumption. We consider each of these possibilities in turn. First, we consider the quantity (2) under the situation where a single main effect at locus G is present. This corresponds to reparameterising $A = c, B_2 = a/c, B_1 = b/c$ and $C_2 = C_1 = 1$, so that the left hand table on page 6 becomes equivalent to the left hand table in the middle of page 4 of this Text S2. By noting that $\sum_i g_i = \sum_j h_j = 1$ and $\sum_i a_{g,i} = \sum_j a_{h,j} = \sum_i e_i = 0$, Equation (2) is simplified to

$$\begin{aligned}
&\frac{(\sum_i d_i B_i g_i)(2u) + 2D_\psi(\sum_i d_i B_i a_{g,i})}{K/A} - \frac{(\sum_i d_i B_i g_i)}{K/A} \times \frac{(\sum_i B_i g_i)(2u) + 2D_\psi(\sum_i B_i a_{g,i})}{(\sum_i B_i g_i)} \\
&= 2D_\psi \frac{(\sum_i d_i B_i a_{g,i}) - \frac{\sum_i B_i a_{g,i}}{\sum_i B_i g_i}}{\sum_i B_i g_i} \\
&= 2D_\psi \frac{\{2\frac{a}{c}p + \frac{b}{c}(q-p)\} - \frac{\frac{a}{c}p + 2\frac{b}{c}(q-p) - q}{\frac{a}{c}p^2 + 2\frac{b}{c}pq + q^2}}{\frac{a}{c}p^2 + 2\frac{b}{c}pq + q^2}. \qquad (3)
\end{aligned}$$

This quantity reduces to $2D_\psi$ if $a = b = c$, i.e. no main effects, which coincides with Equation (4) of [2].

Similary, the first term of the denominator of $R_{\mathrm{WZ},A}$ is expressed as

$$\sqrt{\sum_{ij} d_i^2 Q_{A,ij} - (\sum_{ij} d_i Q_{A,ij})^2} = \sqrt{\sum_i d_i^2 B_i g_i - \frac{(\sum_{ij} d_i B_i g_i)^2}{K/A}} \Big/ \sqrt{K/A}$$
$$= \sqrt{(4\frac{a}{c}p^2 + 2\frac{b}{c}pq) - \frac{(2\frac{a}{c}p^2 + 2\frac{b}{c}pq)^2}{\frac{a}{c}p^2 + 2\frac{b}{c}pq + q^2}} \Big/ \sqrt{\frac{a}{c}p^2 + 2\frac{b}{c}pq + q^2},$$

which reduces to $\sqrt{2pq}$ if $a = b = c$. For the second term of the denominator of $R_{\mathrm{WZ},A}$,

$$\sqrt{\sum_{ij} d_j^2 Q_{A,ij} - (\sum_{ij} d_j Q_{A,ij})^2}$$
$$= \Bigg[(\sum_i B_i g_i)(\sum_j d_j^2 h_j) + 2D_\psi(\sum_i B_i a_{g,i})(\sum_j d_j^2 a_{h,j}) + 2D_\psi^2(\sum_i B_i e_i)(\sum_j d_j^2 e_j)$$
$$- \frac{\{(\sum_i B_i g_i)(\sum_j d_j h_j) + 2D_\psi(\sum_i B_i a_{g,i})(\sum_j d_j a_{h,j}) + 2D_\psi^2(\sum_i B_i e_i)(\sum_j d_j e_j)\}^2}{K/A} \Bigg]^{1/2} \Big/ \sqrt{K/A}$$
$$= \frac{\left[(\sum_i B_i g_i)(2u)(2u+v) + 2D_\psi(\sum_i B_i a_{g,i})(2u+1) + 4D_\psi^2(\sum_i B_i e_i) - \frac{\{(\sum_i B_i g_i)(2u) + 2D_\psi(\sum_i B_i a_{g,i})\}^2}{\frac{a}{c}p^2 + 2\frac{b}{c}pq + q^2} \right]^{1/2}}{\sqrt{\frac{a}{c}p^2 + 2\frac{b}{c}pq + q^2}}.$$

When $a = b = c$, the last display reduces to $\sqrt{2uv}$ because $\sum_i B_i g_i = 1$ and $\sum_i B_i a_{g,i} = 0$. These results generalize those obtained by [2] and show that the Wellek and Ziegler correlation coefficient reduces to $D_\psi/\sqrt{pquv}$ (i.e. Pearson's correlation coefficient based on haplotypes) if $a = b = c$.

The three quantities above and $R_{\mathrm{WZ},A}$ may vary depending on the choice of a and b. Note that, when a main effect is present at locus G, the corresponding quantities for the control population are obtained by setting $A = 1 - c, B_2 = (1-a)/(1-c), B_1 = (1-b)/(1-c)$ and $C_2 = C_1 = 1$. Thus, Wellek and Ziegler correlation coefficients R_{WZ} calculated for case and control populations are in general not identical. In other words, the Wellek and Ziegler correlation coefficient does not possess the invariant property. However, if $D_\psi = 0$, Equation (3) becomes zero for any choice of a and b. Therefore, the Wellek and Ziegler correlation coefficient does possess the invariant property, provided the two loci are not in LD.

The above argument can be extended to the presence of main effects at both loci, provided we make a rare disease assumption. It can be seen that if $D_\psi = 0$ the quantity (2) reduces to zero for arbitrary choice of B_2, B_1, C_2, C_1. Because the denominator of R_{WZ} is positive, $R_{\mathrm{WZ},A} = 0$. This is also understood from

the fact that if $D_\psi = 0$ the genotype frequencies are expressed as

$$Q_{A,ij} = A(B_i g_i)(C_j h_j)/K,$$

which implies the statistical independence between marginal distributions of each locus and the correlation coefficient should be zero. Under a rare disease assumption, the Wellek and Ziegler correlation coefficient for the control population is zero. Consequently, even with main effects at both loci, the Wellek and Ziegler statistic possesses the invariant property assuming a rare disease and no population-level LD.

Wu et al. (2010) statistic

The Wu et al. (2010) [3] odds ratio is based on estimated haplotype frequencies, estimated under the (potentially incorrect) assumption that the haplotypes come together independently (i.e. are in HWE) in cases. This quantity is not tractable because HWE does not necessarily hold in cases under the presence of main effects at either locus [4]. There is, therefore, in general, no guarrantee that the invariant property is satisfied, although it is possible that under certain specific genetic models it may hold. We refer the reader to our simulation study for evaluation of the properties of the Wu et al. (2010) odds ratio in various situations.

Ideal Wu statistic

The ideal Wu statistic is calculatable if phase information is available. Consider the possible configurations of phased diplotypes (combinations of haplotypes) that an individual can possess at two diallelic loci, G and H, with locus G having alleles G_1 and G_2 and locus H having alleles H_1 and H_2. In Text S3, we show that, assuming no parent-of-origin effects, the diplotype probabilities in cases and controls may be written:

Cases	Maternal haplotype			
Paternal haplotype	G_1-H_1	G_1-H_2	G_2-H_1	G_2-H_2
G_1-H_1	$\psi_{11}^2 f_{1111}/K$	$\psi_{11}\psi_{12} f_{1112}/K$	$\psi_{11}\psi_{21} f_{1121}/K$	$\psi_{11}\psi_{22} f_{1122}/K$
G_1-H_2	$\psi_{12}\psi_{11} f_{1211}/K$	$\psi_{12}^2 f_{1212}/K$	$\psi_{12}\psi_{21} f_{1221}/K$	$\psi_{12}\psi_{22} f_{1222}/K$
G_2-H_1	$\psi_{21}\psi_{11} f_{2111}/K$	$\psi_{21}\psi_{12} f_{2112}/K$	$\psi_{21}^2 f_{2121}/K$	$\psi_{21}\psi_{22} f_{2122}/K$
G_2-H_2	$\psi_{22}\psi_{11} f_{2211}/K$	$\psi_{22}\psi_{12} f_{2212}/K$	$\psi_{22}\psi_{21} f_{2221}/K$	$\psi_{22}^2 f_{2222}/K$

Controls	Maternal haplotype			
Paternal haplotype	G_1-H_1	G_1-H_2	G_2-H_1	G_2-H_2
G_1-H_1	$\frac{\psi_{11}^2(1-f_{1111})}{(1-K)}$	$\frac{\psi_{11}\psi_{12}(1-f_{1112})}{(1-K)}$	$\frac{\psi_{11}\psi_{21}(1-f_{1121})}{(1-K)}$	$\frac{\psi_{11}\psi_{22}(1-f_{1122})}{(1-K)}$
G_1-H_2	$\frac{\psi_{12}\psi_{11}(1-f_{1211})}{(1-K)}$	$\frac{\psi_{12}^2(1-f_{1212})}{(1-K)}$	$\frac{\psi_{12}\psi_{21}(1-f_{1221})}{(1-K)}$	$\frac{\psi_{12}\psi_{22}(1-f_{1222})}{(1-K)}$
G_2-H_1	$\frac{\psi_{21}\psi_{11}(1-f_{2111})}{(1-K)}$	$\frac{\psi_{21}\psi_{12}(1-f_{2112})}{(1-K)}$	$\frac{\psi_{21}^2(1-f_{2121})}{(1-K)}$	$\frac{\psi_{21}\psi_{22}(1-f_{2122})}{(1-K)}$
G_2-H_2	$\frac{\psi_{22}\psi_{11}(1-f_{2211})}{(1-K)}$	$\frac{\psi_{22}\psi_{12}(1-f_{2212})}{(1-K)}$	$\frac{\psi_{22}\psi_{21}(1-f_{2221})}{(1-K)}$	$\frac{\psi_{22}^2(1-f_{2222})}{(1-K)}$

where ψ_{jk} is the population haplotype frequency of haplotype G_j-H_k, f_{jklm} is the probability of being diseased for an individual with diplotype G_j-H_k/G_l-H_m and K is the population prevalance. Suppose main effects exist at locus G, but not at locus H. Then we may write $f_{1k1m} = a$, $f_{1k2m} = f_{2k1m} = b$ and $f_{2k2m} = c$. The diplotype probabilities in cases and controls may be written:

Cases	Maternal haplotype			
Paternal haplotype	G_1-H_1	G_1-H_2	G_2-H_1	G_2-H_2
G_1-H_1	$\frac{\psi_{11}^2 a}{K}$	$\frac{\psi_{11}\psi_{12}a}{K}$	$\frac{\psi_{11}\psi_{21}b}{K}$	$\frac{\psi_{11}\psi_{22}b}{K}$
G_1-H_2	$\frac{\psi_{12}\psi_{11}a}{K}$	$\frac{\psi_{12}^2 a}{K}$	$\frac{\psi_{12}\psi_{21}b}{K}$	$\frac{\psi_{12}\psi_{22}b}{K}$
G_2-H_1	$\frac{\psi_{21}\psi_{11}b}{K}$	$\frac{\psi_{21}\psi_{12}b}{K}$	$\frac{\psi_{21}^2 c}{K}$	$\frac{\psi_{21}\psi_{22}c}{K}$
G_2-H_2	$\frac{\psi_{22}\psi_{11}b}{K}$	$\frac{\psi_{22}\psi_{12}b}{K}$	$\frac{\psi_{22}\psi_{21}c}{K}$	$\frac{\psi_{22}^2 c}{K}$

Controls	Maternal haplotype			
Paternal haplotype	G_1-H_1	G_1-H_2	G_2-H_1	G_2-H_2
G_1-H_1	$\frac{\psi_{11}^2(1-a)}{(1-K)}$	$\frac{\psi_{11}\psi_{12}(1-a)}{(1-K)}$	$\frac{\psi_{11}\psi_{21}(1-b)}{(1-K)}$	$\frac{\psi_{11}\psi_{22}(1-b)}{(1-K)}$
G_1-H_2	$\frac{\psi_{12}\psi_{11}(1-a)}{(1-K)}$	$\frac{\psi_{12}^2(1-a)}{(1-K)}$	$\frac{\psi_{12}\psi_{21}(1-b)}{(1-K)}$	$\frac{\psi_{12}\psi_{22}(1-b)}{(1-K)}$
G_2-H_1	$\frac{\psi_{21}\psi_{11}(1-b)}{(1-K)}$	$\frac{\psi_{21}\psi_{12}(1-b)}{(1-K)}$	$\frac{\psi_{21}^2(1-c)}{(1-K)}$	$\frac{\psi_{21}\psi_{22}(1-c)}{(1-K)}$
G_2-H_2	$\frac{\psi_{22}\psi_{11}(1-b)}{(1-K)}$	$\frac{\psi_{22}\psi_{12}(1-b)}{(1-K)}$	$\frac{\psi_{22}\psi_{21}(1-c)}{(1-K)}$	$\frac{\psi_{22}^2(1-c)}{(1-K)}$

The ideal Wu et al. statistic (Equation (4) in their paper) is calculated through counting haplotypes as observed in the cells of the above two tables. Each cell contributes two haplotypes that then contribute to the counts in the relevant haplotype categories. This results in the ideal Wu statistic corresponding

to an estimate of the following log odds ratio quantity in cases:

$$\begin{aligned}
& \log \frac{(2a\psi_{11}^2 + 2a\psi_{11}\psi_{12} + 2b\psi_{11}\psi_{21}2b\psi_{11}\psi_{22})(2b\psi_{22}\psi_{11} + 2b\psi_{22}\psi_{12} + 2c\psi_{22}\psi_{21} + 2c\psi_{22}^2)}{(2a\psi_{12}\psi_{11} + 2a\psi_{12}^2 + 2b\psi_{12}\psi_{21} + 2b\psi_{12}\psi_{22})(2b\psi_{21}\psi_{11} + 2b\psi_{21}\psi_{12} + 2c\psi_{21}^2 + 2c\psi_{21}\psi_{22})} \\
= & \log \frac{2\psi_{11}[a\psi_{11} + a\psi_{12} + b\psi_{21} + b\psi_{22}]2\psi_{22}[b\psi_{11} + b\psi_{12} + c\psi_{21} + c\psi_{22}]}{2\psi_{12}[a\psi_{11} + a\psi_{12} + b\psi_{21} + b\psi_{22}]2\psi_{21}[b\psi_{11} + b\psi_{12} + c\psi_{21} + c\psi_{22}]} \\
= & \log \frac{\psi_{11}\psi_{22}}{\psi_{12}\psi_{21}} \\
= & \lambda_\psi
\end{aligned}$$

while the log odds ratio in controls takes the same form, but with a, b, c, replaced by $(1-a)$, $(1-b)$, $(1-c)$ respectively. Since both these log odds ratio quantities reduce to λ_ψ, we find that the odds ratio calculated separately within case and control samples is invariant even when one locus has a main effect. Thus, the ideal Wu case/control approach is valid in the presence of main effects at a single locus. Furthermore, provided there is no population-level LD, $\lambda_\psi = 0$, and so the ideal Wu case-only approach is also valid in the presence of main effects at a single locus.

Now consider main effects at both loci, so that

$$f_{jklm} = \frac{e^{\alpha+\beta_1 I(j+l=3)+\beta_2 I(j+l=2)+\gamma_1 I(k+m=3)+\gamma_2 I(k+m=2)}}{1+e^{\alpha+\beta_1 I(j+l=3)+\beta_2 I(j+l=2)+\gamma_1 I(k+m=3)+\gamma_2 I(k+m=2)}}$$

Under a rare disease assumption, we may write

$$f_{jklm} \approx e^{\alpha+\beta_1 I(j+l=3)+\beta_2 I(j+l=2)+\gamma_1 I(k+m=3)+\gamma_2 I(k+m=2)}$$

and we define $A = e^\alpha$, $B_1 = e^{\beta_1}$, $B_2 = e^{\beta_2}$, $C_1 = e^{\gamma_1}$, $C_2 = e^{\gamma_2}$. Then the diplotype probabilities in cases and controls are:

Cases	Maternal haplotype			
Paternal haplotype	G_1-H_1	G_1-H_2	G_2-H_1	G_2-H_2
G_1-H_1	$\frac{\psi_{11}^2 AB_2C_2}{K}$	$\frac{\psi_{11}\psi_{12}AB_2C_1}{K}$	$\frac{\psi_{11}\psi_{21}AB_1C_2}{K}$	$\frac{\psi_{11}\psi_{22}AB_1C_1}{K}$
G_1-H_2	$\frac{\psi_{12}\psi_{11}AB_2C_1}{K}$	$\frac{\psi_{12}^2 AB_2}{K}$	$\frac{\psi_{12}\psi_{21}AB_1C_1}{K}$	$\frac{\psi_{12}\psi_{22}AB_1}{K}$
G_2-H_1	$\frac{\psi_{21}\psi_{11}AB_1C_2}{K}$	$\frac{\psi_{21}\psi_{12}AB_1C_1}{K}$	$\frac{\psi_{21}^2 AC_2}{K}$	$\frac{\psi_{21}\psi_{22}AC_1}{K}$
G_2-H_2	$\frac{\psi_{22}\psi_{11}AB_1C_1}{K}$	$\frac{\psi_{22}\psi_{12}AB_1}{K}$	$\frac{\psi_{22}\psi_{21}AC_1}{K}$	$\frac{\psi_{22}^2 A}{K}$

Controls	Maternal haplotype			
Paternal haplotype	G_1-H_1	G_1-H_2	G_2-H_1	G_2-H_2
G_1-H_1	ψ_{11}^2	$\psi_{11}\psi_{12}$	$\psi_{11}\psi_{21}$	$\psi_{11}\psi_{22}$
G_1-H_2	$\psi_{12}\psi_{11}$	ψ_{12}^2	$\psi_{12}\psi_{21}$	$\psi_{12}\psi_{22}$
G_2-H_1	$\psi_{21}\psi_{11}$	$\psi_{21}\psi_{12}$	ψ_{21}^2	$\psi_{21}\psi_{22}$
G_2-H_2	$\psi_{22}\psi_{11}$	$\psi_{22}\psi_{12}$	$\psi_{22}\psi_{21}$	ψ_{22}^2

(since, under a rare disease assumption, controls have the same diplotype probabilities as the general population)

The ideal Wu et al. statistic is again calculated through counting haplotypes as observed in the cells of each of the above two tables. In controls, this calculation results in an estimate of the following log odds ratio:

$$\begin{aligned}
& \log\frac{(2\psi_{11}^2+2\psi_{11}\psi_{12}+2\psi_{11}\psi_{21}+2\psi_{11}\psi_{22})(2\psi_{22}\psi_{11}+2\psi_{22}\psi_{12}+2\psi_{22}\psi_{21}+2\psi_{22}^2)}{(2\psi_{12}\psi_{11}+2\psi_{12}^2+2\psi_{12}\psi_{21}+2\psi_{12}\psi_{22})(2\psi_{21}\psi_{11}+2\psi_{21}\psi_{12}+2\psi_{21}^2+2\psi_{21}\psi_{22})} \\
= & \log\frac{2\psi_{11}[\psi_{11}+\psi_{12}+\psi_{21}+\psi_{22}]2\psi_{22}[\psi_{11}+\psi_{12}+\psi_{21}+\psi_{22}]}{2\psi_{12}[\psi_{11}+\psi_{12}+\psi_{21}+\psi_{22}]2\psi_{21}[\psi_{11}+\psi_{12}+\psi_{21}+\psi_{22}]} \\
= & \log\frac{\psi_{11}\psi_{22}}{\psi_{12}\psi_{21}} \\
= & \lambda_\psi
\end{aligned}$$

In cases, the log odds ratio estimated is instead

$$\begin{aligned}
& \log\frac{(\psi_{11}^2AB_2C_2+\psi_{11}\psi_{12}AB_2C_1+\psi_{11}\psi_{21}AB_1C_2+\psi_{11}\psi_{22}AB_1C_1)(\psi_{22}\psi_{11}AB_1C_1+\psi_{22}\psi_{12}AB_1+\psi_{22}\psi_{21}AC_1+\psi_{22}^2A)}{(\psi_{12}\psi_{11}AB_2C_1+\psi_{12}^2AB_2+\psi_{12}\psi_{21}AB_1C_1+\psi_{12}\psi_{22}AB_1)(\psi_{21}\psi_{11}AB_1C_2+\psi_{21}\psi_{12}AB_1C_1+\psi_{21}^2AC_2+\psi_{21}\psi_{22}AC_1)} \\
= & \log\frac{\psi_{11}[\psi_{11}B_2C_2+\psi_{12}B_2C_1+\psi_{21}B_1C_2+\psi_{22}B_1C_1]\psi_{22}[\psi_{11}B_1C_1+\psi_{12}B_1+\psi_{21}C_1+\psi_{22}]}{\psi_{12}[\psi_{11}B_2C_1+\psi_{12}B_2+\psi_{21}B_1C_1+\psi_{22}B_1]\psi_{21}[\psi_{11}B_1C_2+\psi_{12}B_1C_1+\psi_{21}C_2+\psi_{22}C_1]}
\end{aligned}$$

It is not clear that this quantity is in general equal to λ_ψ, i.e. that the invariant property should hold, when main effects operate at both loci. However, for certain models, the log odds ratio estimated in cases does turn out to be equal to λ_ψ. In particular, if you assume a multiplicative model for the effects of alleles at both loci (i.e. $B_1 = B$, $C_1 = C$, $B_2 = B^2$, $C_2 = C^2$, for some parameters B and C), which is equivalent to an additive model on the log odds scale, then, following some algebra, we find that the

log odds ratio reduces to λ_ψ as required. Alternatively, if you assume a recessive model (i.e. $B_1 = 1$, $C_1 = 1$), and also assume no population level LD (so $\lambda_\psi = D_\psi = 0$), then the log odds ratio in cases also reduces to 0, as required. These observations partly explain the results seen in our simulations (Scenarios 5c and 5d). The theory presented here relies on a rare disease assumption. However, the results from simulation Scenarios 5a and 5b suggest that, under these particular models, the performance of the ideal Wu statistic is generally quite robust to the presence of main effects at both loci, even without invoking a rare disease assumption.

References

1. Purcell S, Neale B, Todd-Brown K, Thomas L, Ferreira MA, et al. (2007) PLINK: a tool set for whole-genome association and population-based linkage analyses. Am J Hum Genet 81: 559–575.

2. Wellek S, Ziegler A (2009) A genotype based approach to assessing the association between single nucleotide polymorphisms. Hum Hered 67: 128–139.

3. Wu X, Dong H, Luo L, Zhu Y, Peng G, et al. (2010) A novel statistic for genome-wide interaction analysis. PLoS Genet 6: e1001131.

4. Zaykin DV, Meng Z, Ehm MG (2006) Contrasting linkage-disequilibrium patterns between cases and controls as a novel association-mapping method. Am J Hum Genet 78: 737–746.
