# Supplementary material for: Improved Statistics for Genome-Wide Interaction Analysis
Source: PLoS Genet. 2012 Apr 5;8(4):e1002625. doi: 10.1371/journal.pgen.1002625 (PMC3320596; doi:10.1371/journal.pgen.1002625)
Supplement: Text S3 — A logistic regression view of the Wu et al. statistic. (PDF) [file pgen.1002625.s015.pdf]

Text S3

A logistic regression view of the Wu et al. statistic

Here we provide motivation for our joint effects statistic via a logistic regression view of the statistic proposed by Wu et al. [1].

Consider the possible configurations of phased diplotypes (combinations of haplotypes) that an individual can possess at two diallelic loci, G and H, with locus G having alleles G_1 and G_2 and locus H having alleles H_1 and H_2. The four possible haplotypes result in 16 possible diplotypes. In the general population, assuming HWE, the distribution of these diplotypes is as shown in the following table, where ψ_{jk} is the haplotype frequency of haplotype G_j-H_k:

Paternal	Maternal haplotype			
haplotype	G_1-H_1	G_1-H_2	G_2-H_1	G_2-H_2
G_1-H_1	ψ_{11}^2	$\psi_{11}\psi_{12}$	$\psi_{11}\psi_{21}$	$\psi_{11}\psi_{22}$
G_1-H_2	$\psi_{12}\psi_{11}$	ψ_{12}^2	$\psi_{12}\psi_{21}$	$\psi_{12}\psi_{22}$
G_2-H_1	$\psi_{21}\psi_{11}$	$\psi_{21}\psi_{12}$	ψ_{21}^2	$\psi_{21}\psi_{22}$
G_2-H_2	$\psi_{22}\psi_{11}$	$\psi_{22}\psi_{12}$	$\psi_{22}\psi_{21}$	ψ_{22}^2

In theory, one could imagine that these 16 configurations result in 16 different penetrance (or log odds of disease) values. However, if we assume that there are no parent-of-origin effects i.e. the penetrance of diplotype G_j-H_k/G_l-H_m equals that of G_l-H_m/G_j-H_k, then we have 10 different penetrance value categories corresponding to the 10 upper right cells in the above table, (with each of the 6 lower left cells taking the same penetrance value as their mirror image cell on the upper right). In this situation, the log odds of disease for the different diplotypes can be written as:

Paternal	Maternal haplotype			
haplotype	G_1-H_1	G_1-H_2	G_2-H_1	G_2-H_2
G_1-H_1	$\alpha+\beta_2+\gamma_2+\delta_{22}$	$\alpha+\beta_2+\gamma_1+\delta_{21}$	$\alpha+\beta_1+\gamma_2+\delta_{12}$	$\alpha+\beta_1+\gamma_1+\delta'_{11}$
G_1-H_2	$\alpha+\beta_2+\gamma_1+\delta_{21}$	$\alpha+\beta_2$	$\alpha+\beta_1+\gamma_1+\delta_{11}$	$\alpha+\beta_1$
G_2-H_1	$\alpha+\beta_1+\gamma_2+\delta_{12}$	$\alpha+\beta_1+\gamma_1+\delta_{11}$	$\alpha+\gamma_2$	$\alpha+\gamma_1$
G_2-H_2	$\alpha+\beta_1+\gamma_1+\delta'_{11}$	$\alpha+\beta_1$	$\alpha+\gamma_1$	α

Here we have parameterized the 10 log odds values in terms of 10 standard logistic regression parameters: a baseline effect (α), effects due to one or two copies of the susceptibility allele at locus G (β_1, β_2), at locus H (γ_1, γ_2) and five interaction parameters (δ'_{11}, δ_{11}, δ_{12}, δ_{21}, δ_{22}). Note that there are two different interaction parameters (δ'_{11} and δ_{11}) that could operate when an individual is heterozygous at both loci, which allows the penetrance for diplotype G_1-H_1/G_2-H_2 to differ from that for G_1-H_2/G_2-H_1 i.e. models the difference between so-called 'cis' and 'trans' effects. If one wishes to assume that these diplotypes have the same penetrance (i.e. $\delta'_{11} = \delta_{11}$) then one would obtain a model for the log odds that corresponds to the usual 9-parameter 'saturated' model for combinations of genotypes at the two loci:

	Locus H		
Locus G	H_1H_1	H_1H_2	H_2H_2
G_1G_1	$\alpha+\beta_2+\gamma_2+\delta_{22}$	$\alpha+\beta_2+\gamma_1+\delta_{21}$	$\alpha+\beta_2$
G_1G_2	$\alpha+\beta_1+\gamma_2+\delta_{12}$	$\alpha+\beta_1+\gamma_1+\delta_{11}$	$\alpha+\beta_1$
G_2G_2	$\alpha+\gamma_2$	$\alpha+\gamma_1$	α

This 9-parameter model is convenient as, in general, phase is not observed, and so we do not have any data with which to distinguish between the penetrances of G_1-H_1/G_2-H_2 and G_1-H_2/G_2-H_1. If phased diplotypes were, in fact, observed (i.e. we observed which of the 10 different diplotype categories each individual falls into), we could use case/control data to fit the full 10 parameter model. However, when phase is not observed, we observe only the 9 genotype categories above, and thus have a maximum of 9 estimable penetrance parameters. If we wished to allow a different parameterisation where δ'_{11} and δ_{11} were not equal, then we would have to make some kind of other parameter restrictions, in order to not exceed the maximum of 9 estimable parameters.

In the main text, we pointed out that the log odds ratio used in the method proposed by Wu et al. [1] can be seen to be analagous to the quantity used in case-only analysis [2] [3] [4], if the unit of analysis is defined to be a 'haplotype' (rather than an individual) and if binary variables x_1 and x_2 are defined as indicator variables for the two possible alleles at each locus on the haplotype. This suggests that the method of Wu et al. coresponds to testing the interaction parameter δ in a standard logistic regression model:

$$\log \frac{p}{1-p} = \alpha_0 + \beta x_1 + \gamma x_2 + \delta x_1 x_2$$

where p represents the probability that a haplotype is 'diseased' (i.e. comes from a case rather than from a control).

There are two problems with applying this logistic regression model in practice. One is that diplotypes (and thus haplotypes) are not observed (so the unit of analysis required for fitting this logistic regression model is not, in fact, available). The second is that, even if haplotypes were observed (in the form of diplotypes), it is not clear that the constituent haplotypes in a diplotype should be considered to have independent risks of being 'diseased'. As pointed out by Sasieni [5], alleles (or, analagously, haplotypes) do not get disease; people with particular genotypes (or, analagously, with particular diplotypes) do. However, Sasieni [5] pointed out certain conditions under which splitting up genotypes into their constituent alleles (or, analagously, diplotypes into their constituent haplotypes) and treating them as 'independent' does produce inference consistent with what would be obtained from the underlying genotype (or diplotype) based model: namely, when HWE holds in the control population and when, moreover, the homozygous odds ratio is the square of the heterozygous one.

Considering Wu et al.'s approach as an implementation of the 'haplotype-based' logistic regression model above, this model imposes a particular structure on the log odds for a haplotype being 'diseased', namely that haplotypes G_1-H_1, G_1-H_2, G_2-H_1, G_2-H_2, have log odds of being diseased of α_0, $\alpha_0+\gamma$, $\alpha_0+\beta$, $\alpha_0+\beta+\gamma+\delta$ respectively. Converting this to a diplotype-based model, while imposing the condition of the homozygous odds ratio being the square of the heterozygous one, would suggest that Wu et al.'s approach in fact corresponds to fitting the following model for the log odds of disease for the different diplotypes:

Paternal haplotype	Maternal haplotype G_1-H_1	G_1-H_2	G_2-H_1	G_2-H_2
G_1-H_1	$2\alpha_0+2\beta+2\gamma+2\delta$	$2\alpha_0+2\beta+\gamma+\delta$	$2\alpha_0+\beta+2\gamma+\delta$	$2\alpha_0+\beta+\gamma+\delta$
G_1-H_2	$2\alpha_0+2\beta+\gamma+\delta$	$2\alpha_0+2\beta$	$2\alpha_0+\beta+\gamma$	$2\alpha_0+\beta$
G_2-H_1	$2\alpha_0+\beta+2\gamma+\delta$	$2\alpha_0+\beta+\gamma$	$2\alpha_0+2\gamma$	$2\alpha_0+\gamma$
G_2-H_2	$2\alpha_0+\beta+\gamma+\delta$	$2\alpha_0+\beta$	$2\alpha_0+\gamma$	$2\alpha_0$

This model can be seen to correspond to a restricted form of our ealier 10-parameter model, in which we reparameterise the parameters in the 10-parameter model as follows: $\alpha=2\alpha_0$, $\beta_1=\beta$, $\beta_2=2\beta$ $\gamma_1=\gamma$, $\gamma_2=2\gamma$, $\delta_{12}=\delta_{21}=\delta'_{11}=\delta$, $\delta_{22}=2\delta$, $\delta_{11}=0$.

Proof of equivalance between Wu et al.'s test and restricted logistic regression model

Here we prove that, provided we make a rare disease assumption, the parameter δ in the above restricted form of the 10-parameter logistic regression model does indeed correspond precisely to the parameter tested by Wu et al. (2010) [1].

Following the notation of Wu et al. (2010) [1], we denote the penetrance for each cell in the 16-cell table as f_{jklm} where f_{jklm} represents the probability of being diseased for an individual with diplotype G_j-H_k/G_l-H_m. Wu et al. (2010) [1] define their 'interaction' odds ratio of interest as

$$\left[\frac{h_{11}h_{22}}{h_{12}h_{21}}\right] \Big/ \left[\frac{(1-h_{11})(1-h_{22})}{(1-h_{12})(1-h_{21})}\right]$$

where h_{jk} is the so so-called 'penetrance' of haplotype G_j-H_k:

$$h_{jk} = \psi_{11}f_{jk11} + \psi_{12}f_{jk12} + \psi_{21}f_{jk21} + \psi_{22}f_{jk22}$$

This concept of 'penetrance' of a haplotype is slightly complicated to understand, but appears to represent some kind of weighted average of the penetrances for diplotypes involving that haplotype, averaged over the possibilities for the other haplotype in the diplotype. Wu et al. (2010) [1] show that their overall log odds ratio of interest using this definition can be reduced to

$$\log\frac{P_{11}^A P_{22}^A}{P_{12}^A P_{21}^A} - \log\frac{P_{11}^N P_{22}^N}{P_{12}^N P_{21}^N}$$

where P_{jk}^A and P_{jk}^N refer to haplotype frequencies in cases and controls respectively, which corresponds to the formulation given in Equations 3 and 5 in our main manuscript.

Our proposed restricted logistic regression model would imply that the diplotype penetrances f_{jklm} take the following form:

Paternal haplotype	Maternal haplotype G_1-H_1	G_1-H_2	G_2-H_1	G_2-H_2
G_1-H_1	$\frac{e^{\alpha+2\beta+2\gamma+2\delta}}{1+e^{\alpha+2\beta+2\gamma+2\delta}}$	$\frac{e^{\alpha+2\beta+\gamma+\delta}}{1+e^{\alpha+2\beta+\gamma+\delta}}$	$\frac{e^{\alpha+\beta+2\gamma+\delta}}{1+e^{\alpha+\beta+2\gamma+\delta}}$	$\frac{e^{\alpha+\beta+\gamma+\delta}}{1+e^{\alpha+\beta+\gamma+\delta}}$
G_1-H_2	$\frac{e^{\alpha+2\beta+\gamma+\delta}}{1+e^{\alpha+2\beta+\gamma+\delta}}$	$\frac{e^{\alpha+2\beta}}{1+e^{\alpha+2\beta}}$	$\frac{e^{\alpha+\beta+\gamma}}{1+e^{\alpha+\beta+\gamma}}$	$\frac{e^{\alpha+\beta}}{1+e^{\alpha+\beta}}$
G_2-H_1	$\frac{e^{\alpha+\beta+2\gamma+\delta}}{1+e^{\alpha+\beta+2\gamma+\delta}}$	$\frac{e^{\alpha+\beta+\gamma}}{1+e^{\alpha+\beta+\gamma}}$	$\frac{e^{\alpha+2\gamma}}{1+e^{\alpha+2\gamma}}$	$\frac{e^{\alpha+\gamma}}{1+e^{\alpha+\gamma}}$
G_2-H_2	$\frac{e^{\alpha+\beta+\gamma+\delta}}{1+e^{\alpha+\beta+\gamma+\delta}}$	$\frac{e^{\alpha+\beta}}{1+e^{\alpha+\beta}}$	$\frac{e^{\alpha+\gamma}}{1+e^{\alpha+\gamma}}$	$\frac{e^{\alpha}}{1+e^{\alpha}}$

Using this formulation for the diplotype penetrances f_{jklm}, we may write down an expression for Wu et al.'s interaction log odds ratio of interest in terms of the parameters α, β, γ, δ. This expression is complicated and, in general, Wu et al.'s log odds ratio does not turn out to precisely correspond to δ. However, if we are willing to make a rare disease assumption, the penetrances f_{jklm} may be written

Paternal haplotype	Maternal haplotype G_1-H_1	G_1-H_2	G_2-H_1	G_2-H_2
G_1-H_1	$e^{\alpha+2\beta+2\gamma+2\delta}$	$e^{\alpha+2\beta+\gamma+\delta}$	$e^{\alpha+\beta+2\gamma+\delta}$	$e^{\alpha+\beta+\gamma+\delta}$
G_1-H_2	$e^{\alpha+2\beta+\gamma+\delta}$	$e^{\alpha+2\beta}$	$e^{\alpha+\beta+\gamma}$	$e^{\alpha+\beta}$
G_2-H_1	$e^{\alpha+\beta+2\gamma+\delta}$	$e^{\alpha+\beta+\gamma}$	$e^{\alpha+2\gamma}$	$e^{\alpha+\gamma}$
G_2-H_2	$e^{\alpha+\beta+\gamma+\delta}$	$e^{\alpha+\beta}$	$e^{\alpha+\gamma}$	e^{α}

and, moreover, the denominator of Wu et al.'s odds ratio

$$\frac{(1-h_{11})(1-h_{22})}{(1-h_{12})(1-h_{21})} \approx 1.$$

Therefore, Wu et al.'s odds ratio of interest is reduced to

$$\begin{aligned}\frac{h_{11}h_{22}}{h_{12}h_{21}} &= \frac{(\psi_{11}f_{1111}+\psi_{12}f_{1112}+\psi_{21}f_{1121}+\psi_{22}f_{1122})(\psi_{11}f_{1211}+\psi_{12}f_{1212}+\psi_{21}f_{1221}+\psi_{22}f_{1222})}{(\psi_{11}f_{2111}+\psi_{12}f_{2112}+\psi_{21}f_{2121}+\psi_{22}f_{2122})(\psi_{11}f_{2211}+\psi_{12}f_{2212}+\psi_{21}f_{2221}+\psi_{22}f_{2222})} \\ &= \frac{e^{\alpha}(\psi_{11}e^{2\beta+2\gamma+2\delta}+\psi_{12}e^{2\beta+\gamma+\delta}+\psi_{21}e^{\beta+2\gamma+\delta}+\psi_{22}e^{\beta+\gamma+\delta})e^{\alpha}(\psi_{11}e^{\beta+\gamma+\delta}+\psi_{12}e^{\beta}+\psi_{21}e^{\gamma}+\psi_{22})}{e^{\alpha}(\psi_{11}e^{2\beta+\gamma+\delta}+\psi_{12}e^{2\beta}+\psi_{21}e^{\beta+\gamma}+\psi_{22}e^{\beta})e^{\alpha}(\psi_{11}e^{\beta+2\gamma+\delta}+\psi_{12}e^{\beta+\gamma}+\psi_{21}e^{2\gamma}+\psi_{22}e^{\gamma})}\end{aligned}$$

$$
\begin{aligned}
= \quad & [\psi_{11}^2 e^{3\beta+3\gamma+3\delta} + 2\psi_{11}\psi_{12}e^{3\beta+2\gamma+2\delta} + 2\psi_{11}\psi_{21}e^{2\beta+3\gamma+2\delta} + 2\psi_{11}\psi_{22}e^{2\beta+2\gamma+2\delta} + \psi_{12}^2 e^{3\beta+\gamma+\delta} \\
& +2\psi_{12}\psi_{21}e^{2\beta+2\gamma+\delta} + 2\psi_{12}\psi_{22}e^{2\beta+\gamma+\delta} + \psi_{21}^2 e^{\beta+3\gamma+\delta} + 2\psi_{21}\psi_{22}e^{\beta+2\gamma+\delta} + \psi_{22}^2 e^{\beta+\gamma+\delta}] \\
& /[\psi_{11}^2 e^{3\beta+3\gamma+2\delta} + 2\psi_{11}\psi_{12}e^{3\beta+2\gamma+\delta} + 2\psi_{11}\psi_{21}e^{2\beta+3\gamma+\delta} + 2\psi_{11}\psi_{22}e^{2\beta+2\gamma+\delta} + \psi_{12}^2 e^{3\beta+\gamma} \\
& +2\psi_{12}\psi_{21}e^{2\beta+2\gamma} + 2\psi_{12}\psi_{22}e^{2\beta+\gamma} + \psi_{21}^2 e^{\beta+3\gamma} + 2\psi_{21}\psi_{22}e^{\beta+2\gamma} + \psi_{22}^2 e^{\beta+\gamma}] \\
= \quad & e^{\delta}
\end{aligned}
$$

Thus, under a rare disease assumption, the odds ratio used as the basis of the Wu et al. (2010) statistic can indeed be seen to correspond to e^{δ} (and thus the log odds ratio corresponds to δ) where δ is the interaction term in the following restricted logistic regression formulation for the log odds of disease:

Paternal haplotype	Maternal haplotype G_1-H_1	G_1-H_2	G_2-H_1	G_2-H_2
G_1-H_1	$\alpha + 2\beta + 2\gamma + 2\delta$	$\alpha + 2\beta + \gamma + \delta$	$\alpha + \beta + 2\gamma + \delta$	$\alpha + \beta + \gamma + \delta$
G_1-H_2	$\alpha + 2\beta + \gamma + \delta$	$\alpha + 2\beta$	$\alpha + \beta + \gamma$	$\alpha + \beta$
G_2-H_1	$\alpha + \beta + 2\gamma + \delta$	$\alpha + \beta + \gamma$	$\alpha + 2\gamma$	$\alpha + \gamma$
G_2-H_2	$\alpha + \beta + \gamma + \delta$	$\alpha + \beta$	$\alpha + \gamma$	α

Since diplotypes are not observed, it is not usually possible to implement this logistic regression model in practice, although in principal one could attempt to fit it using missing data likelihood methods (e.g. via an EM algorithm [6]).

Improved test of λ via joint effects statistic

We have seen that, under a rare disease assumption, the interaction log odds ratio tested by Wu et al. corresponds to the parameter δ in the above logistic regression formulation. Nevertheless, Wu et al. do not estimate δ directly but rather test whether it equals 0 by constructing two log odds ratios

$$\lambda_A = \log \frac{P_{11}^A P_{22}^A}{P_{12}^A P_{21}^A}$$

$$\lambda_N = \log \frac{P^N_{11} P^N_{22}}{P^N_{12} P^N_{21}}$$

(where P^A_{jk} and P^N_{jk} refer to haplotype frequencies in cases and controls respectively), and by then testing whether $\lambda_A = 0$ (case-only test) or whether $\lambda_A = \lambda_N$ (case/control test). We propose using a similar idea, but using a test that allows for more general main effects than in the Wu formulation (which makes restrictions on the main effects at each of the two loci, namely that alleles act additively on the log odds scale within each locus). Our motivation for making this improvement is the fear that, if the model assumed by Wu et al. is misspecified with respect to the main effects, then it is possible that inference with respect to interaction effects might be affected.

First we return to our more general 10-parameter model for the log odds for the different diplotypes:

Paternal haplotype	Maternal haplotype G_1-H_1	G_1-H_2	G_2-H_1	G_2-H_2
G_1-H_1	$\alpha + \beta_2 + \gamma_2 + \delta_{22}$	$\alpha + \beta_2 + \gamma_1 + \delta_{21}$	$\alpha + \beta_1 + \gamma_2 + \delta_{12}$	$\alpha + \beta_1 + \gamma_1 + \delta'_{11}$
G_1-H_2	$\alpha + \beta_2 + \gamma_1 + \delta_{21}$	$\alpha + \beta_2$	$\alpha + \beta_1 + \gamma_1 + \delta_{11}$	$\alpha + \beta_1$
G_2-H_1	$\alpha + \beta_1 + \gamma_2 + \delta_{12}$	$\alpha + \beta_1 + \gamma_1 + \delta_{11}$	$\alpha + \gamma_2$	$\alpha + \gamma_1$
G_2-H_2	$\alpha + \beta_1 + \gamma_1 + \delta'_{11}$	$\alpha + \beta_1$	$\alpha + \gamma_1$	α

Following Wu's idea of estimating quantites in cases and controls separately, we derive the distribution of the diplotypes in cases and controls that results from the above model. In cases, this distribution is given by $P((G_j\text{-}H_k/G_l\text{-}H_m)|D)$, which by Bayes' theorem (assuming HWE in the general population) equals $f_{jklm}\psi_{jk}\psi_{lm}/K$, where f_{jklm} is the probability of being diseased for an individual with diplotype G_j-H_k/G_l-H_m, ψ_{jk} represents the population frequency of haplotype G_j-H_k, D represents the event that an individual is affected (diseased) and $K = P(D)$ is the population prevalance. Similarly, the distribution of diplotypes in controls is given by $(1 - f_{jklm})\psi_{jk}\psi_{lm}/(1 - K)$. Thus, the diplotype probabilities in cases and controls may be written:

Cases	Maternal haplotype			
Paternal haplotype	G_1-H_1	G_1-H_2	G_2-H_1	G_2-H_2
G_1-H_1	$\psi_{11}^2 f_{1111}/K$	$\psi_{11}\psi_{12} f_{1112}/K$	$\psi_{11}\psi_{21} f_{1121}/K$	$\psi_{11}\psi_{22} f_{1122}/K$
G_1-H_2	$\psi_{12}\psi_{11} f_{1211}/K$	$\psi_{12}^2 f_{1212}/K$	$\psi_{12}\psi_{21} f_{1221}/K$	$\psi_{12}\psi_{22} f_{1222}/K$
G_2-H_1	$\psi_{21}\psi_{11} f_{2111}/K$	$\psi_{21}\psi_{12} f_{2112}/K$	$\psi_{21}^2 f_{2121}/K$	$\psi_{21}\psi_{22} f_{2122}/K$
G_2-H_2	$\psi_{22}\psi_{11} f_{2211}/K$	$\psi_{22}\psi_{12} f_{2212}/K$	$\psi_{22}\psi_{21} f_{2221}/K$	$\psi_{22}^2 f_{2222}/K$

Controls	Maternal haplotype			
Paternal haplotype	G_1-H_1	G_1-H_2	G_2-H_1	G_2-H_2
G_1-H_1	$\frac{\psi_{11}^2(1-f_{1111})}{(1-K)}$	$\frac{\psi_{11}\psi_{12}(1-f_{1112})}{(1-K)}$	$\frac{\psi_{11}\psi_{21}(1-f_{1121})}{(1-K)}$	$\frac{\psi_{11}\psi_{22}(1-f_{1122})}{(1-K)}$
G_1-H_2	$\frac{\psi_{12}\psi_{11}(1-f_{1211})}{(1-K)}$	$\frac{\psi_{12}^2(1-f_{1212})}{(1-K)}$	$\frac{\psi_{12}\psi_{21}(1-f_{1221})}{(1-K)}$	$\frac{\psi_{12}\psi_{22}(1-f_{1222})}{(1-K)}$
G_2-H_1	$\frac{\psi_{21}\psi_{11}(1-f_{2111})}{(1-K)}$	$\frac{\psi_{21}\psi_{12}(1-f_{2112})}{(1-K)}$	$\frac{\psi_{21}^2(1-f_{2121})}{(1-K)}$	$\frac{\psi_{21}\psi_{22}(1-f_{2122})}{(1-K)}$
G_2-H_2	$\frac{\psi_{22}\psi_{11}(1-f_{2211})}{(1-K)}$	$\frac{\psi_{22}\psi_{12}(1-f_{2212})}{(1-K)}$	$\frac{\psi_{22}\psi_{21}(1-f_{2221})}{(1-K)}$	$\frac{\psi_{22}^2(1-f_{2222})}{(1-K)}$

Under the 10-parameter diplotype model, and making a rare disease assumption, these diplotype probabilities in cases and controls reduce to:

Cases	Maternal haplotype			
Paternal haplotype	G_1-H_1	G_1-H_2	G_2-H_1	G_2-H_2
G_1-H_1	$\frac{\psi_{11}^2 e^{\alpha+\beta_2+\gamma_2+\delta_{22}}}{K}$	$\frac{\psi_{11}\psi_{12} e^{\alpha+\beta_2+\gamma_1+\delta_{21}}}{K}$	$\frac{\psi_{11}\psi_{21} e^{\alpha+\beta_1+\gamma_2+\delta_{12}}}{K}$	$\frac{\psi_{11}\psi_{22} e^{\alpha+\beta_1+\gamma_1+\delta'_{11}}}{K}$
G_1-H_2	$\frac{\psi_{12}\psi_{11} e^{\alpha+\beta_2+\gamma_1+\delta_{21}}}{K}$	$\frac{\psi_{12}^2 e^{\alpha+\beta_2}}{K}$	$\frac{\psi_{12}\psi_{21} e^{\alpha+\beta_1+\gamma_1+\delta_{11}}}{K}$	$\frac{\psi_{12}\psi_{22} e^{\alpha+\beta_1}}{K}$
G_2-H_1	$\frac{\psi_{21}\psi_{11} e^{\alpha+\beta_1+\gamma_2+\delta_{12}}}{K}$	$\frac{\psi_{21}\psi_{12} e^{\alpha+\beta_1+\gamma_1+\delta_{11}}}{K}$	$\frac{\psi_{21}^2 e^{\alpha+\gamma_2}}{K}$	$\frac{\psi_{21}\psi_{22} e^{\alpha+\gamma_1}}{K}$
G_2-H_2	$\frac{\psi_{22}\psi_{11} e^{\alpha+\beta_1+\gamma_1+\delta'_{11}}}{K}$	$\frac{\psi_{22}\psi_{12} e^{\alpha+\beta_1}}{K}$	$\frac{\psi_{22}\psi_{21} e^{\alpha+\gamma_1}}{K}$	$\frac{\psi_{22}^2 e^{\alpha}}{K}$

Controls	Maternal haplotype			
Paternal haplotype	G_1-H_1	G_1-H_2	G_2-H_1	G_2-H_2
G_1-H_1	ψ_{11}^2	$\psi_{11}\psi_{12}$	$\psi_{11}\psi_{21}$	$\psi_{11}\psi_{22}$
G_1-H_2	$\psi_{12}\psi_{11}$	ψ_{12}^2	$\psi_{12}\psi_{21}$	$\psi_{12}\psi_{22}$
G_2-H_1	$\psi_{21}\psi_{11}$	$\psi_{21}\psi_{12}$	ψ_{21}^2	$\psi_{21}\psi_{22}$
G_2-H_2	$\psi_{22}\psi_{11}$	$\psi_{22}\psi_{12}$	$\psi_{22}\psi_{21}$	ψ_{22}^2

Therefore, combining cells of the above table appropriately, the probabilities of the 9 observable genotype combinations in cases and controls are:

Cases	Locus H		
Locus G	H_1H_1	H_1H_2	H_2H_2
G_1G_1	$\frac{\psi_{11}^2 e^{\alpha+\beta_2+\gamma_2+\delta_{22}}}{K}$	$\frac{2\psi_{11}\psi_{12}e^{\alpha+\beta_2+\gamma_1+\delta_{21}}}{K}$	$\frac{\psi_{12}^2 e^{\alpha+\beta_2}}{K}$
G_1G_2	$\frac{2\psi_{11}\psi_{21}e^{\alpha+\beta_1+\gamma_2+\delta_{12}}}{K}$	$\frac{2\psi_{11}\psi_{22}e^{\alpha+\beta_1+\gamma_1+\delta'_{11}}+2\psi_{12}\psi_{21}e^{\alpha+\beta_1+\gamma_1+\delta_{11}}}{K}$	$\frac{2\psi_{12}\psi_{22}e^{\alpha+\beta_1}}{K}$
G_2G_2	$\frac{\psi_{21}^2 e^{\alpha+\gamma_2}}{K}$	$\frac{2\psi_{21}\psi_{22}e^{\alpha+\gamma_1}}{K}$	$\frac{\psi_{22}^2 e^{\alpha}}{K}$

Controls	Locus H		
Locus G	H_1H_1	H_1H_2	H_2H_2
G_1G_1	ψ_{11}^2	$2\psi_{11}\psi_{12}$	ψ_{12}^2
G_1G_2	$2\psi_{11}\psi_{21}$	$2\psi_{11}\psi_{22} + 2\psi_{12}\psi_{21}$	$2\psi_{12}\psi_{22}$
G_2G_2	ψ_{21}^2	$2\psi_{21}\psi_{22}$	ψ_{22}^2

Our joint effects test is based on constructing (within cases and controls separately) four odds ratios (i_{22}, i_{21}, i_{12}, i_{11}) by using each of the four top left cells in turn, to estimate the odds ratio relative to the baseline (bottom right) cell. The motivation for this approach is that any main effects will cancel out, see Text S2. Given the distribution of the 9 observable genotype combinations above, we can see that in controls these odds ratios correspond to the following quantities:

$$i_{22} = \frac{\psi_{11}^2\psi_{22}^2}{\psi_{12}^2\psi_{21}^2} = (e^{\lambda_\psi})^2 = e^{2\lambda_\psi}$$

$$i_{21} = \frac{2\psi_{11}\psi_{12}\psi_{22}^2}{2\psi_{21}\psi_{22}\psi_{12}^2} = e^{\lambda_\psi}$$

$$i_{12} = \frac{2\psi_{11}\psi_{21}\psi_{22}^2}{2\psi_{12}\psi_{22}\psi_{21}^2} = e^{\lambda_\psi}$$

$$i_{11} = \frac{2\psi_{22}^2[\psi_{11}\psi_{22} + \psi_{12}\psi_{21}]}{4\psi_{12}\psi_{22}\psi_{21}\psi_{22}} = \frac{e^{\lambda_\psi} + 1}{2}$$

where λ_ψ is the log odds ratio measure defined with respect to haplotype frequencies in the general population.

For cases, the quantities estimated by the 4 odds ratios depend on the paramaterisation chosen for the interaction parameters (but not on the main effects parameters, which all cancel out as desired). Suppose we imposed a paramaterisation for interaction effects corresponding to that imposed by Wu et al. i.e. $\delta_{12} = \delta_{21} = \delta'_{11} = \delta$, $\delta_{22} = 2\delta$, $\delta_{11} = 0$. Then, in cases, the four odds ratios end up corresponding to the following quantities :

$$i_{22} = \frac{\psi_{11}^2\psi_{22}^2}{\psi_{12}^2\psi_{21}^2}e^{\delta_{22}} = (e^{\lambda_\psi})^2 e^{2\delta} = e^{2(\lambda_\psi+\delta)} = e^{2\lambda_A}, \text{ say}$$

$$i_{21} = \frac{2\psi_{11}\psi_{12}\psi_{22}^2}{2\psi_{21}\psi_{22}\psi_{12}^2}e^{\delta_{21}} = e^{\lambda_\psi}e^{\delta} = e^{\lambda_\psi+\delta} = e^{\lambda_A}$$

$$i_{12} = \frac{2\psi_{11}\psi_{21}\psi_{22}^2}{2\psi_{12}\psi_{22}\psi_{21}^2}e^{\delta_{12}} = e^{\lambda_\psi}e^{\delta} = e^{\lambda_\psi+\delta} = e^{\lambda_A}$$

$$\begin{aligned} i_{11} &= \frac{2\psi_{22}^2[\psi_{11}\psi_{22}e^{\delta'_{11}} + \psi_{12}\psi_{21}e^{\delta_{11}}]}{4\psi_{12}\psi_{22}\psi_{21}\psi_{22}} = \frac{\psi_{11}\psi_{22}e^{\delta} + \psi_{12}\psi_{21}}{2\psi_{12}\psi_{21}} \\ &= \frac{1}{2}(e^{\lambda_\psi}e^{\delta}+1) = \frac{e^{\lambda_\psi+\delta}+1}{2} = \frac{e^{\lambda_A}+1}{2} \end{aligned}$$

where λ_A is defined as $\lambda_\psi + \delta$. Thus, by estimating (i_{11}, i_{12}, i_{21}, i_{22}) in cases and controls separately, and considering these quantities as the following functions of a parameter λ:

$$i_{22} = e^{2\lambda} \qquad i_{21} = e^{\lambda} \qquad i_{12} = e^{\lambda} \qquad i_{11} = \frac{e^{\lambda}+1}{2}$$

we can see that in controls, the quantity λ corresponds to the usual log odds ratio measure λ_ψ defined with respect to haplotype frequencies in the general population, while in cases the quantity λ corresponds to $\lambda_A = \lambda_\psi + \delta$, where δ is Wu et al.'s interaction parameter of interest. Therefore, we may test whether $\delta = 0$ by testing whether the parameter λ as estimated (on the basis of i_{11}, i_{12}, i_{21}, i_{22}) in cases equals λ as estimated in controls (case/control test). Alternatively, if we are willing to assume no population level LD (so $\lambda_\psi = 0$), we may instead test whether $\delta = 0$ by testing whether λ as estimated in cases equals 0

(case-only test).

Alternative versions of joint effects statistics

The motivation for the joint effects statistics given above also allows the possibility of imposing alternative parameterisations for the interaction parameters. Suppose we wished to impose the usual 9-parameter 'saturated' model for combinations of genotypes at the two loci:

	Locus H		
Locus G	H_1H_1	H_1H_2	H_2H_2
G_1G_1	$\alpha+\beta_2+\gamma_2+\delta_{22}$	$\alpha+\beta_2+\gamma_1+\delta_{21}$	$\alpha+\beta_2$
G_1G_2	$\alpha+\beta_1+\gamma_2+\delta_{12}$	$\alpha+\beta_1+\gamma_1+\delta_{11}$	$\alpha+\beta_1$
G_2G_2	$\alpha+\gamma_2$	$\alpha+\gamma_1$	α

Then, using the same argument as in the previous section, the four odds ratios (i_{11}, i_{12}, i_{21}, i_{22}) used in the joint effects test end up corresponding to estimates of the following quantities in controls:

$$i_{22} = e^{2\lambda_\psi} \qquad i_{21} = e^{\lambda_\psi} \qquad i_{12} = e^{\lambda_\psi} \qquad i_{11} = \frac{e^{\lambda\psi}+1}{2}$$

while in cases they end up estimating:

$$i_{22} = e^{2\lambda_\psi} e^{\delta_{22}} \qquad i_{21} = e^{\lambda_\psi} e^{\delta_{21}} \qquad i_{12} = e^{\lambda_\psi} e^{\delta_{12}} \qquad i_{11} = \frac{e^{\lambda\psi}+1}{2} e^{\delta_{11}}$$

We may thus test each interaction effect individually by testing whether the relevant odds ratio (i_{11}, i_{12}, i_{21} or i_{22}) is equal when estimated in cases as in controls (case/control test) or, assuming no population level LD, by testing whether the relevant odds ratio estimated in cases alone equals 1. This strategy would lead to four 1df tests or an overall 4df test when considering all interaction effects simultaneously. To increase power, one might prefer to reparameterise in terms of a single parameter δ, for example assuming an allelic coding whereby $\delta_{11} = \delta$, $\delta_{12} = \delta_{21} = 2\delta$, $\delta_{22} = 4\delta$. (This coding would be similar to the model assumed in PLINK). In that case, the odds ratios in cases end up estimating:

$$i_{22} = e^{2\lambda_\psi} e^{4\delta} \qquad i_{21} = e^{\lambda_\psi} e^{2\delta} \qquad i_{12} = e^{\lambda_\psi} e^{2\delta} \qquad i_{11} = \frac{e^{\lambda\psi}+1}{2} e^{\delta}$$

while the odds ratios in controls estimate

$$i_{22} = e^{2\lambda_\psi} \qquad i_{21} = e^{\lambda_\psi} \qquad i_{12} = e^{\lambda_\psi} \qquad i_{11} = \frac{e^{\lambda\psi} + 1}{2}$$

as before. Denoting the odds ratios estimated in cases as (i^A_{11}, i^A_{12}, i^A_{21}, i^A_{22}) and those in controls as (i^N_{11}, i^N_{12}, i^N_{21}, i^N_{22}), we could therefore construct 4 separate estimates of δ:

$$\hat{\delta}_1 = \frac{\log(i^A_{22}/i^N_{22})}{4} \qquad \hat{\delta}_2 = \frac{\log(i^A_{21}/i^N_{21})}{2} \qquad \hat{\delta}_3 = \frac{\log(i^A_{12}/i^N_{12})}{2} \qquad \hat{\delta}_4 = \log(i^A_{11}/i^N_{11})$$

Similar to the joint effects test of λ described previously, we could use a weighted average of these four estimates (with weights chosen to make the variance minimum), divided by its estimated variance, as a direct test of the parameter δ. We defer any detailed derivation and investigation of the properties of this (and other) alternative parameterisations of the joint effects test to future work.

References

1. Wu X, Dong H, Luo L, Zhu Y, Peng G, et al. (2010) A novel statistic for genome-wide interaction analysis. PLoS Genet 6: e1001131.

2. Piegorsch WW, Weinberg CR, Taylor JA (1994) Non-hierarchical logistic models and case-only designs for assessing susceptibility in population-based case-control studies. Statistics in Medicine 13: 153–162.

3. Yang Q, Khoury MJ, Sun F, Flanders WD (1999) Case-only design to measure gene-gene interaction. Epidemiology 10: 167-170.

4. Weinberg CR, Umbach DM (2000) Choosing a retrospective design to assess joint genetic and environmental contributions to risk. Am J Epidemiol 152: 197–203.

5. Sasieni P (1997) From genotypes to genes: doubling the sample size. Biometrics 53: 1253-1261.

6. Dempster A, Laird N, Rubin D (1977) Maximum likelihood from incomplete data via the EM algorithm. Journal of the Royal Statistical Society, Series B 39: 1–22.
