# Supplementary material for: Improved Statistics for Genome-Wide Interaction Analysis
Source: PLoS Genet. 2012 Apr 5;8(4):e1002625. doi: 10.1371/journal.pgen.1002625 (PMC3320596; doi:10.1371/journal.pgen.1002625)
Supplement: Text S4 — Here we consider the relationship between the fast-epistasis (FE) and Wellek and Ziegler (WZ) inspired statistics and standard logistic and linear regression. (PDF) [file pgen.1002625.s016.pdf]

Text S4

Here we consider the relationship between the *fast-epistasis* (FE) and Wellek and Ziegler (WZ) inspired statistics and standard logistic and linear regression.

PLINK's *fast-epistasis* statistic

The *fast-epistasis* statistic in PLINK [1] is motivated on the PLINK website as a faster alternative to the standard allelic effects logistic regression-based test (obtained in PLINK via the `--epistasis` option):

$$\log \frac{p}{1-p} \quad = \quad \alpha + \beta x_1 + \gamma x_2 + \delta x_1 x_2$$

where predictor variables x_1 and x_2 are coded (0,1,2), according to the number of susceptibility alleles possessed at locus G and H, respectively. On the PLINK website it states that the formulation of the *fast-epistasis* statistic "assumes Hardy-Weinberg and linkage equilibrium for the two SNPs in the population", although simulations (using less extreme models than are considered here in this manuscript) "have shown the case/control test to be very robust to deviations from the linkage equilibrium assumption". It is therefore of interest to determine to what extent the odds ratio estimated in the *fast-epistasis* method estimates the desired interaction parameter δ within this allelic model, particularly under the conditions of HWE and linkage equilibrium.

In Text S2 we showed that, under a rare disease assumption, assuming main effects at both loci (denoted $A = e^{\alpha}$, $B_1 = e^{\beta_1}$, $B_2 = e^{\beta_2}$, $C_1 = e^{\gamma_1}$, $C_2 = e^{\gamma_2}$) and assuming HWE and linkage equilibrium, the odds ratio measure in cases reduces to

$$\mathrm{OR}_{\mathrm{FE},A} \quad = \quad \left(\frac{B_2(C_2\psi_{11} + C_1\psi_{12}) + B_1(C_2\psi_{21} + C_1\psi_{22})}{B_2(\psi_{12} + C_1\psi_{11}) + B_1(\psi_{22} + C_1\psi_{21})} \right) \times \left(\frac{(\psi_{22} + C_1\psi_{21}) + B_1(\psi_{12} + C_1\psi_{11})}{(C_2\psi_{21} + C_1\psi_{22}) + B_1(C_2\psi_{11} + C_1\psi_{12})} \right)$$

while that in controls reduces to 1. Adding in an interaction effect δ, and making an assumption of allelic effects (so that $B_1 = B$, $C_1 = C$, $B_2 = B^2$, $C_2 = C^2$, for some parameters B and C), the odds ratio measure used by the *fast-epistasis* statistic PLINK in cases becomes

$$
\begin{aligned}
\mathrm{OR}_{\mathrm{FE},A} &= \left(\frac{\psi_{11}B^2C^2e^{4\delta}+\psi_{12}B^2Ce^{2\delta}+\psi_{21}BC^2e^{2\delta}+\psi_{22}BCe^{\delta}}{\psi_{12}B^2+\psi_{11}B^2Ce^{2\delta}+\psi_{22}B+\psi_{21}BCe^{\delta}}\right)\times\left(\frac{\psi_{22}+\psi_{21}C+\psi_{12}B+\psi_{11}BCe^{\delta}}{\psi_{21}C^2+\psi_{22}C+\psi_{11}BC^2e^{2\delta}+\psi_{12}BCe^{\delta}}\right)\\
&= \frac{(\psi_{11}BCe^{4\delta}+\psi_{12}Be^{2\delta}+\psi_{21}Ce^{2\delta}+\psi_{22}e^{\delta})(\psi_{11}BCe^{\delta}+\psi_{12}B+\psi_{21}C+\psi_{22})}{(\psi_{11}BCe^{2\delta}+\psi_{12}B+\psi_{21}Ce^{\delta}+\psi_{22})(\psi_{11}BCe^{2\delta}+\psi_{12}Be^{\delta}+\psi_{21}C+\psi_{22})}\\
&= \{\psi_{11}^2B^2C^2e^{5\delta}+\psi_{11}\psi_{12}[B^2Ce^{4\delta}+B^2Ce^{3\delta}]+\psi_{11}\psi_{21}[BC^2e^{4\delta}+BC^2e^{3\delta}]+\psi_{11}\psi_{22}[BCe^{4\delta}+BCe^{2\delta}]\\
&\quad+\psi_{12}^2B^2e^{2\delta}+\psi_{12}\psi_{21}[BCe^{2\delta}+BCe^{2\delta}]+\psi_{12}\psi_{22}[Be^{2\delta}+Be^{\delta}]+\psi_{21}^2C^2e^{2\delta}+\psi_{21}\psi_{22}[Ce^{2\delta}+Ce^{\delta}]+\psi_{22}^2e^{\delta}\}\\
&\quad/\{\psi_{11}^2B^2C^2e^{4\delta}+\psi_{11}\psi_{12}[B^2Ce^{3\delta}+B^2Ce^{2\delta}]+\psi_{11}\psi_{21}[BC^2e^{2\delta}+BC^2e^{3\delta}]+\psi_{11}\psi_{22}[BCe^{2\delta}+BCe^{2\delta}]\\
&\quad+\psi_{12}^2B^2e^{\delta}+\psi_{12}\psi_{21}[BC+BCe^{2\delta}]+\psi_{12}\psi_{22}[B+Be^{\delta}]+\psi_{21}^2C^2e^{\delta}+\psi_{21}\psi_{22}[Ce^{\delta}+C]+\psi_{22}^2\}\\
&= \{e^{\delta}(\psi_{11}^2B^2C^2e^{4\delta}+\psi_{11}\psi_{12}[B^2Ce^{3\delta}+B^2Ce^{2\delta}]+\psi_{11}\psi_{21}[BC^2e^{2\delta}+BC^2e^{3\delta}]+\psi_{12}^2B^2e^{\delta}+\psi_{12}\psi_{22}[B+Be^{\delta}]\\
&\quad+\psi_{21}^2C^2e^{\delta}+\psi_{21}\psi_{22}[Ce^{\delta}+C]+\psi_{22}^2)+\psi_{11}\psi_{22}[BCe^{4\delta}+BCe^{2\delta}]+\psi_{12}\psi_{21}[BCe^{2\delta}+BCe^{2\delta}]\}\\
&\quad/\{(\psi_{11}^2B^2C^2e^{4\delta}+\psi_{11}\psi_{12}[B^2Ce^{3\delta}+B^2Ce^{2\delta}]+\psi_{11}\psi_{21}[BC^2e^{2\delta}+BC^2e^{3\delta}]+\psi_{12}^2B^2e^{\delta}+\psi_{12}\psi_{22}[B+Be^{\delta}]\\
&\quad+\psi_{21}^2C^2e^{\delta}+\psi_{21}\psi_{22}[Ce^{\delta}+C]+\psi_{22}^2)+\psi_{11}\psi_{22}[BCe^{2\delta}+BCe^{2\delta}]+\psi_{12}\psi_{21}[BC+BCe^{2\delta}]\}
\end{aligned}
$$

We can see that most of the terms in the numerator equal e^{δ} times a corresponding term in the denominator. If this was true for all terms, the everything would cancel and the odds ratio would equal e^{δ}, as desired. However, two 'problem' terms do not follow this pattern, namely the terms in $\psi_{11}\psi_{22}$ and $\psi_{12}\psi_{21}$, meaning that the odds ratio can instead only be written

$$
\mathrm{OR}_{\mathrm{FE},A} = \frac{\{e^{\delta}L+\psi_{11}\psi_{22}[BCe^{4\delta}+BCe^{2\delta}]+\psi_{12}\psi_{21}[BCe^{2\delta}+BCe^{2\delta}]\}}{\{L+\psi_{11}\psi_{22}[BCe^{2\delta}+BCe^{2\delta}]+\psi_{12}\psi_{21}[BC+BCe^{2\delta}]\}},
$$

where L is defined appropriately.

Recall that if there is no LD, we may write $\psi_{11}=pu$, $\psi_{21}=qu$, $\psi_{12}=pv$, $\psi_{22}=qv$, where p,q,u,v are the allele frequencies of G_1, G_2, H_1, H_2 respectively. Then the odds ratio reduces to

$$\begin{aligned} \mathrm{OR}_{\mathrm{FE},A} &= \frac{\{e^{\delta}L + pquvBC[e^{4\delta} + e^{2\delta} + e^{2\delta} + e^{2\delta}]\}}{\{L + pquvBC[e^{2\delta} + e^{2\delta} + 1 + e^{2\delta}]\}} \\ &= \frac{\{e^{\delta}L + pquvBC[3e^{2\delta} + e^{4\delta}]\}}{\{L + pquvBC[3e^{2\delta} + 1]\}} \end{aligned}$$

Considering the right hand term in the numerator and denominator, the terms still do not cancel out, and the odds ratio estimated by the *fast-epistasis* statistic in PLINK is thus not precisely equivalent to the desired interaction odds ratio. However, under the null hypothesis of no interaction ($\delta = 0$), $\mathrm{OR}_{\mathrm{FE},A}$ does reduce to 1, suggesting that, provided there is no population level LD, $\mathrm{OR}_{\mathrm{FE},A}$ (or the difference between $\log \mathrm{OR}_{\mathrm{FE},A}$ and $\log \mathrm{OR}_{\mathrm{FE},N}$) should provide valid inference with respect to *testing* the null hypothesis, even though the parameter estimated is not precisely equal to the desired interaction parameter in the allelic logistic regression model.

Wellek and Ziegler inspired statistic

The Wellek and Ziegler (WZ) inspired statistic is based on calculating the correlation coefficient (within cases and controls separately) between genotype variables x_g and x_h, coded (0,1,2) according to the number of susceptibility alleles at locus G and H respectively. We then test whether this correlation coefficient is the same in cases as in controls (case/control test) or whether the correlation coefficient in cases equals 0 (case-only test).

Statistical theory [2] predicts the following relationship between the sample correlation coefficient r_{gh} and the estimated regression coefficient $\hat{\beta}$ from standard linear (ordinary least squares) regression of a variable x_g (with observations $x_{g_i}; i = 1, \ldots, n$) regressed on a predictor variable x_h (with observations $x_{h_i}; i = 1, \ldots, n$):

$$\hat{\beta} = r_{gh} \frac{s_g}{s_h}$$

where s_g^2 and s_h^2 are the unbiased sample variances $s_g^2 = \sum_{i=1}^{n} (x_{g_i} - \overline{x}_g)^2$ and $s_h^2 = \sum_{i=1}^{n} (x_{h_i} - \overline{x}_h)^2$. In addition, $\hat{\beta}$ is equal to the maximum likelihood estimator of β obtained from fitting the following linear model to the observations:

$$x_g = \beta_0 + \beta x_h + \epsilon \qquad \text{where } \epsilon \sim N(0, \sigma^2)$$

This relationship between the correlation coefficient and the estimated regression coefficient suggests that the WZ test can be considered as a test of whether $\beta_A = 0$ (case-only test), or of whether $\frac{\beta_A s_{h_A}}{s_{g_A}} = \frac{\beta_N s_{h_N}}{s_{g_N}}$ (case/control test), where A and N refer to quantities calculated in cases and controls, respectively. Since x_g and x_h are discrete multinomial (rather than continuous) variables, linear regression is perhaps not the most obvious way to model their relationship, but nevertheless the regression coefficients β_A and β_B still correspond to the estimates of the slopes of the lines that minimise the sum of the squared differences between predicted and observed values of x_h, under this model.

Suppose the true model for disease corresponds to a standard saturated logistic regression model as given in Equation (2) of the main manuscript:

$$\begin{aligned}\log\frac{p}{1-p} &= \alpha + \beta_1 I(x_g = 1) + \beta_2 I(x_g = 2) + \gamma_1 I(x_h = 1) + \gamma_2 I(x_h = 2) + \delta_{11} I(x_g = 1) I(x_h = 1) \\ &\quad + \delta_{12} I(x_g = 1) I(x_h = 2) + \delta_{21} I(x_g = 2) I(x_h = 1) + \delta_{22} I(x_g = 2) I(x_h = 2)\end{aligned}$$

Under the null hypothesis of no interaction (i.e. $\delta_{jk} = 0 \quad \forall j, k$), assuming no population level correlation between the variables x_g and x_h, and making a rare disease assumption, we have

$$\begin{aligned}\frac{P(x_g|D, x_h)}{P(x_g = 0|D, x_h)} &= \frac{P(D|x_g, x_h)P(x_g|x_h)/P(D|x_h)}{P(D|x_g = 0, x_h)P(x_g = 0|x_h)/P(D|x_h)} \\ &= \frac{e^{\alpha+\beta_1 I(x_g=1)+\beta_2 I(x_g=2)+\gamma_1 I(x_h=1)+\gamma_2 I(x_h=2)} P(x_g)}{e^{\alpha+\gamma_1 I(x_h=1)+\gamma_2 I(x_h=2)} P(x_g = 0)} \\ &= [e^{\beta_1 I(x_g=1)+\beta_2 I(x_g=2)}] \frac{P(x_g)}{P(x_g = 0)}\end{aligned}$$

where D indicates the event that an individual is affected (diseased). Thus the odds (and therefore the multinomial probabilities) of a case falling into genotype categories 1 or 2 relative to category 0 at locus G do not, in fact, depend on the genotype at locus H, indicating that the observations in cases should be equally distributed between the three genotype categories ($x_g = 0, 1, 2$) at each level (0, 1, 2) of x_h. Therefore the expected value of the linear regression coefficient β_A (i.e. the slope), and thus r_{gh_A}, should be 0, validating use of the WZ case-only test in these circumstances. Similarly, in controls we obtain

$$\frac{P(x_g|\overline{D}, x_h)}{P(x_g = 0|\overline{D}, x_h)} = \frac{P(x_g)}{P(x_g = 0)}$$

indicating that the observations in controls should also be equally distributed between the three genotype

categories ($x_g = 0, 1, 2$) at each level (0, 1, 2) of x_h. Therefore the expected value of the linear regression coefficient β_N (and thus r_{gh_N}) should also be 0, validating use of the WZ case/control test in these circumstances.

If there is population-level correlation between the variables x_g and x_h, by a similar argument we obtain

$$\frac{P(x_g|D,x_h)}{P(x_g=0|D,x_h)} = [e^{\beta_1 I(x_g=1)+\beta_2 I(x_g=2)}]\frac{P(x_g|x_h)}{P(x_g=0|x_h)}$$

while in controls we obtain

$$\frac{P(x_g|\overline{D},x_h)}{P(x_g=0|\overline{D},x_h)} = \frac{P(x_g|x_h)}{P(x_g=0|x_h)}$$

If there were no main effects at locus G ($\beta_1 = \beta_2 = 0$), then these two quantities would be the same within cases and controls. Although the distribution into the three genotype categories for locus G ($x_g = 0, 1, 2$) will vary according to the genotype at locus H, resulting in a non-zero slope for the linear regression line, this slope should be the same for cases and controls. However, even if the slopes are the same, if there there are main effects at locus H, the quantity s_h and thus the correlation coefficient r_{gh} will not be the same in cases as in controls. Thus, the WZ case/control test is not expected to be valid in the presence of LD, unless there are no main effects operating at either locus. These results are consistent with what we observe in simulation Scenarios 1-4.

References

1. Purcell S, Neale B, Todd-Brown K, Thomas L, Ferreira MA, et al. (2007) PLINK: a tool set for whole-genome association and population-based linkage analyses. Am J Hum Genet 81: 559–575.

2. Draper NR, Smith H (1981) Applied Regression Analysis, 2nd Edition. John Wiley & Sons, New York.
