# Supplementary material for: Improved Statistics for Genome-Wide Interaction Analysis
Source: PLoS Genet. 2012 Apr 5;8(4):e1002625. doi: 10.1371/journal.pgen.1002625 (PMC3320596; doi:10.1371/journal.pgen.1002625)
Supplement: Text S5 — Here we show that the Wellek and Ziegler inspired case-only statistic can be viewed equivalently as a score test with respect to the interaction parameter δ in the model given in the second table on page 5 of Text S3. (PDF) [file pgen.1002625.s017.pdf]

Text S5

Here we show that the Wellek and Ziegler inspired case-only statistic can be viewed equivalently as a score test with respect to the interaction parameter δ in the model given in the second table on page 5 of Text S3. We repeat the table from page 5 of Text S3 here for completeness:

Paternal	Maternal haplotype			
haplotype	G_1-H_1	G_1-H_2	G_2-H_1	G_2-H_2
G_1-H_1	$e^{\alpha+2\beta+2\gamma+2\delta}$	$e^{\alpha+2\beta+\gamma+\delta}$	$e^{\alpha+\beta+2\gamma+\delta}$	$e^{\alpha+\beta+\gamma+\delta}$
G_1-H_2	$e^{\alpha+2\beta+\gamma+\delta}$	$e^{\alpha+2\beta}$	$e^{\alpha+\beta+\gamma}$	$e^{\alpha+\beta}$
G_2-H_1	$e^{\alpha+\beta+2\gamma+\delta}$	$e^{\alpha+\beta+\gamma}$	$e^{\alpha+2\gamma}$	$e^{\alpha+\gamma}$
G_2-H_2	$e^{\alpha+\beta+\gamma+\delta}$	$e^{\alpha+\beta}$	$e^{\alpha+\gamma}$	e^{α}

This model can be viewed as the model for estimating the LD parameter that is used in the Wu et al. case-only statistic with re-parameterization $P_{11} = e^{\delta+\beta+\gamma+\alpha/2}$, $P_{12} = e^{\gamma+\alpha/2}$, $P_{21} = e^{\beta+\alpha/2}$, $P_{22} = e^{\alpha/2}$.

The resulting penetrance table corresponding to the observable genotypes is given as follows:

	Locus H		
Locus G	H_1H_1	H_1H_2	H_2H_2
G_1G_1	$e^{2\delta+2\beta+2\gamma+\alpha}$	$2e^{\delta+2\beta+\gamma+\alpha}$	$e^{2\beta+\alpha}$
G_1G_2	$2e^{\delta+\beta+2\gamma+\alpha}$	$2e^{\beta+\gamma+\alpha}(e^{\delta}+1)$	$2e^{\beta+\alpha}$
G_2G_2	$e^{2\gamma+\alpha}$	$2e^{\gamma+\alpha}$	e^{α}

Using the notation in Table 1 of the main manuscript, the log-likelihood function assuming the model in the above table is written as

$$2\delta n_{22} + \delta n_{21} + \delta n_{12} + \log(e^{\delta}+1)n_{11} + n\alpha + C,$$

where C denotes a constant independent of δ and n is the total number of observed genotypes. The parameter α plays a role as the normalizing factor for standardization of the genotype frequencies. That is, the summation of all genotype frequencies in the above table has to be unity:

$$\begin{aligned}1 &= e^{\alpha}\{e^{2\delta+2\beta+2\gamma} + 2e^{\delta+2\beta+\gamma} + e^{2\beta} + 2e^{\delta+\beta+2\gamma} + 2e^{\beta+\gamma}(e^{\delta}+1) + 2e^{\beta} + e^{2\gamma} + 2e^{\gamma} + 1\} \\ &= e^{\alpha}(e^{\delta+\beta+\gamma} + e^{\beta} + e^{\gamma} + 1)^2\end{aligned}$$

Taking the logarithm and rearranging leads to

$$\alpha = -2\log(e^{\delta+\beta+\gamma} + e^{\beta} + e^{\gamma} + 1)$$

By noting that α is a function of (β, γ, δ) and differentiating the log-likelihood function with respect to δ, we have the score function with respect to the parameter δ:

$$2n_{22} + n_{21} + n_{12} + n_{11}\frac{e^{\delta}}{e^{\delta}+1} - 2n\frac{e^{\delta+\beta+\gamma}}{e^{\delta+\beta+\gamma} + e^{\beta} + e^{\gamma} + 1} = 2n_{22} + n_{21} + n_{12} + n_{11}\frac{e^{\delta}}{e^{\delta}+1} - 2ne^{\delta+\beta+\gamma+\alpha/2},$$

(where we use the relationship $e^{-\alpha/2} = e^{\delta+\beta+\gamma} + e^{\beta} + e^{\gamma} + 1$). If $\delta = 0$ (the null hypothesis for the case-only test) the score function reduces to

$$2n_{22} + n_{21} + n_{12} + \frac{1}{2}n_{11} - 2ne^{\beta+\gamma+\alpha/2}$$

Furthermore, since $e^{\gamma+\alpha/2} = P_{12} = pv$, $e^{\beta+\alpha/2} = P_{21} = qu$ and $e^{\alpha/2} = P_{22} = qv$, we have $e^{\gamma} = P_{12}/P_{22} = p/q$. Therefore, $e^{\beta+\gamma+\alpha/2} = \frac{p}{q}qu = pu$ and the score function at $\delta = 0$ reduces to

$$\frac{n}{2}\left(4\frac{n_{22}}{n} + 2\frac{n_{21}}{n} + 2\frac{n_{12}}{n} + \frac{n_{11}}{n} - 4pu\right) \tag{1}$$

Let X and Y denote, as in [1], random variables corresponding to Wellek and Ziegler's genotype coding $(2, 1, 0)$ for locus G and H. The empirical means for X and Y are then expressed as

$$\frac{1}{n}\sum_{i=1}^{n}\{2I(X_i = 2) + I(X_i = 1)\} = 2\frac{n_{22} + n_{21} + n_{20}}{n} + \frac{n_{12} + n_{11} + n_{10}}{n},$$
$$\frac{1}{n}\sum_{i=1}^{n}\{2I(Y_i = 2) + I(Y_i = 1)\} = 2\frac{n_{22} + n_{12} + n_{02}}{n} + \frac{n_{21} + n_{11} + n_{01}}{n},$$

if (X_i, Y_i) represents the realization of (X, Y) for ith individual. The first and second quantities equal $2\hat{p}$ and $2\hat{u}$, in which $\hat{p}$ and $\hat{u}$ are MLEs of the allele frequencies p and u. Hence, $4\hat{p}\hat{u}$ is the product of the sample means of X and Y.

Similarly, the fact that $n_{jk} = \sum_{i=1}^{n} I(X_i = j, Y_i = k)$ for $j, k = 1, 2$ implies that the term $(4n_{22} + 2n_{21} + 2n_{12} + n_{11})/n$ is also interpreted as the sample expectation $E(XY)$. With this notation, the score function at $\delta = 0$ substituting the MLE in Equation (1) can be viewed as the sample covariance

$\text{cov}(X, Y) = E(XY) - E(X)E(Y)$ multiplied by $n/2$, under the Wellek and Ziegler coding. Because the hypothesis that the covariance equals zero is equivalent to the hypothesis that the correlation equals zero (if variances are nonzero), the Wellek and Ziegler inspired test that corresponds to the test for the correlation coefficient being zero is equivalent to the score test for δ in the model described above.

References

1. Wellek S, Ziegler A (2009) A genotype based approach to assessing the association between single nucleotide polymorphisms. Hum Hered 67: 128–139.
